# NOTCH signaling in COVID-19: a central hub controlling genes, proteins, and cells that mediate SARS-CoV-2 entry, the inflammatory response, and lung regeneration

**DOI:** 10.3389/fcimb.2022.928704

**Published:** 2022-08-04

**Authors:** Piyush Baindara, Md Bodruzzaman Sarker, Alexander P. Earhart, Santi M. Mandal, Adam G. Schrum

**Affiliations:** ^1^ Department of Molecular Microbiology & Immunology, School of Medicine, University of Missouri, Columbia, MO, United States; ^2^ Division of Animal Sciences, College of Agriculture, Food and Natural Resources, University of Missouri, Columbia MO, United States; ^3^ Central Research Facility, Indian Institute of Technology Kharagpur, Kharagpur, India; ^4^ Department of Surgery, School of Medicine, University of Missouri, Columbia, MO, United States; ^5^ Department of Biomedical, Biological, & Chemical Engineering, College of Engineering, University of Missouri, Columbia, MO, United States

**Keywords:** ADAM, ACE2, furin, Notch, SARS-CoV-2, COVID-19, gamma-secretase inhibitor (GSI)

## Abstract

In the lungs of infected individuals, the downstream molecular signaling pathways induced by Severe Acute Respiratory Syndrome Coronavirus 2 (SARS-CoV-2) are incompletely understood. Here, we describe and examine predictions of a model in which NOTCH may represent a central signaling axis in lung infection in Coronavirus Disease 2019 (COVID-19). A pathway involving NOTCH signaling, furin, ADAM17, and ACE2 may be capable of increasing SARS-CoV-2 viral entry and infection. NOTCH signaling can also upregulate IL-6 and pro-inflammatory mediators induced to hyperactivation in COVID-19. Furthermore, if NOTCH signaling fails to turn down properly and stays elevated, airway regeneration during lung healing can be inhibited—a process that may be at play in COVID-19. With specific NOTCH inhibitor drugs in development and clinical trials for other diseases being conducted, the roles of NOTCH in all of these processes central to both infection and healing merit contemplation if such drugs might be applied to COVID-19 patients.

## Introduction

SARS-CoV-2 infection has been reported to cause various pathologies in respiratory, cardiovascular, gastrointestinal, and central nervous systems with a prominent cause of death being Acute Respiratory Distress Syndrome (ARDS) secondary to COVID-19 (Long et al., 2020; Paybast et al., 2020; Holliday et al., 2021; Hu et al., 2021a). Cellular entry of SARS-CoV-2 is mediated by its spike protein upon interaction with the human receptor ACE2 (Walls et al., 2020). Protease activities that affect viral entry include those of furin, which cleaves spike protein and facilitates entry. In SARS-CoV-2 target cells, ADAM17 can induce ectodomain shedding of ACE2 inhibiting viral engagement and entry, while TMPRSS2 cleaves ACE2 but enhances SARS-CoV-2 infection, highlighting that regulation of ACE2 cleavage and shedding is a complex process incompletely understood (Hoffmann et al., 2020; Zipeto et al., 2020; Wang et al., 2022).

Greater understanding of host signaling pathways important for infection might point toward novel viral or host targets with therapeutic potential for COVID-19. NOTCH signaling is highly evolutionarily conserved and plays a role as master regulator of various biological functions in health and disease (Shang et al., 2016). In terms of viral pathogenesis, NOTCH signaling has previously been shown to regulate different processes in infection by Epstein-Barr virus, influenza virus, respiratory syncytial virus, human papillomavirus, hepatitis B virus, and human immunodeficiency virus (Hayward, 2004; Breikaa and Lilly, 2021). Recently, up-regulation of NOTCH signaling was implicated by transcriptional signatures obtained from the lungs of SARS-CoV-2-infected rhesus macaques (Rosa et al., 2021). In the present study, we describe and examine predictions of a model where NOTCH signaling may represent a central hub controlling genes, proteins, and cells in SARS-CoV-2 infection, and in the resulting processes of inflammation and tissue regeneration in the lung.

### Rizzo’s model where NOTCH impacts SARS-CoV-2 infection and inflammation

Expressed at the surface membrane, four human NOTCH receptors (NOTCH 1-4) can be engaged by Jagged-1, Jagged-2, and Delta-like ligands (Dll)1, 3, and 4 (Aster et al., 2017; Sprinzak and Blacklow, 2021). Recently, we were highly interested in a model that was proposed, described, and illustrated by Paola Rizzo and colleagues (Rizzo et al., 2020), placing NOTCH as a candidate regulatory pathway in SARS-CoV-2 infection and inflammation. Here, we first concisely summarize main points of the Rizzo model, building on illustrations inspired from that seminal publication. Next, we extend the model to potential roles for NOTCH in adaptive immunity and tissue regeneration in the lung. With key elements of the model described, we test predictions of the model accessing publicly available single-cell gene expression data from human lungs. In the polygenic, multi-ligand, post-translationally regulated NOTCH family system, so many different colors of response are theoretically possible; yet, we will allow an oversimplified framework tool assignment of “NOTCH good” versus “NOTCH bad” to represent predicted net effects on disease outcome upon NOTCH pathway activation. Finally, we consider the extent to which drug-mediated NOTCH inhibition should be contemplated for severe COVID-19 that is unresponsive to other therapies.

### NOTCH bad #1

**Figure 1:**
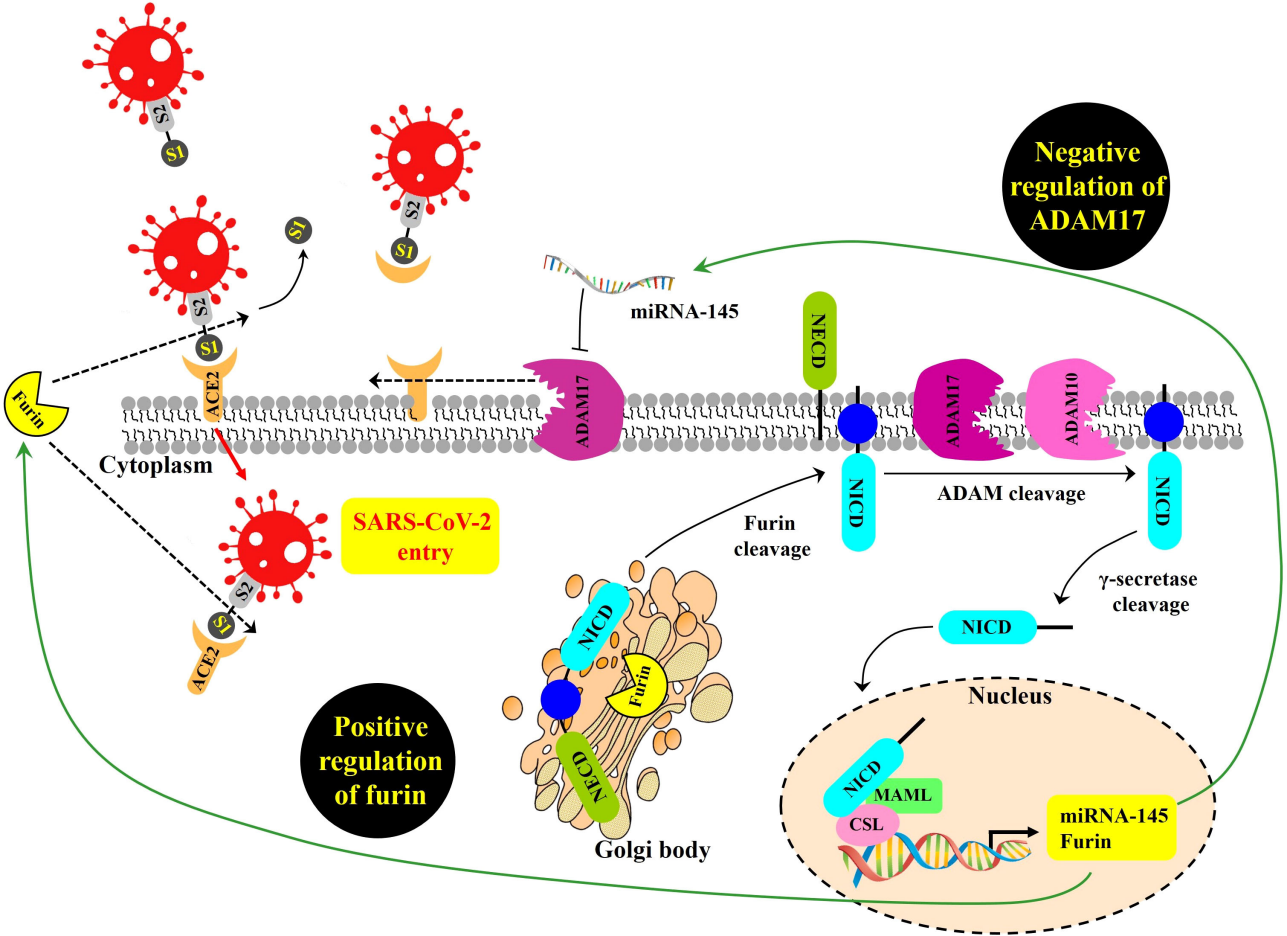
Proposed NOTCH signaling pathway effects on SARS-CoV-2 infection, modeled from Rizzo et al. (2020). SARS-CoV-2 entry is mediated by the binding of the viral spike (S) glycoprotein to ACE2 and proteolytic cleavage at the S1/S2 site of the S glycoprotein by the host protease furin. ADAM17 regulates ACE2 levels on the plasma membrane by promoting ACE2 shedding. NOTCH signaling is a positive regulator of furin and a negative regulator of ADAM17 transcript, the latter due to induction of miRNA-145. GSIs are NOTCH inhibitors that may represent a possible therapeutic strategy to inhibit viral entry into cells by reducing furin and increasing ADAM17-mediated ACE2 shedding. NOTCH precursor is cleaved by furin in the Golgi apparatus to form a heterodimer of NOTCH extracellular domain (NECD), plus the remaining cleavage product containing within the NOTCH intracellular domain (NICD). This cleavage-induced heterodimer is exported to surface expression. Upon productive ligand engagement, NOTCH is cleaved by ADAM10 or ADAM17 and later cleaved by the gamma-secretase complex. Afterwards, liberated NICD translocates into the nucleus and interacts with the transcription factor CSL and the transcriptional co-activator MAML to regulate the transcription of NOTCH target genes, including furin, and miRNA-145.

First, for cells directly infected by SARS-CoV-2, the NOTCH pathway might promote viral entry (**Figure 1**). Beginning at the cell surface of a potential target of SARS-CoV-2, NOTCH activation by ligand engagement leads to the sequential proteolytic cleavage of NOTCH by protease ADAM10 or ADAM17, and then by the gamma-secretase complex (Qiu et al., 2015; Breikaa and Lilly, 2021; Sprinzak and Blacklow, 2021). Liberated NOTCH intracellular domain (NICD) translocates to the nucleus where it joins CBF-1/RBP-Jk/Suppressor of hairless/Lag-1 (CSL) and mastermind-like (MAML) in a transcriptional complex inducing expression of many target genes, including furin and miRNA-145 (Rizzo et al., 2020). As a protein convertase, furin cleaves immature NOTCH protein to its mature heterodimeric form prior to surface expression (van Tetering and Vooijs, 2011), and also cleaves SARS-CoV-2 spike protein in a process required for its efficient cellular entry during infection (Peacock et al., 2021; Wu and Zhao, 2021). Therefore, by increasing furin levels, NOTCH signaling might promote positive-feedback of more NOTCH surface expression and concomitantly increase viral spike cleavage and cellular entry.

NOTCH signaling might also make more cell surface ACE2 available for SARS-CoV-2 docking. ADAM17 is a main convertase involved in the proteolytic cleavage and ectodomain shedding of ACE2 during SARS-CoV-2 infection (Palau et al., 2020). At the mRNA level, ADAM17 expression can be inhibited when NOTCH upregulates miRNA-145 (Doberstein et al., 2013). Thus, it is possible that NOTCH could reduce ADAM17 levels, resulting in increased non-cleaved, surface-retained ACE2 available for spike-mediated viral entry.

### NOTCH bad #2

**Figure 2:**
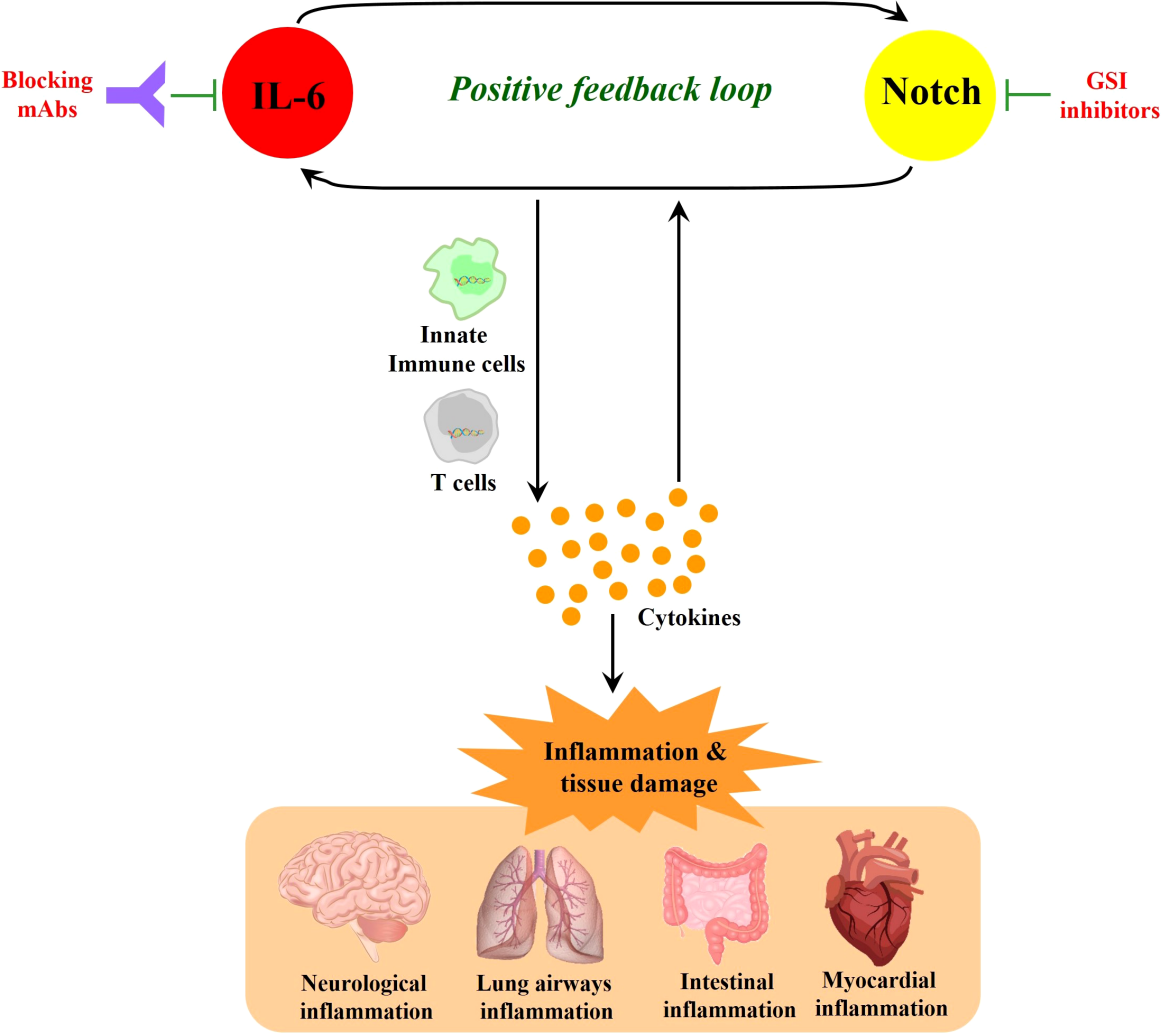
NOTCH signaling induces IL-6 in a positive-feedback loop. NOTCH signaling promotes the production of inflammatory cytokines including IL-6. In turn, IL-6 increases the expression of NOTCH ligands (such as Dll1 and Dll4), which can further amplify NOTCH signaling. This NOTCH : IL-6 positive-feedback loop can promote innate immune cell and adaptive immune T cell activation together with further amplified inflammatory cytokine elaboration. When these processes are highly active without sufficient downregulation in a resolution phase, they can cause inflammatory tissue damage and continued cycles of IL-6 and NOTCH upregulation and activation. While monoclonal antibodies (mAbs) that block IL-6 or its receptor represent promising therapeutic modalities, GSIs block NOTCH signaling as candidate drugs in clinical trials for other diseases, and these latter compounds could be contemplated when severe COVID-19 may be unresponsive to other therapies. In sum, it is suggested that targeting the positive feedback loop between NOTCH and IL-6 could be a potential therapeutic strategy that may have application against COVID-19.

In the immune response to SARS-CoV-2 infection, excessive NOTCH signaling may promote IL-6 and inflammatory pathways that can exacerbate morbidity and severity of COVID-19 (**Figure 2**). Hyperactivation of innate immune cells and products contribute to cytokine storm (Hu et al., 2021b) and disease severity in the lung airway, gastrointestinal tract, and myocardial and neurological tissues (Najjar et al., 2020; Pezzini and Padovani, 2020). IL-6 is a major cytokine involved in the immunopathophysiology of COVID-19, a characteristic biomarker of cytokine storm and a therapeutic target to reduce inflammation and disease symptoms (Campochiaro and Dagna, 2020; Grifoni et al., 2020; Santa Cruz et al., 2021). Additionally, COVID-19-induced cytokine storm syndrome often includes high IL-6 receptor, TNF-α, granulocyte-colony stimulating factor and other immune products (Zhang et al., 2020).

NOTCH1 can directly bind to the IL-6 promoter to activate its transcription (Wongchana and Palaga, 2012). In turn, IL-6 increases the expression of NOTCH ligands such as Dll1 and Dll4, enacting a potential positive feedback loop (Yang et al., 2015; Hildebrand et al., 2018; Rizzo et al., 2020). NOTCH signaling also triggers the production of iNOS and numerous cytokines including IL-4, IL-13, plus other inducers of JNK and ERK MAP kinase pathways that can ultimately influence downstream T cell differentiation (Qu et al., 2017; Wang et al., 2018; Sega et al., 2019). TNF-α has also been reported to induce expression of NOTCH1 and NOTCH4 (Quillard et al., 2010) and high serum TNF-α levels are associated with increased mortality in severe COVID-19 (Mortaz et al., 2021). In a recent cohort study, TNF-α inhibitor monotherapy was found to reduce risk in severe COVID-19 patients (Izadi et al., 2021). These observations are consistent with a contributing role for NOTCH in the inflammatory response known to promote severity in COVID-19.

### NOTCH good #1

NOTCH activation can influence peripheral CD4 T helper cell responses (Adler et al., 2003; Eagar et al., 2004; Osborne and Minter, 2007), thus potentially aiding adaptive immune protection, memory generation, and antibody production for sterilizing immunity in the long run. The expression of this potentially positive effect might be observed subsequent to the more dangerous, early, hyper-innate immune signaling imbalance (**Figure 2**) that precedes the zenith of a fully mounted adaptive immune protective response. But this illustrates one means by which NOTCH signaling in certain specific cells, like T cells, perhaps at the right time in the response kinetic, may be predicted to promote a positive effect against SARS-CoV-2 infection.

### NOTCH good #2

**Figure 3:**
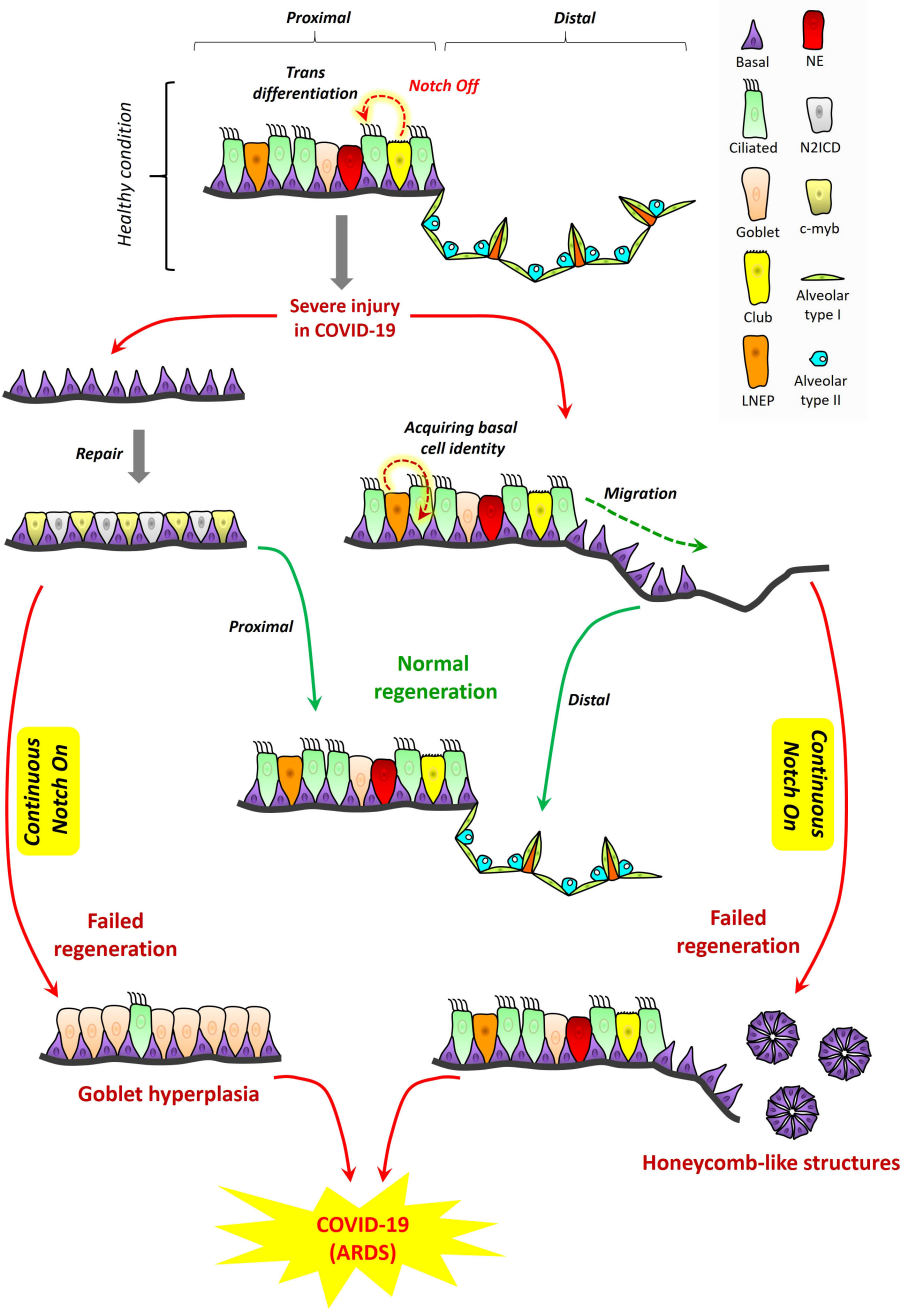
Proposed role for NOTCH in lung regeneration in COVID-19: tissue structure in the proximal and distal respiratory tract. A variety of lung epithelial cells exist along the proximal‐distal axis. In proximal regions, there are five major cell populations, which include basal cells (purple), goblet cells (light pink), ciliated cells (green), club cells (yellow), and neuroendocrine cells (red). LNEPs (lineage‐negative epithelial progenitor cells) are shown in light orange color. In alveolar regions, there are mainly two types of epithelial cells, flattened alveolar type I cells (light green) and cuboidal alveolar type II cells (indigo). Septa are shown in dark orange color. Cell loss in the proximal respiratory tract due to injury triggers the expansion of basal cells and their direct segregation into N2ICD (club progenitor, light grey) and c‐myb (ciliated progenitor, light yellow) cells. However, continuous NOTCH activation during COVID-19 along with cytokine storm may result in goblet cell hyperplasia which could exacerbate disease pathogenesis and contribute to ARDS. LNEPs acquire basal cell identity and migrate towards the damaged area of the distal respiratory tract. NOTCH blocks the trans-differentiation of basal cells into alveolar cells and thus NOTCH inhibition is necessary for normal regeneration. However, continuous NOTCH activation may occur in COVID-19 and this could result in abnormal regeneration with basal cell excess and honeycomb‐like structures that may contribute to ARDS and disease severity.

In homeostasis and healing post-injury, NOTCH signaling is essential for tissue regeneration in lung and it is thus conceivable this principle may apply to ARDS in COVID-19. The proximal-distal axis of the human respiratory tract consists of a variety of lung epithelial cells (**Figure 3**). The trachea, bronchi, and bronchioles are proximal regions of the respiratory system that are made up of basal cells, goblet cells, ciliated cells, club cells, and neuroendocrine cells. Basal cells reside in both cartilaginous and smaller airways that include bronchioles. The alveolar or distal regions consist of flattened alveolar type I cells (AT1) and cuboidal alveolar type II cells (AT2) (Kiyokawa and Morimoto, 2020). NOTCH signaling promotes proliferation and dissemination of basal cells necessary for healing and regeneration of other lung cell types to restore functional airways (Kotton and Morrisey, 2014).

### NOTCH bad #3

Excessive NOTCH signaling inhibits regeneration of cell types necessary for airway function (**Figure 3**). Subsequent to NOTCH signaling, its tightly regulated inhibition is critical for normal maintenance of airway cell subsets and their regeneration during healing. Upon alveolar epithelial injury, NOTCH signaling in AT2 cells becomes downregulated by a non-canonical ligand of NOTCH (Dlk1), facilitating AT2 to AT1 transition, and leading to alveolar repair (Finn et al., 2019). NOTCH inhibition is required to promote diverse cell differentiation in airway replenishment and restoration while avoiding lung fibrosis during healing (Wasnick et al., 2019). Continuous or excessive NOTCH activation can perturb the balance of cell differentiation during regeneration, promoting basal cell proliferation and goblet cell development at the expense of other diverse airway cell types. In the proximal airways, excessive NOTCH activation can promote goblet cell hyperplasia accompanied by high cytokine levels (Danahay et al., 2015). In the distal area, continuous NOTCH blocks the trans-differentiation of basal cells into alveolar cells (Kiyokawa and Morimoto, 2020), while inhibiting AT2 to AT1 transition, resulting in failure to regenerate the alveolus, an outcome sometimes marked by honeycomb cysts and basal cell excess (Vaughan et al., 2015; Reyfman et al., 2019).

Some published observations support the notion that these patterns of NOTCH activation and inhibition may be at play in COVID-19, at least in subsets of patients with underlying chronic lung conditions. SARS-CoV-2 shows gradient infectivity from proximal to distal regions of the respiratory tract that follows ACE2 expression patterns, with ciliated cells and AT2 cells being primary targets (Hou et al., 2020) in addition to goblet cells (Osan et al., 2020). As infection progresses, SARS-CoV-2 cell tropism expands to infect a greater array of airway epithelial cells including club and basal cells (Ryu and Shin, 2021). It has been previously proposed that goblet cell hyperplasia could occur in COVID-19 and other viral infections known to induce high mucus production (Mehedi, 2020; Cortez and Schultz-Cherry, 2021). An *in vitro* model of airway epithelium derived from healthy or COPD subjects suggested that goblet cell hyperplasia may have been involved in SARS-CoV-2 infection and COVID-19 disease progression (Osan et al., 2020). Other case studies of COVID-19 patients with pulmonary fibrosis reported the presence of honeycomb-like structures with speculation these might impact infection or recovery (Combet et al., 2020; Zou et al., 2021). One case report of COVID-19 showed, among the pathological changes in lung epithelium, the presence of plugged bronchioles with mucus and goblet cell hyperplasia (Yin et al., 2021). It seems reasonable to suggest that NOTCH’s role in lung regeneration is likely to be important in patients who have underlying chronic lung conditions among the highest risk of ARDS secondary to severe COVID-19. To propose that a similar role may be important in the general patient population may be logical, but we are unaware of empirical evidence that directly assesses this concept to date. Therefore, we hypothesize a model where it is conceivable that known rules of NOTCH signaling and downregulation might be at play in COVID-19, where excessive NOTCH activity might inhibit healthy regeneration and increase ARDS severity in COVID-19.

### Testing predictions of the model using publicly available single-cell gene expression data

We reviewed publicly accessible single-cell (sc)RNA-seq data from healthy human lungs (Vieira Braga et al., 2019), assuming these data would represent gene expression patterns of critical cell types early in or near the start of SARS-CoV-2 infection. The first goal was to determine if the model in **Figure 1** could be rejected, unless single cell types could be found to co-express the key genes in cis required for the model to remain sustainable. Supplemental figures each include gene expression data on two pages: (a) epithelial airway cell types and (b) non-epithelial cell types including leukocytes.

First, we assessed whether NOTCH genes were expressed by cell types that could impact SARS-CoV-2 infection. NOTCH1 and NOTCH2 were expressed by basal, alveolar, club, goblet, and ciliated cells, as well as numerous non-epithelial cells (**Figures S1, S2**), although NOTCH1 was relatively greater in the airway than parenchyma in basal2 cells, with an opposite trend for NOTCH2 which favored parenchyma over airway expression in basal1 cells. NOTCH3 expression was largely in basal2, club, and goblet cells with little non-epithelial expression (**Figure S3**). NOTCH4 was mostly expressed by basal1 cells in lung airways along with some ciliated cell expression (**Figure S4**). We observed that another human-specific gene, NOTCH2NL, was mostly expressed by goblet cells in lung airways followed by type1 alveolar cells in the lung parenchyma (**Figure S5**). Similarly, we checked the expression pattern of NOTCH receptors in different cell types. We observed that NOTCH ligand dll1 was expressed mostly by basal cells in lung parenchyma and lung airways, along with club and ciliated cell expression in airways (**Figure S6**), while dll3 and dll4 showed sparse expression (**Figures S7, S8**). Other NOTCH receptors, Jag1 and Jag2, were found highly expressed by airway epithelial cell types, with Jag1 expression was also well-represented in leukocytes (**Figures S9, S10**). Additionally, all the components of the gamma-secretase complex (PSENEN, PSEN1, PSEN2, NCSTN, APH1A, and APH1B) were expressed in lung airways, lung parenchyma, and leukocytes (**Figures S11-S16**). These observations showed that genes relevant to NOTCH signaling have widespread representation in expression levels present in cell types relevant to SARS-CoV-2 infection in the lung.

Next, we checked the gene expression profiles of ACE2, furin, TMPRSS2, ADAM17, and ADAM10. ACE2 was concentrated in goblet and ciliated2 cells, with expression also among ciliated1, club, basal, and alveolar cells (**Figure S17**). Furin and TMPRSS2 expression was observed in all epithelial subsets weighted toward parenchyma and furin also displayed clear expression in non-epithelial subsets, including leukocytes (**Figures S18, S19**). ADAM17 and ADAM10 showed expression in all epithelial and non-epithelial subsets (**Figures S20, S21**). Because these data show that the same cell types can co-express NOTCH, ACE2, furin, TMPRSS2, and ADAM proteases, it validates the possibility that these gene products could act in cis and affect each other as required by the model (**Figure 1**).

Elements of the model in **Figures 2**, **3**, are less reliant on predictions of gene expression in single cell types in cis and rather involve signaling and/or differentiation between cell types. In lung epithelial cell types, IL-6 showed sparse but clear expression spread across all subsets, while greater relative expression was seen in leukocytes and other non-epithelial cells (**Figure S22**). Expression of receptors for IL-6 (IL-6R and IL6ST) was observed across all cell types (**Figures S23,S24**), indicating that a feedback loop between IL-6 responsiveness and NOTCH signaling remains a possible feature which might contribute to the inflammatory response during COVID-19.

### Contemplating NOTCH-based inhibitor drugs for severe, COVID-19 that is unresponsive to other treatments

**Figure 4:**
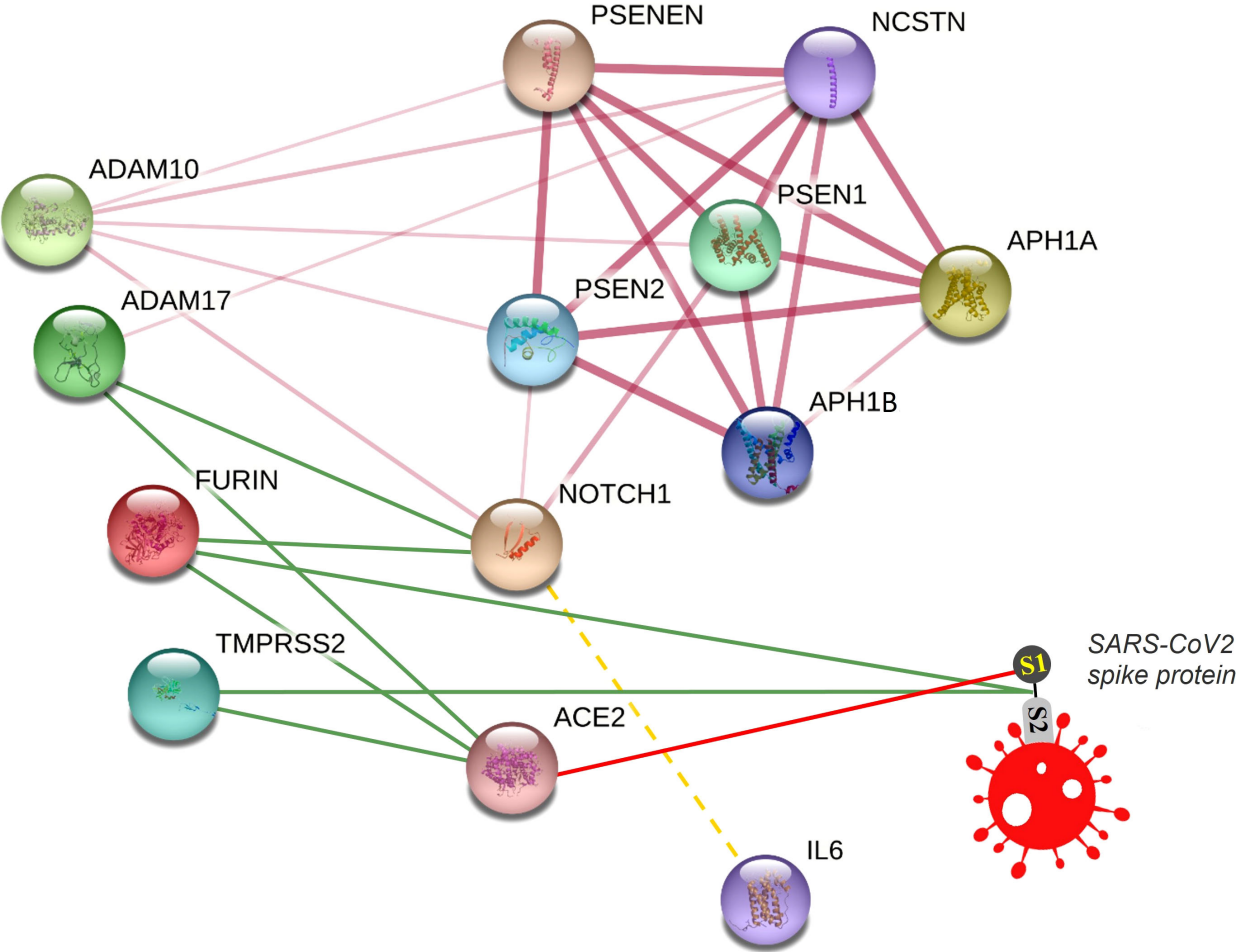
Connecting NOTCH and SARS-CoV-2 in a molecular interaction network. This network is partially generated by STRING v11. All STRING-generated edges are shown in pink (light or dark), while other edges are drawn manually. Gamma secretase complex is represented by different subunits including APH1A, APH1B, PSEN1, PSEN2, PSENEN, and NCSTN. Other proteins included are NOTCH1, FURIN, ADAM17, ADAM10, TMPRSS2, and ACE2. The SARS-CoV2 spike protein is drawn manually. Interaction edges of some proteases (ADAM17, FURIN, TMPRSS2) with NOTCH and ACE2 are indicated by green lines, while binding of SARS-CoV-2 spike protein with its receptor ACE2 is indicated by a red line. The transcriptional (not protein) interaction of NOTCH and IL-6 is indicated by a dashed yellow line.

We visualized a molecular interaction network connecting NOTCH signaling with SARS-CoV-2 (**Figure 4**). Inhibition of NOTCH signaling, NOTCH : IL-6 positive feedback loop, or downstream molecules in these pathways might be hypothesized to decrease SARS-CoV-2 infection, downregulate the inflammatory response, and/or favor lung regeneration.

NOTCH represents a complicated pathway. At the molecular level, it is polygenic, multi-ligand, and post-translationally regulated. At the cell and tissue level, it has multiple different effects including opposing effects on many different cell types in development, activation, differentiation, proliferation, and regeneration. Therefore, it is possible that pharmacologic targeting of NOTCH could generate unwanted on-target, off-disease side-effects. However, it is also true that other pathways targeted in therapy, for example, those involving cortisol or corticosteroid, may also interact with and potentially effect a similar list of processes in multiple cell types and tissues. Yet with the right timing and at the right dose, corticosteroid improves outcomes of severe COVID-19 and is administered as part of standard care (Li et al., 2021). We wonder if NOTCH-based inhibitor drugs should be considered as clinical trial candidates for severe COVID-19 when patient subset risk or other factors make outcomes unresponsive to current established therapies.

Still, prior to discussing specific NOTCH inhibitor treatment ideas, more deliberation is provided here to consider the extent to which this could be foreseen as beneficial versus potentially harmful. In the present study, we counted issues where a net effect of NOTCH signaling could be predicted to be “bad” or “good”. Despite the oversimplification of this scoring tool, there is a pattern worth noting: the two processes scored as “good” are deemphasized in acute, severe disease, whereas the three processes scored as “bad” occur during acute, severe disease, and are here hypothesized to play a causal role contributing to severity. Therefore, if the present model were correct, then inhibition of NOTCH signaling in severe, COVID-19 that is unresponsive to other therapies might (a) decrease furin expression, decrease NOTCH expression, increase ADAM17 expression, and thus decrease new avenues of SARS-CoV-2 infection; (b) decrease inflammation by inhibiting NOTCH : IL-6 positive feedback loop; and (c) favor regeneration of alveoli and healing of respiratory apparatus. In contrast, predictable negative side-effects of NOTCH inhibition in this scenario could include (d) inhibiting CD4 helper T cells, which eventually are needed for adaptive immunity including optimal antibody generation from helped B cells; and (e) inhibiting a basic pathway required for lung tissue regeneration by precursor cells. Regarding (d), other treatments such as corticosteroid can have a similar effect and thus timing and dosage are critical in order to maximize benefit while minimizing this risk. Regarding (e), this might be an acceptable risk, perhaps briefly during intensive care, if NOTCH inhibition might provide the break that is needed to boost alveolar regeneration, even if more distal precursor cells were to be temporarily inhibited. Upon identifying these specific issues where NOTCH impacts SARS-CoV-2-mediated disease in the lungs, we propose that NOTCH inhibitory drug strategies are worth contemplating for severe COVID-19 that may be unresponsive to other therapies.

The data and studies summarized here could suggest a strategy for a subset of patients for whom a targeted NOTCH-based diagnostic assay could be developed and tested for technical efficacy as well as relevance to disease severity. Gamma secretase inhibitor (GSI) drugs have been developed to the point of various clinical trials for other indications (Espinoza and Miele, 2013; Yuan et al., 2015). We suggest that GSIs, provided for a finite period, might be beneficial for the treatment of severe COVID-19 that is unresponsive to other therapies. Regarding the route of administration, perhaps nebulization for direct delivery to the lungs may maximally target the infection and regeneration pathways discussed herein, while systemic delivery might have a more far-reaching effect to inhibit the NOTCH : IL-6 positive feedback loop in immune cells, and might also be predicted to cause more on-target, off-disease side-effects.

## Concluding remarks

We present a model where NOTCH may be a central hub regulating the processes of infection, inflammation, and lung regeneration in COVID-19. In outlining specific aspects of this model with prior knowledge documentation, and testing some predictions using public sc-RNA-seq data, we did not reject the model and found gene expression characteristics in the lung to be largely compatible. NOTCH activation might cause an increase in SARS-CoV-2 cell entry and infection, exacerbate inflammation in a NOTCH : IL-6 positive feedback loop, and inhibit functional airway regeneration. It is conceivable that NOTCH inhibitory drug candidates, such as GSIs, might improve these outcomes in severe COVID-19 that is unresponsive to other therapies.

## Data availability statement

Publicly available datasets were analyzed in this study. This data can be found here: Lung Cell Atlas (https://asthma.cellgeni.sanger.ac.uk/).

## Ethics statement

The present work uses publicly available human gene expression data .

## Author contributions

PB, MBS, AE, and AS collected, analyzed, and/or interpreted data. PB and AS wrote the manuscript. PB, SM, and AS led ideation, hypothesis generation, and conclusions. All authors contributed to the article and approved the submitted version.

## Funding

This work was supported by University of Missouri Biomedical Innovation recruitment funds and NIH grant R01GM103841 (AGS).

## Acknowledgment

We thank Kimberly G. Laffey, PhD, and Zachary M. Holliday, MD, for the critical review of this manuscript.

## Conflict of interest

The authors declare that the research was conducted in the absence of any commercial or financial relationships that could be construed as a potential conflict of interest.

## Publisher’s note

All claims expressed in this article are solely those of the authors and do not necessarily represent those of their affiliated organizations, or those of the publisher, the editors and the reviewers. Any product that may be evaluated in this article, or claim that may be made by its manufacturer, is not guaranteed or endorsed by the publisher.
