# Supplementary material for: NOTCH signaling in COVID-19: a central hub controlling genes, proteins, and cells that mediate SARS-CoV-2 entry, the inflammatory response, and lung regeneration
Source: Front Cell Infect Microbiol. 2022 Aug 4;12:928704. doi: 10.3389/fcimb.2022.928704 (PMC9386183; doi:10.3389/fcimb.2022.928704)
Supplement: Supplementary file 1 [file DataSheet_1.pdf]

NOTCH signaling in COVID-19: A central hub controlling genes, proteins, and cells that mediate SARS-CoV-2 entry, the inflammatory response, and lung regeneration

Piyush Baindara[1]*, Md Bodruzzaman Sarker[1,2], Alexander P. Earhart[1], Santi M. Mandal[3]*, Adam G. Schrum[1,2,4,5]*

[1]Department of Molecular Microbiology & Immunology, School of Medicine, University of Missouri, Columbia, MO, USA

[2]Division of Animal Sciences, College of Agriculture, Food and Natural Resources, University of Missouri, Columbia USA

[3]Central Research Facility, Indian Institute of Technology Kharagpur, Kharagpur 721302, India

[4]Department of Surgery, School of Medicine, University of Missouri, Columbia, MO, USA

[5]Department of Biomedical, Biological, & Chemical Engineering, College of Engineering, University of Missouri, Columbia, MO, USA

***Correspondence**

Piyush Baindara, Email: pbaindara@health.missouri.edu

Santi M. Mandal, Email: mandalsm@gmail.com

Adam Schrum, Email: schruma@health.missouri.edu

Supplemental Information

Molecular interaction network generated using STRING v11.0

The protein-protein interaction (PPI) network of 12 human proteins was made using STRING (version 11.0) (Szklarczyk et al., 2019) to assess potential for molecular and pathway interaction in SARS-CoV-2 infection. For this network, PPI enrichment p-value is $< 1.0^{-16}$, with an average node degree of 3.67. Pink, green, and red edges indicate that proteins are part of a PPI network, where pink indicates non-enzymatic, non-viral protein-protein interaction, green indicates protease:substrate interaction, and red indicates viral:cellular interaction. Pink thickness indicates the strength of data support (confidence). The minimum required interaction score is medium confidence that is 0.400 (set threshold). STRING generated 22 pink edges (light or dark) with a confidence score of 0.400 or above and an average local clustering coefficient of 0.562 indicative of good interaction between selected proteins. The clustering coefficient is a measure of how nodes are connected in the network. Outside the 12 nodes of the PPI network, IL-6 and SARS-CoV-2 with its spike protein S1 and S2 domains were added to the illustration. The dashed yellow edge connecting NOTCH and IL-6 represents gene expression influence, not protein interaction.

Single-cell (sc)RNA-seq data visualization

Data was visualized using the Interactive View tool of the Lung Cell Atlas (https://asthma.cellgeni.sanger.ac.uk/), and its associated, published, publicly available dataset (Vieira Braga et al., 2019).

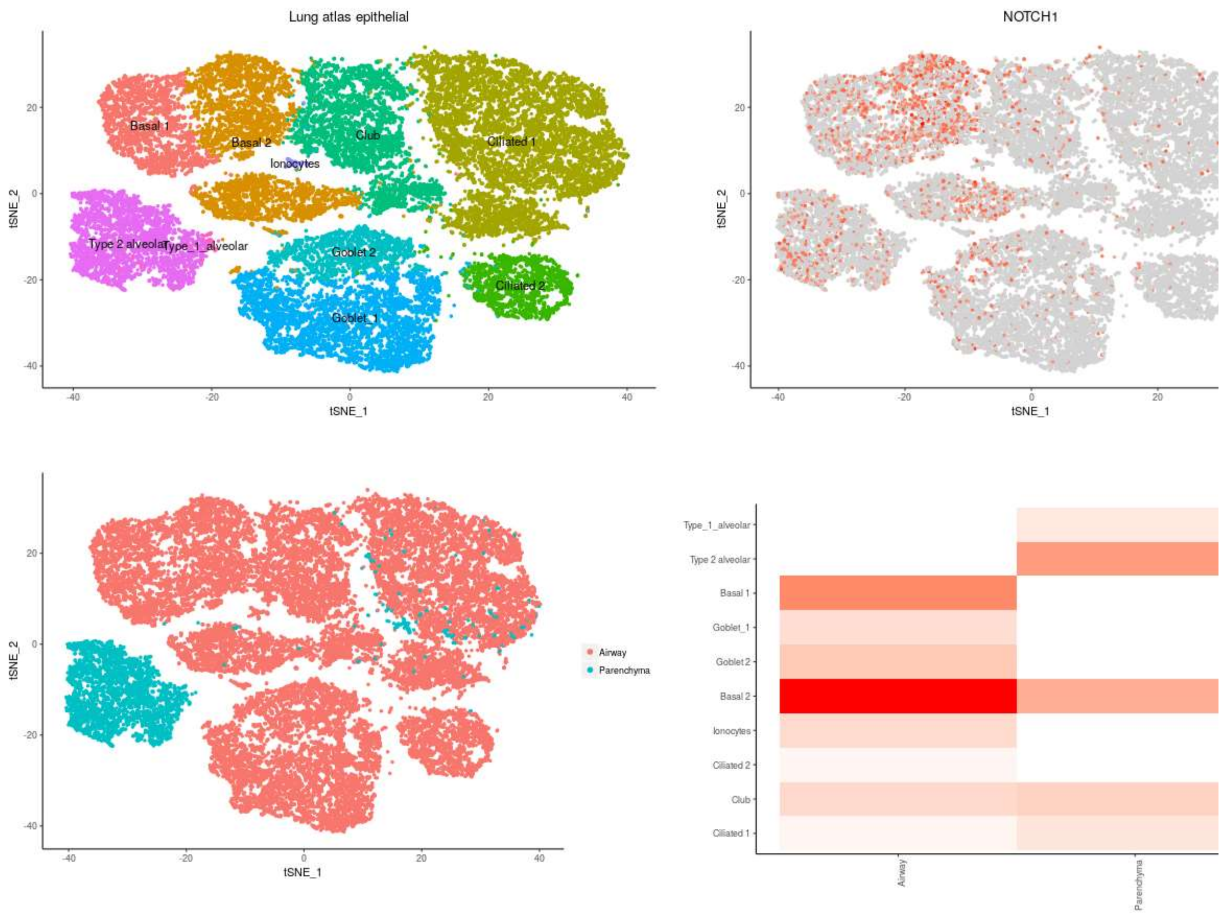

Figure S1a. NOTCH1, lung epithelium.

Upper left: Displaying unbiased clustering of different cell types in lung tissue; Upper right: t-SNE plot showing the expression of NOTCH1 in different cluster of cells; Lower left: Different cell types in upper airway and parenchymal lung tissue; Lower right: t-SNE plot showing the expression of NOTCH1 in upper airway and parenchymal lung tissue.

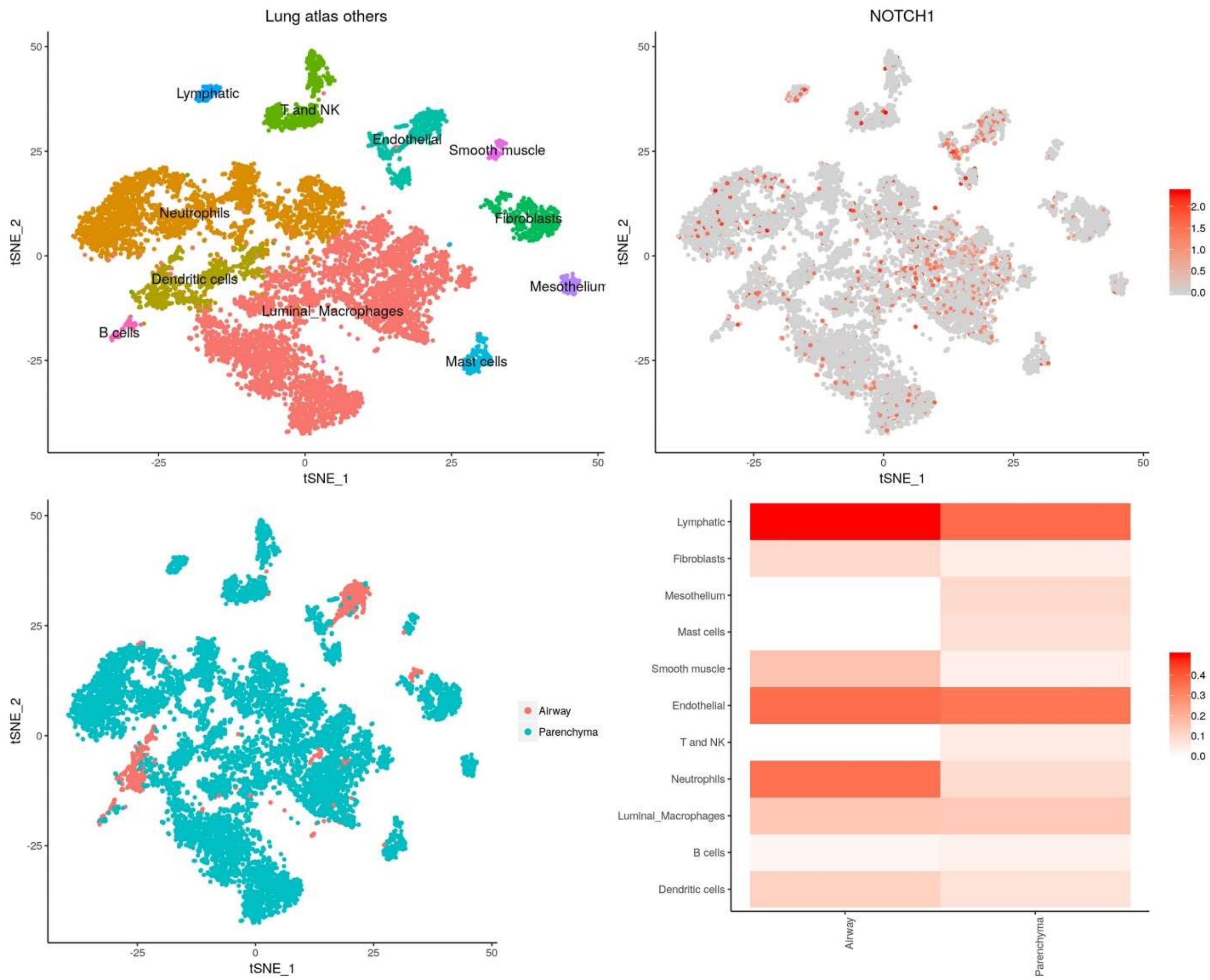

Figure S1b. NOTCH1, non-epithelium.

Upper left: Displaying unbiased clustering of different non-epithelial cell types in lung tissue; Upper right: t-SNE plot showing the expression of NOTCH1 in different cluster of cells; Lower left: Different cell types in upper airway and parenchymal lung tissue; Lower right: t-SNE plot showing the expression of NOTCH1 in upper airway and parenchymal lung tissue.

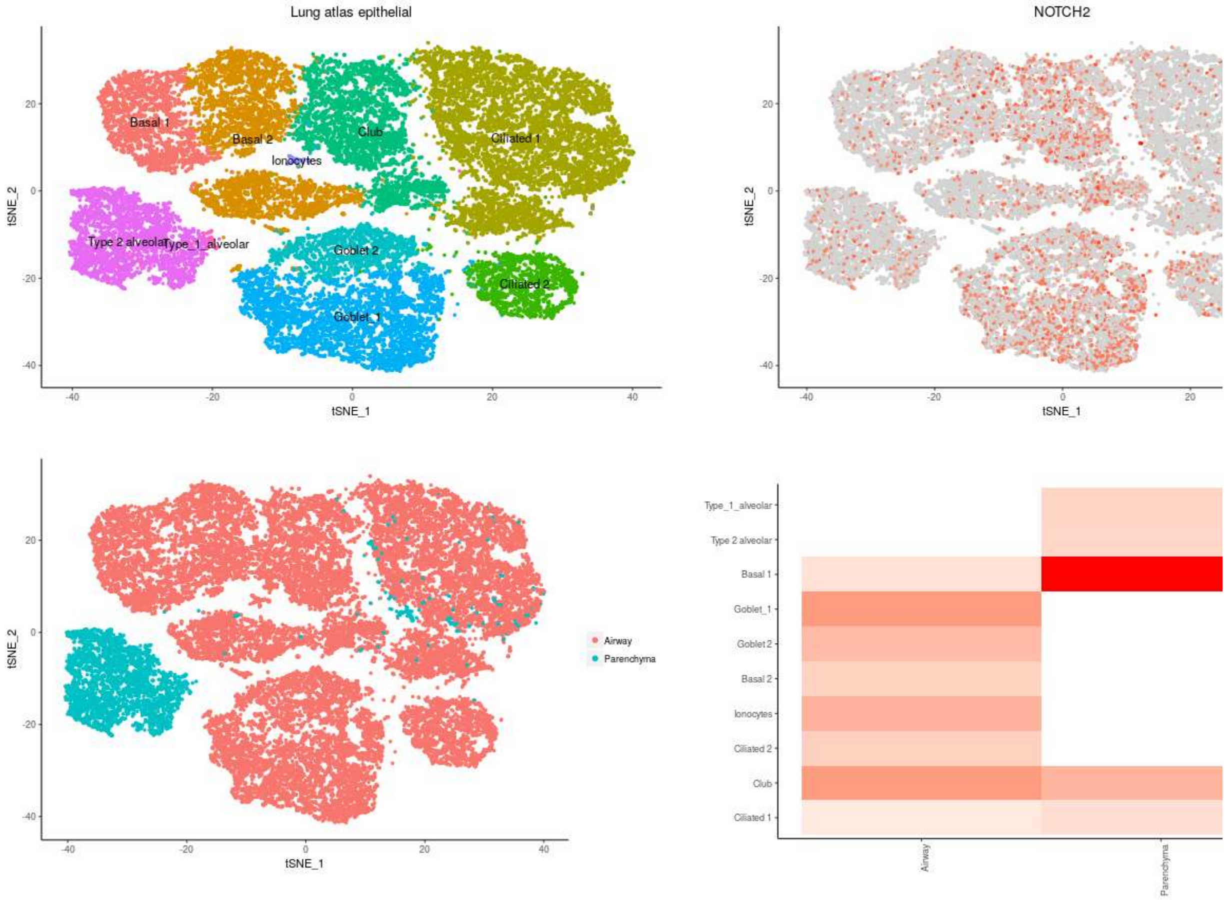

Figure S2a. NOTCH2, lung epithelium.

Upper left: Displaying unbiased clustering of different cell types in lung tissue; Upper right: t-SNE plot showing the expression of NOTCH2 in different cluster of cells; Lower left: Different cell types in upper airway and parenchymal lung tissue; Lower right: t-SNE plot showing the expression of NOTCH2 in upper airway and parenchymal lung tissue.

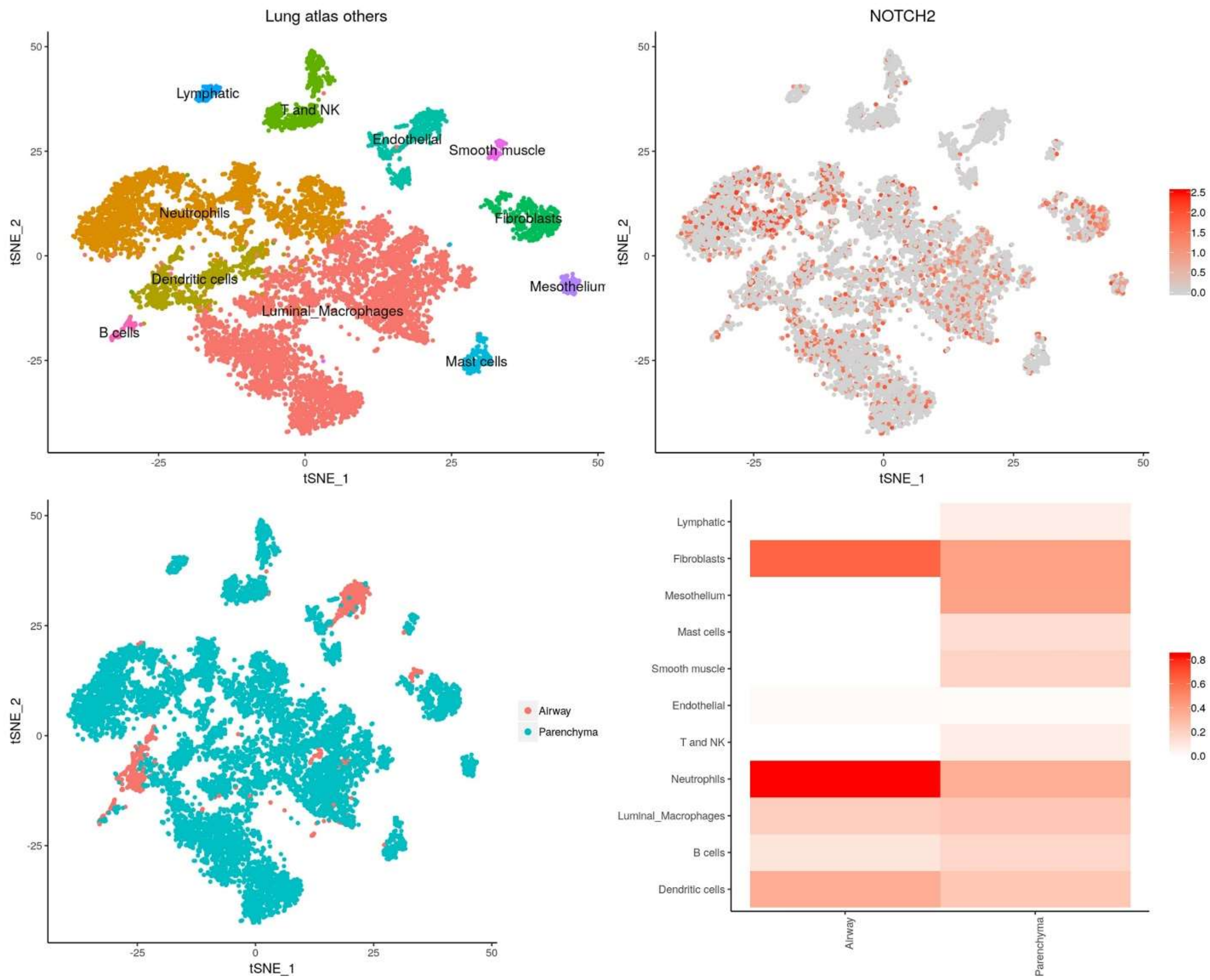

Figure S2b. NOTCH2, non-epithelium.

Upper left: Displaying unbiased clustering of different non-epithelial cell types in lung tissue; Upper right: t-SNE plot showing the expression of NOTCH2 in different cluster of cells; Lower left: Different cell types in upper airway and parenchymal lung tissue; Lower right: t-SNE plot showing the expression of NOTCH2 in upper airway and parenchymal lung tissue.

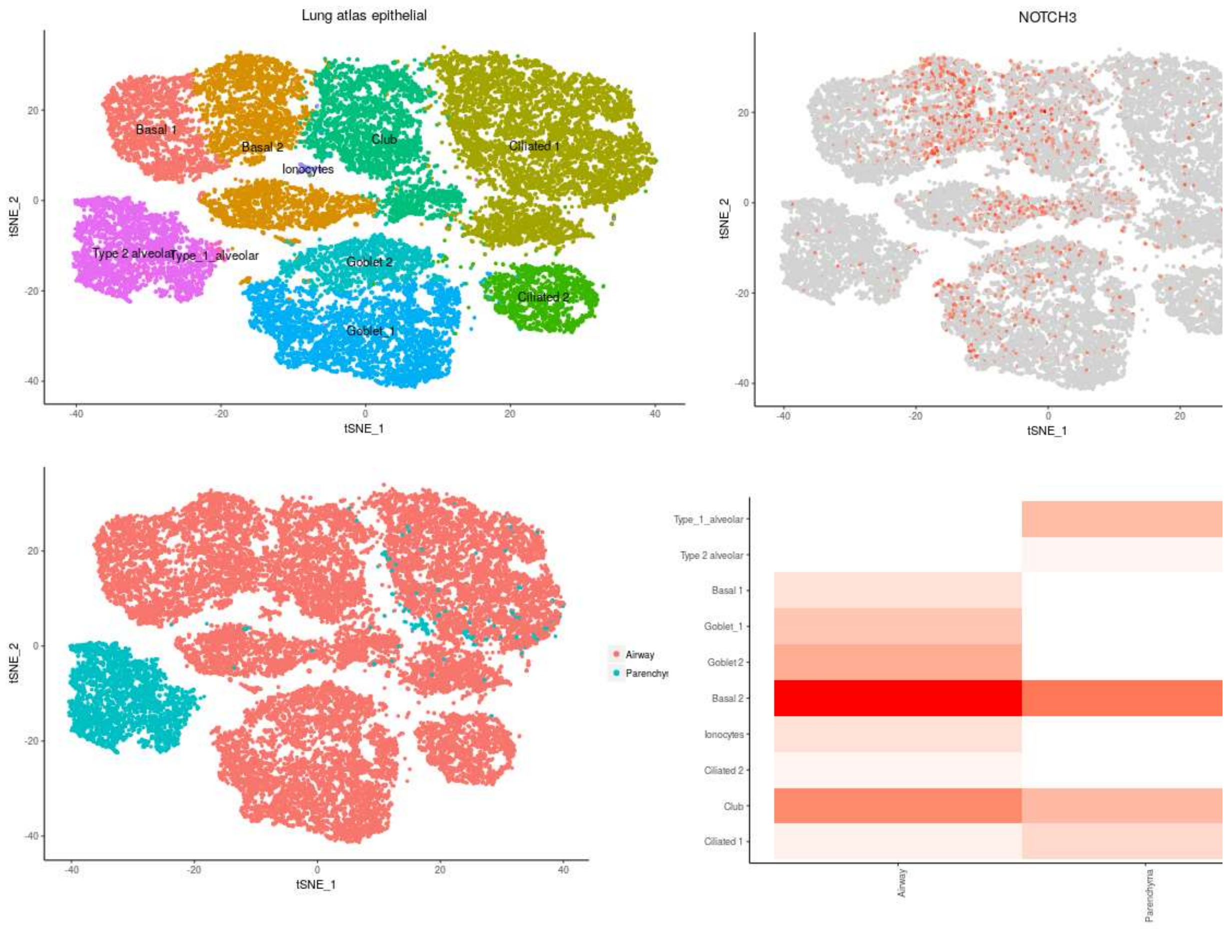

Figure S3a. NOTCH3, lung epithelium.

Upper left: Displaying unbiased clustering of different cell types in lung tissue; Upper right: t-SNE plot showing the expression of NOTCH3 in different cluster of cells; Lower left: Different cell types in upper airway and parenchymal lung tissue; Lower right: t-SNE plot showing the expression of NOTCH3 in upper airway and parenchymal lung tissue.

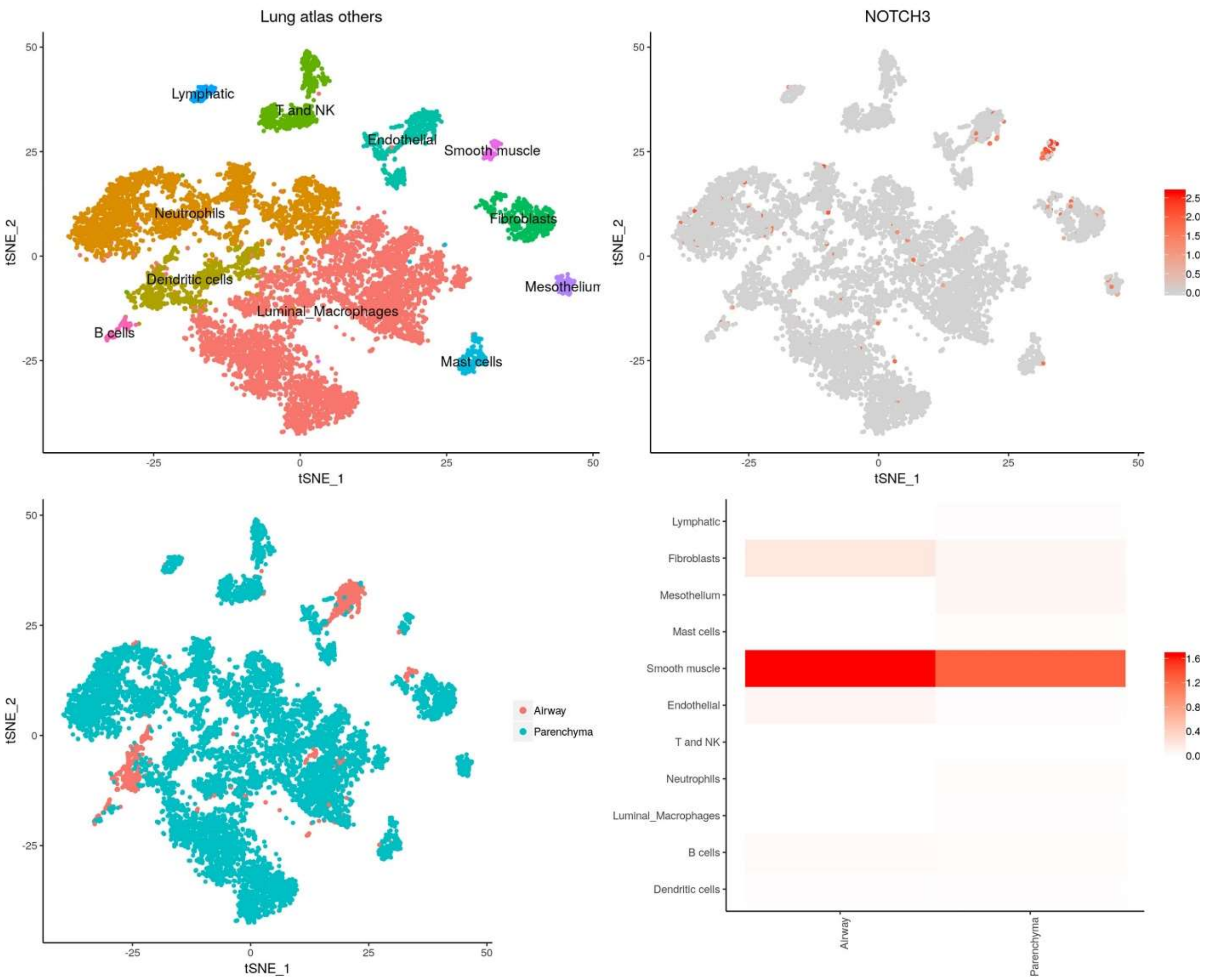

Figure S3b. NOTCH3, non-epithelium.

Upper left: Displaying unbiased clustering of different non-epithelial cell types in lung tissue; Upper right: t-SNE plot showing the expression of NOTCH3 in different cluster of cells; Lower left: Different cell types in upper airway and parenchymal lung tissue; Lower right: t-SNE plot showing the expression of NOTCH3 in upper airway and parenchymal lung tissue.

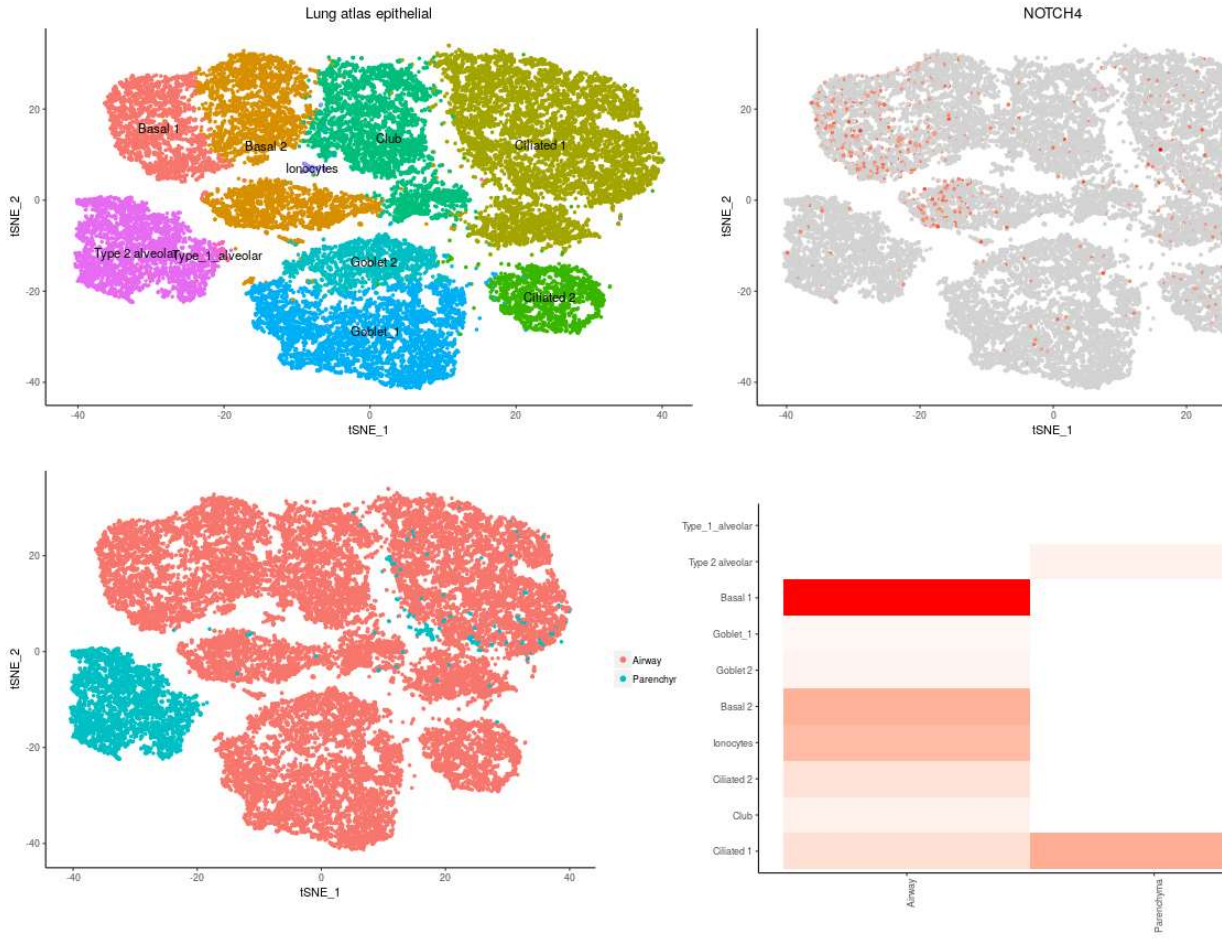

Figure S4a. NOTCH4, lung epithelium.

Upper left: Displaying unbiased clustering of different cell types in lung tissue; Upper right: t-SNE plot showing the expression of NOTCH4 in different cluster of cells; Lower left: Different cell types in upper airway and parenchymal lung tissue; Lower right: t-SNE plot showing the expression of NOTCH4 in upper airway and parenchymal lung tissue.

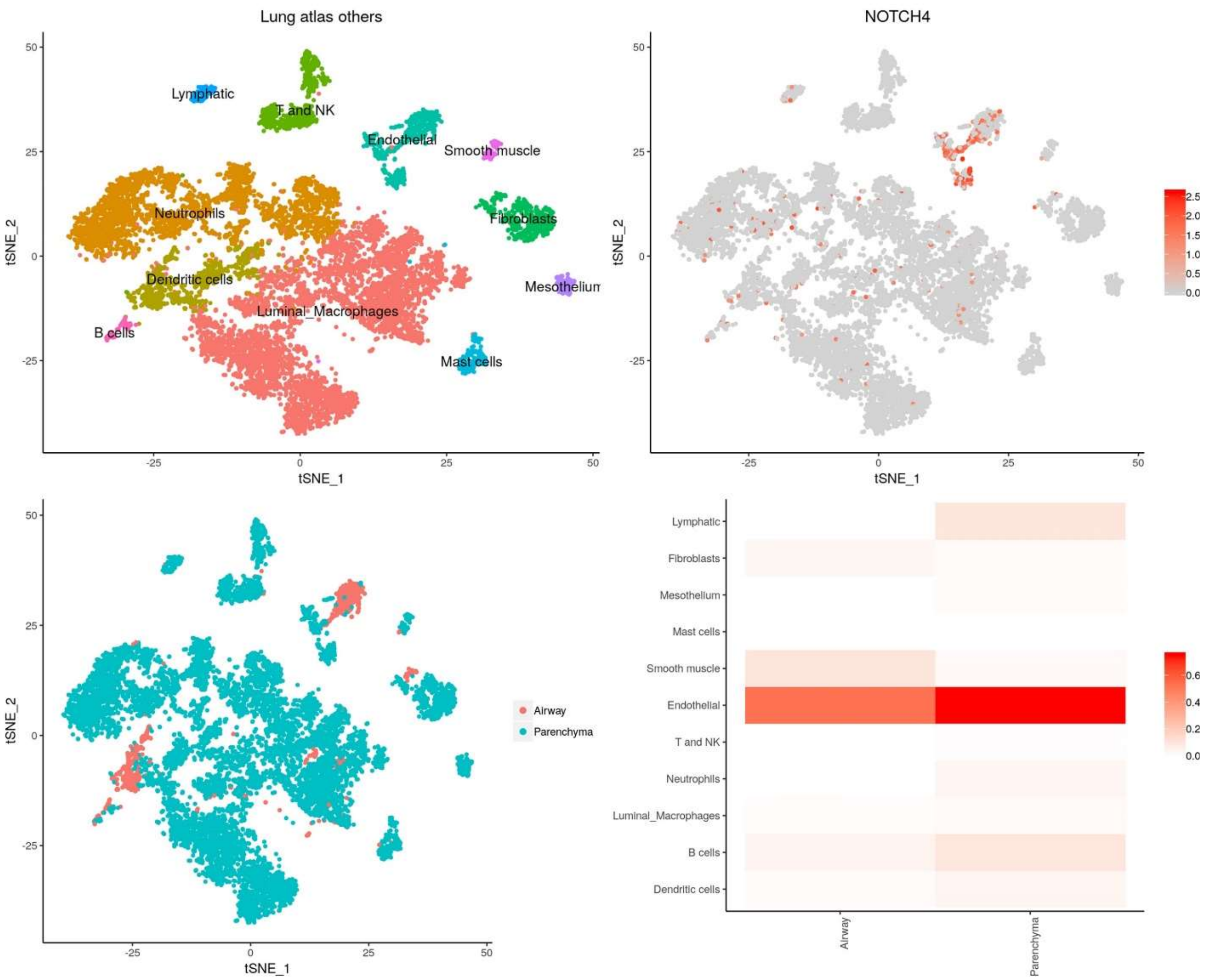

Figure S4b. NOTCH4, non-epithelium.

Upper left: Displaying unbiased clustering of different non-epithelial cell types in lung tissue; Upper right: t-SNE plot showing the expression of NOTCH4 in different cluster of cells; Lower left: Different cell types in upper airway and parenchymal lung tissue; Lower right: t-SNE plot showing the expression of NOTCH4 in upper airway and parenchymal lung tissue.

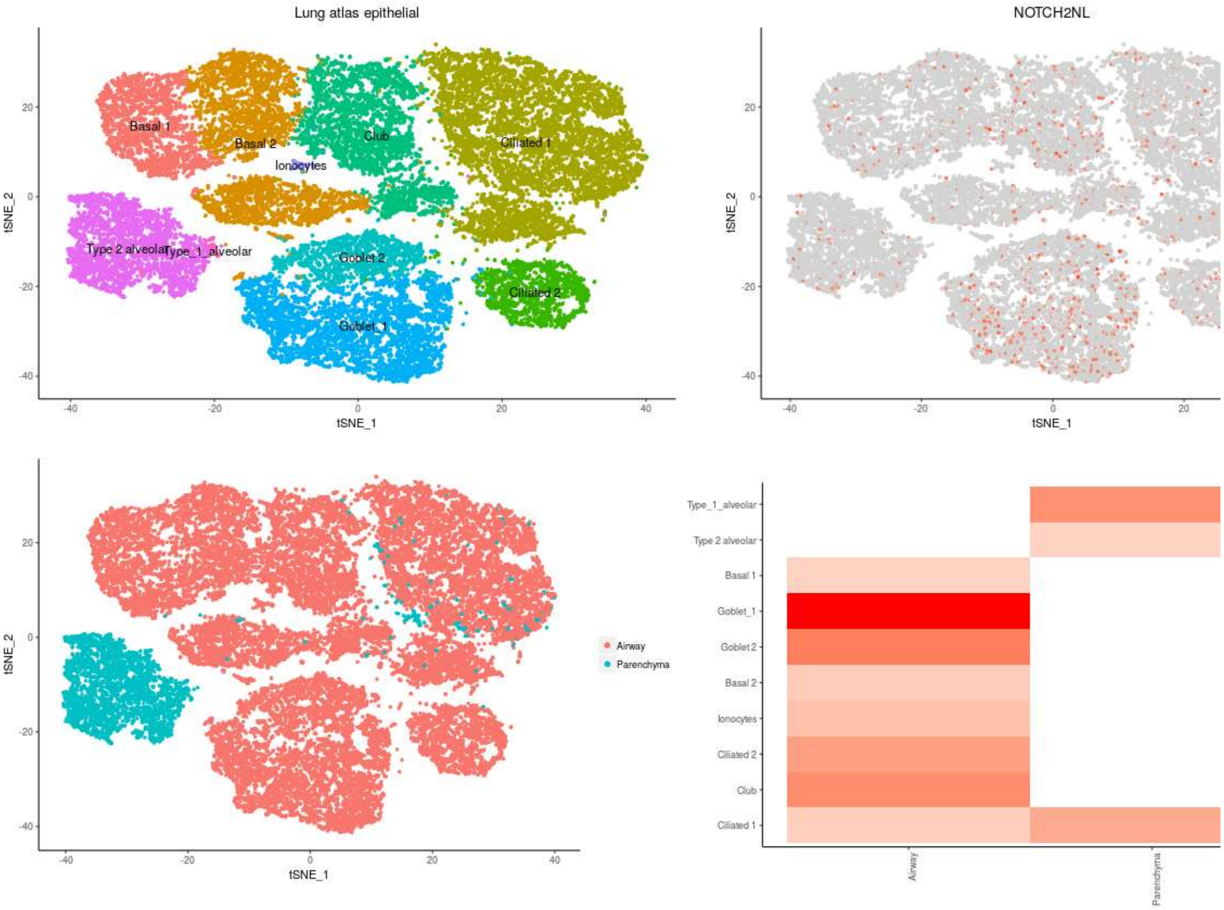

Figure S5a. NOTCH2NL, lung epithelium.

Upper left: Displaying unbiased clustering of different cell types in lung tissue; Upper right: t-SNE plot showing the expression of NOTCH2NL in different cluster of cells; Lower left: Different cell types in upper airway and parenchymal lung tissue; Lower right: t-SNE plot showing the expression of NOTCH2NL in upper airway and parenchymal lung tissue.

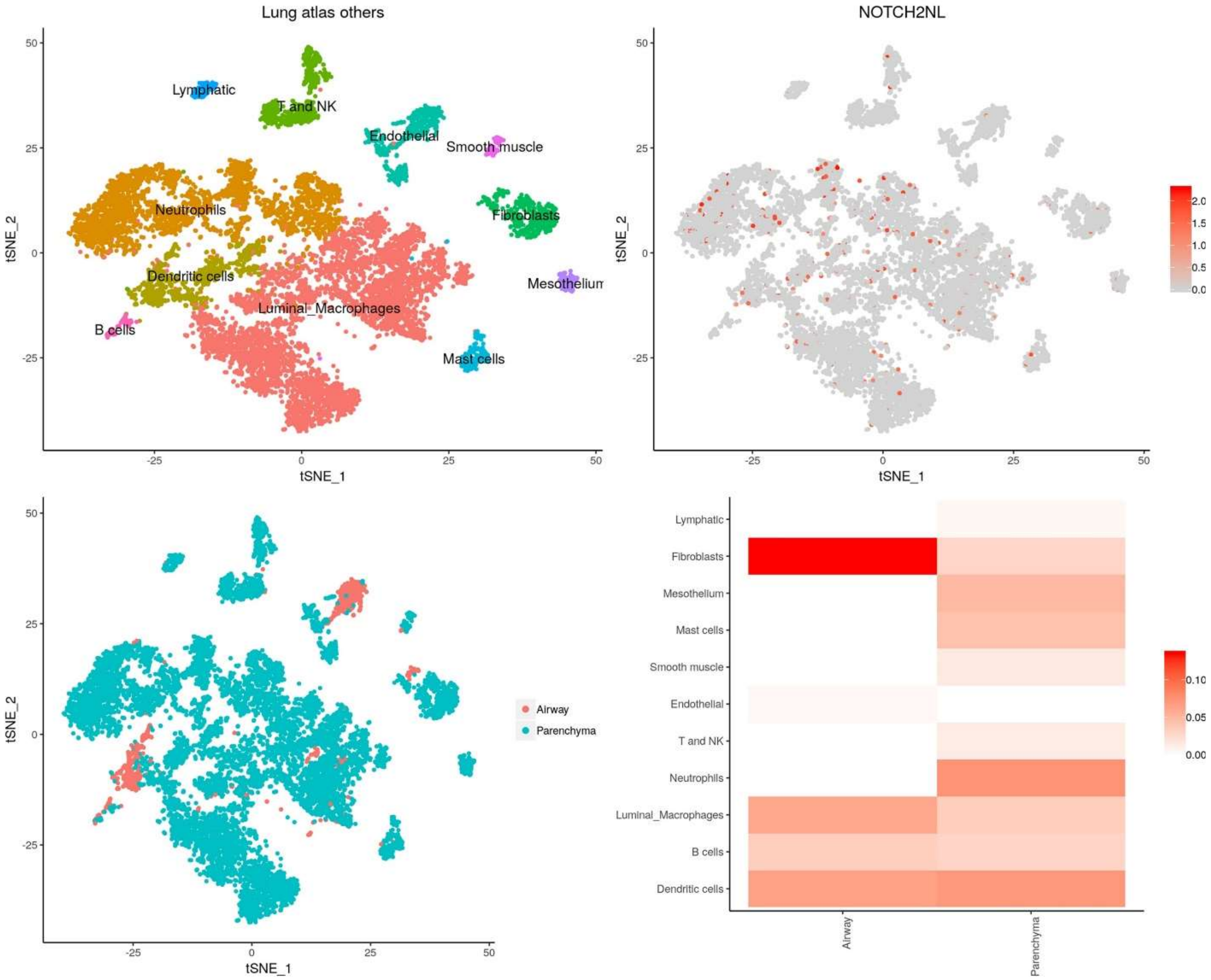

Figure S5b. NOTCH2NL, non-epithelium.

Upper left: Displaying unbiased clustering of different non-epithelial cell types in lung tissue; Upper right: t-SNE plot showing the expression of NOTCH2NL in different cluster of cells; Lower left: Different cell types in upper airway and parenchymal lung tissue; Lower right: t-SNE plot showing the expression of NOTCH2NL in upper airway and parenchymal lung tissue.

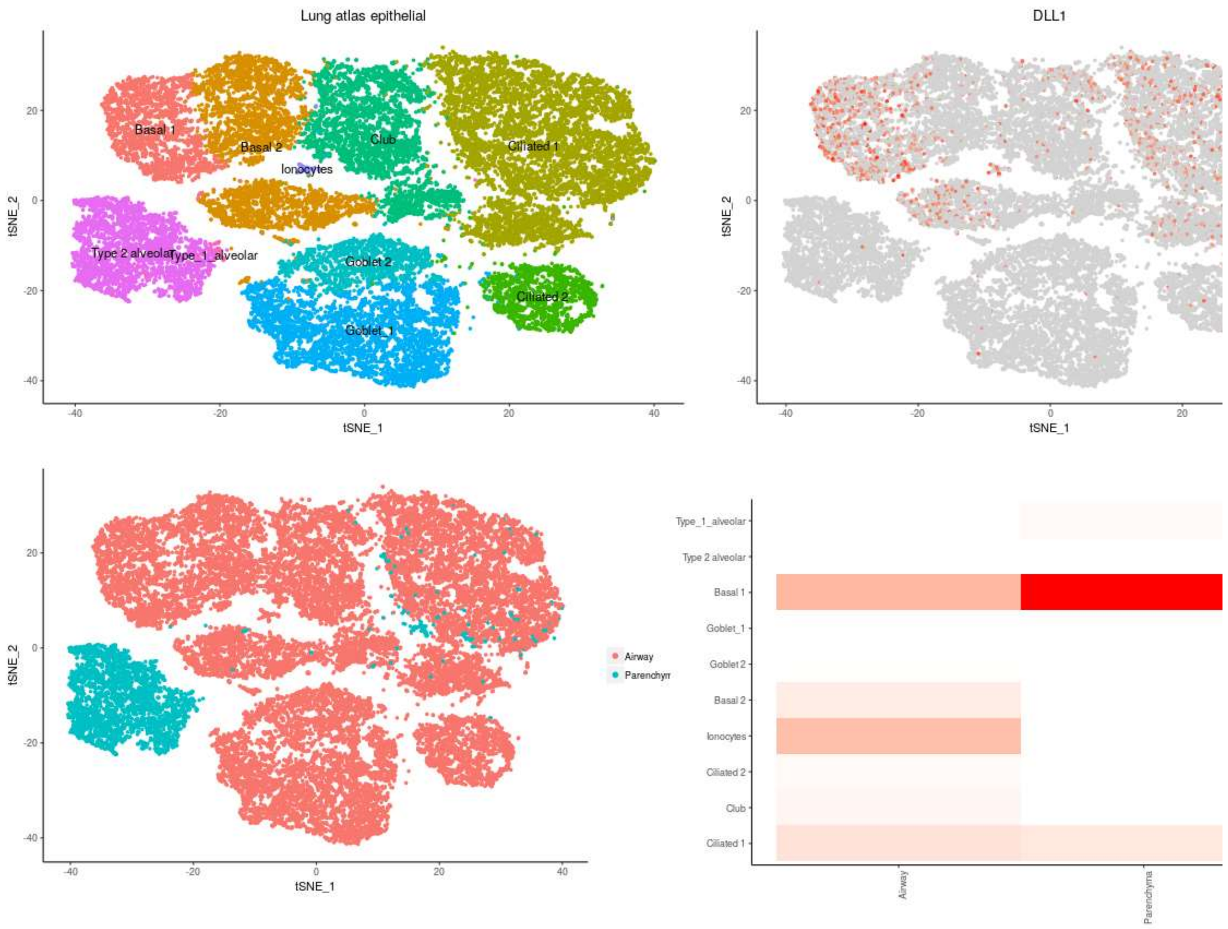

Figure S6a. DLL1, lung epithelium.

Upper left: Displaying unbiased clustering of different cell types in lung tissue; Upper right: t-SNE plot showing the expression of DLL1 in different cluster of cells; Lower left: Different cell types in upper airway and parenchymal lung tissue; Lower right: t-SNE plot showing the expression of DLL1 in upper airway and parenchymal lung tissue.

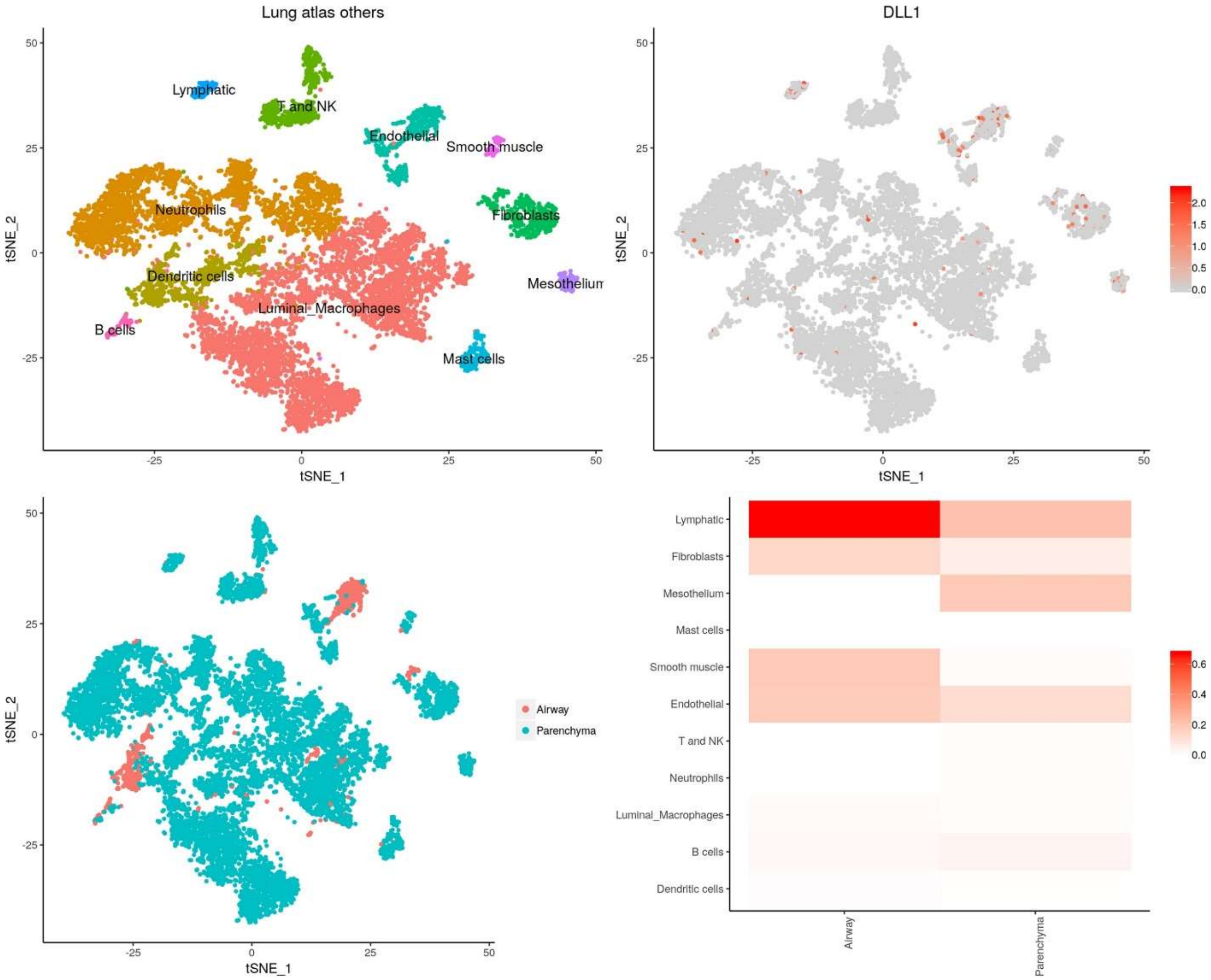

Figure S6b. DLL1, non-epithelium.

Upper left: Displaying unbiased clustering of different non-epithelial cell types in lung tissue; Upper right: t-SNE plot showing the expression of DLL1 in different cluster of cells; Lower left: Different cell types in upper airway and parenchymal lung tissue; Lower right: t-SNE plot showing the expression of DLL1 in upper airway and parenchymal lung tissue.

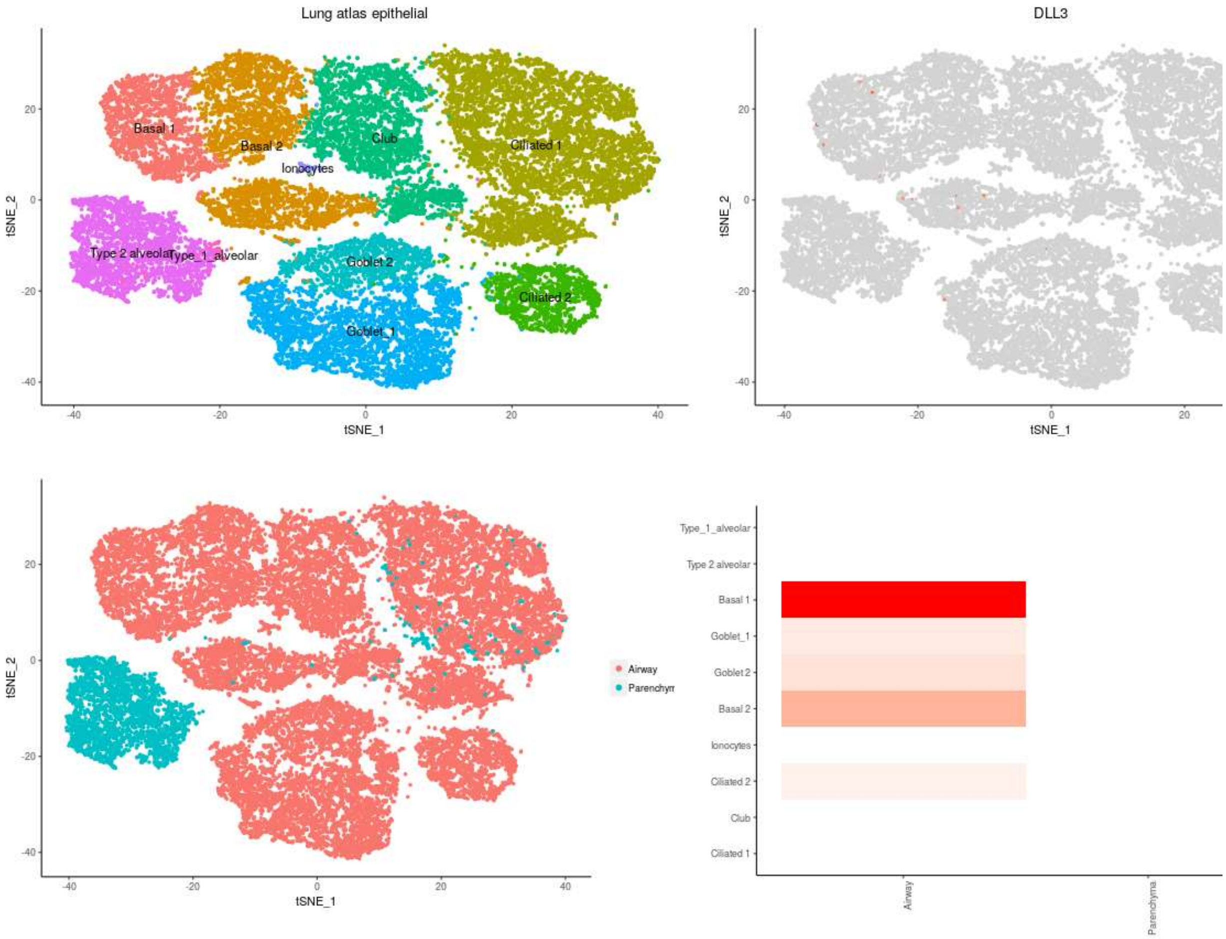

Figure S7a. DLL3, lung epithelium.

Upper left: Displaying unbiased clustering of different cell types in lung tissue; Upper right: t-SNE plot showing the expression of DLL3 in different cluster of cells; Lower left: Different cell types in upper airway and parenchymal lung tissue; Lower right: t-SNE plot showing the expression of DLL3 in upper airway and parenchymal lung tissue.

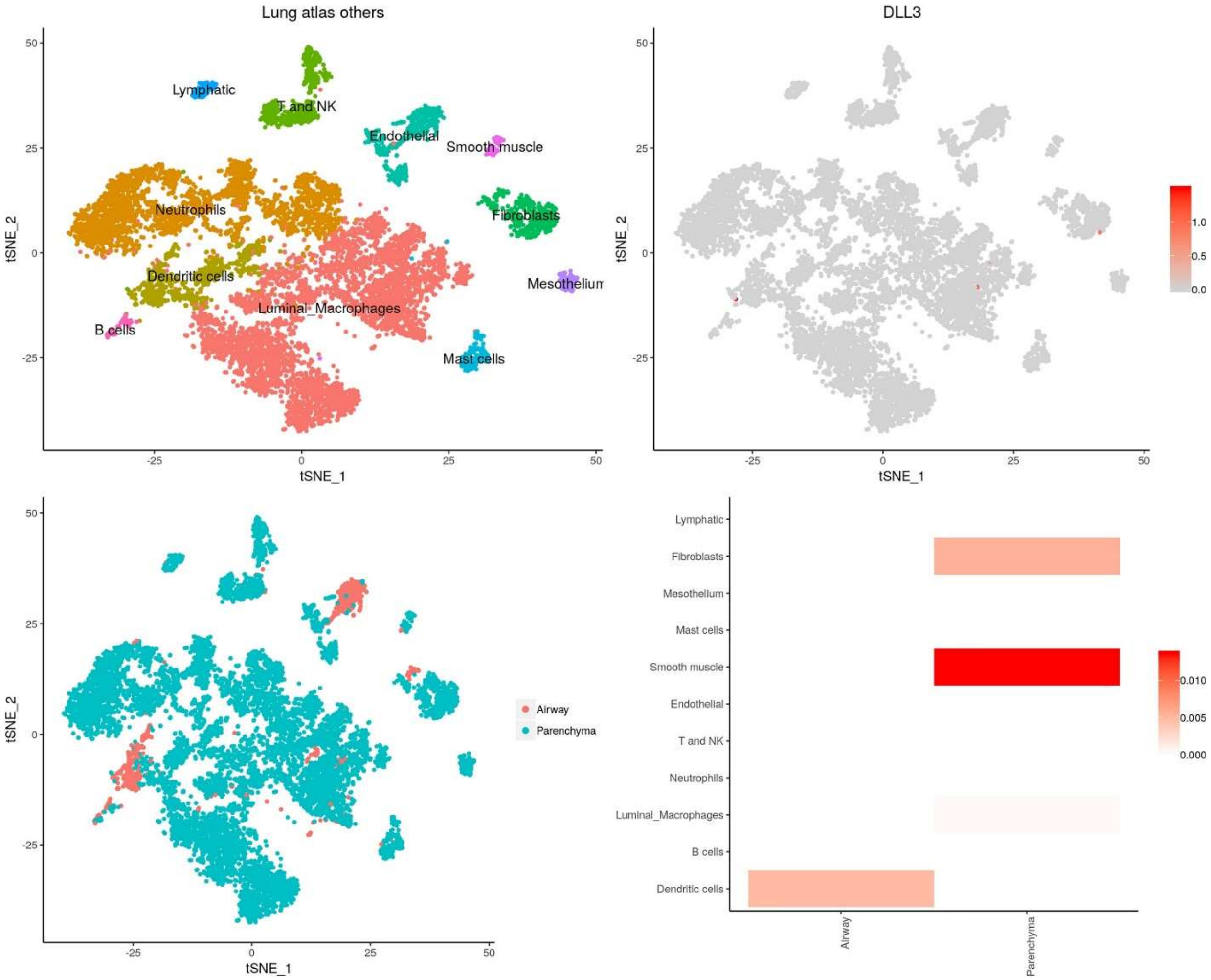

Figure S7b. DLL3, non-epithelium.

Upper left: Displaying unbiased clustering of different non-epithelial cell types in lung tissue; Upper right: t-SNE plot showing the expression of DLL3 in different cluster of cells; Lower left: Different cell types in upper airway and parenchymal lung tissue; Lower right: t-SNE plot showing the expression of DLL3 in upper airway and parenchymal lung tissue.

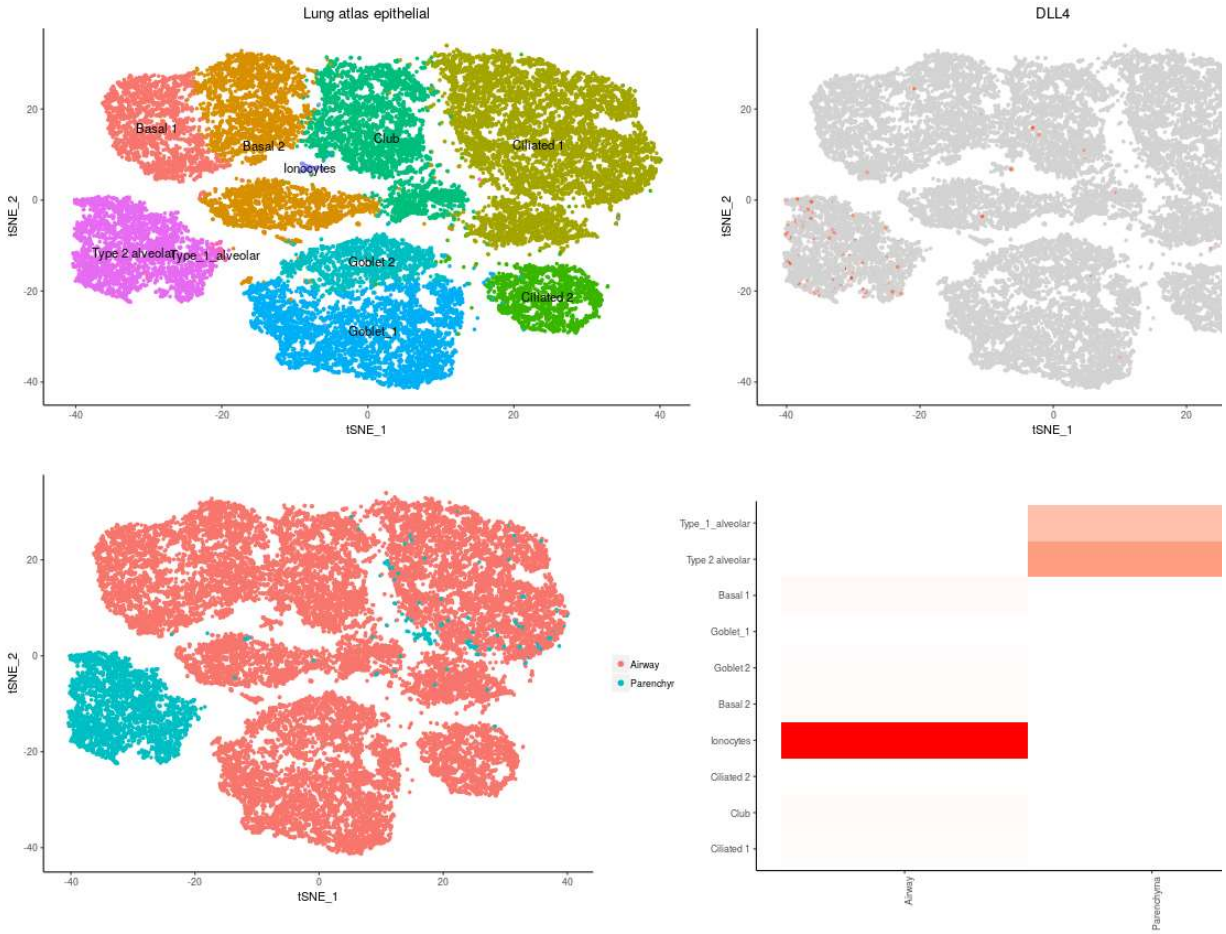

Figure S8a. DLL4, lung epithelium.

Upper left: Displaying unbiased clustering of different cell types in lung tissue; Upper right: t-SNE plot showing the expression of DLL4 in different cluster of cells; Lower left: Different cell types in upper airway and parenchymal lung tissue; Lower right: t-SNE plot showing the expression of DLL4 in upper airway and parenchymal lung tissue.

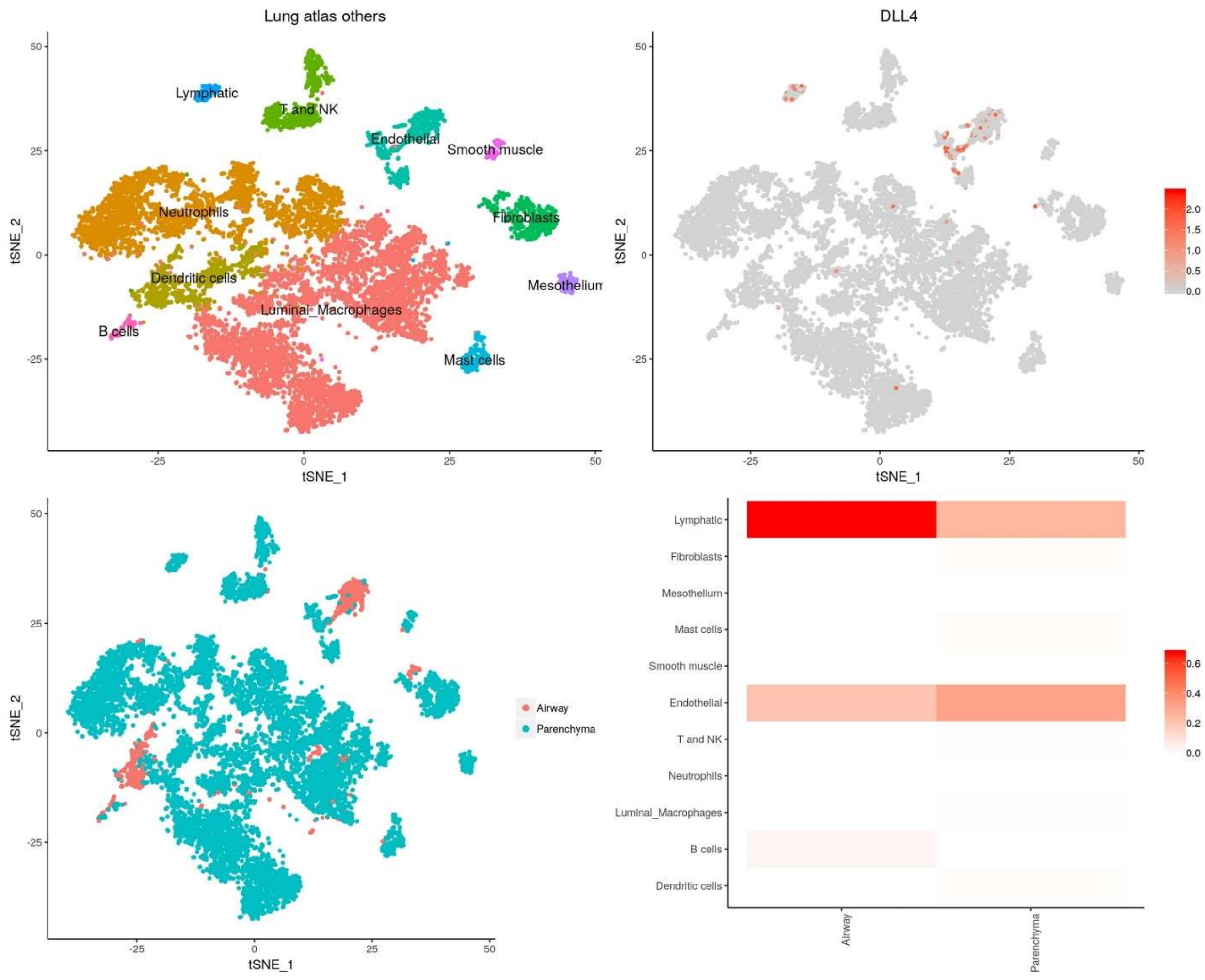

Figure S8b. DLL4, non-epithelium.

Upper left: Displaying unbiased clustering of different non-epithelial cell types in lung tissue; Upper right: t-SNE plot showing the expression of DLL4 in different cluster of cells; Lower left: Different cell types in upper airway and parenchymal lung tissue; Lower right: t-SNE plot showing the expression of DLL4 in upper airway and parenchymal lung tissue.

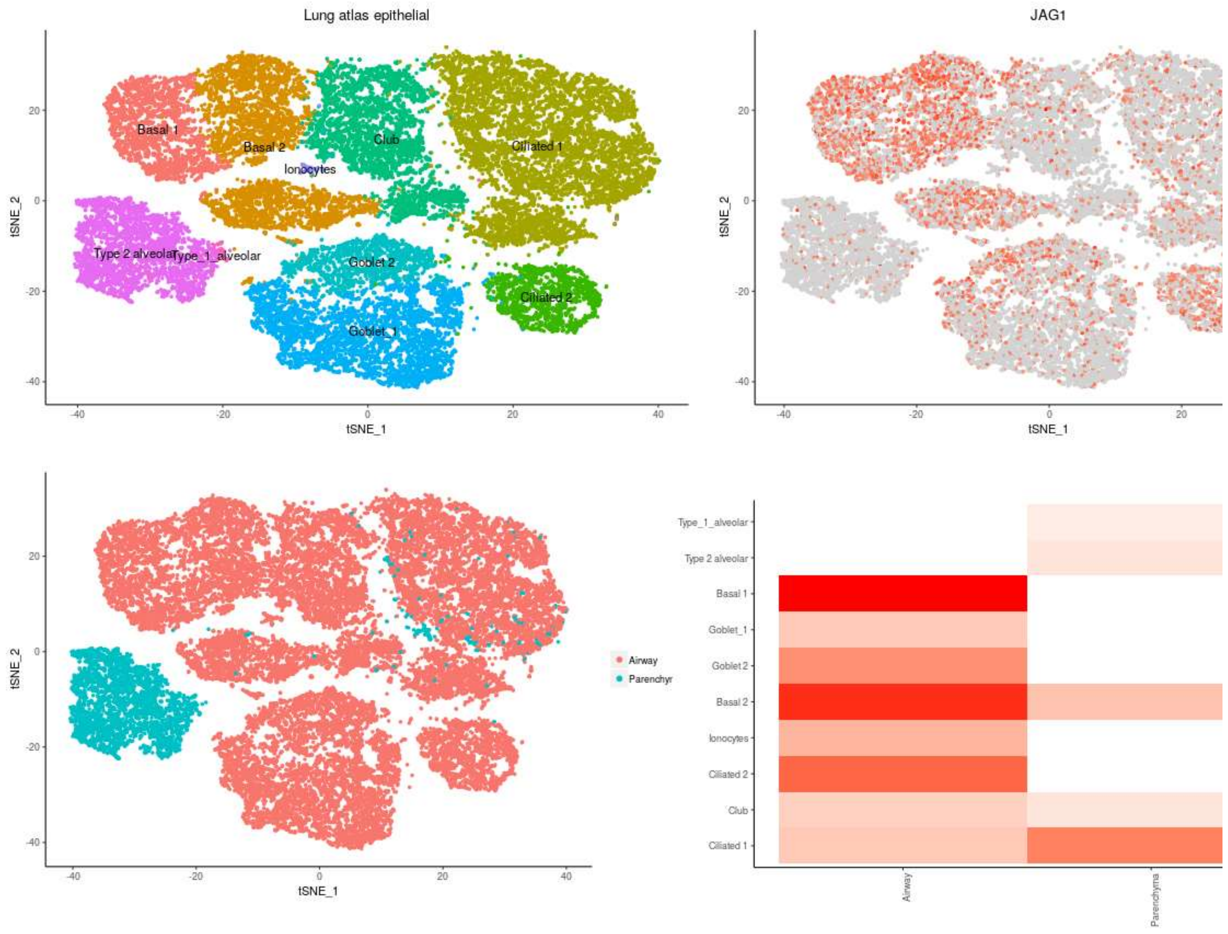

Figure S9a. JAG1, lung epithelium.

Upper left: Displaying unbiased clustering of different cell types in lung tissue; Upper right: t-SNE plot showing the expression of JAG1 in different cluster of cells; Lower left: Different cell types in upper airway and parenchymal lung tissue; Lower right: t-SNE plot showing the expression of JAG1 in upper airway and parenchymal lung tissue.

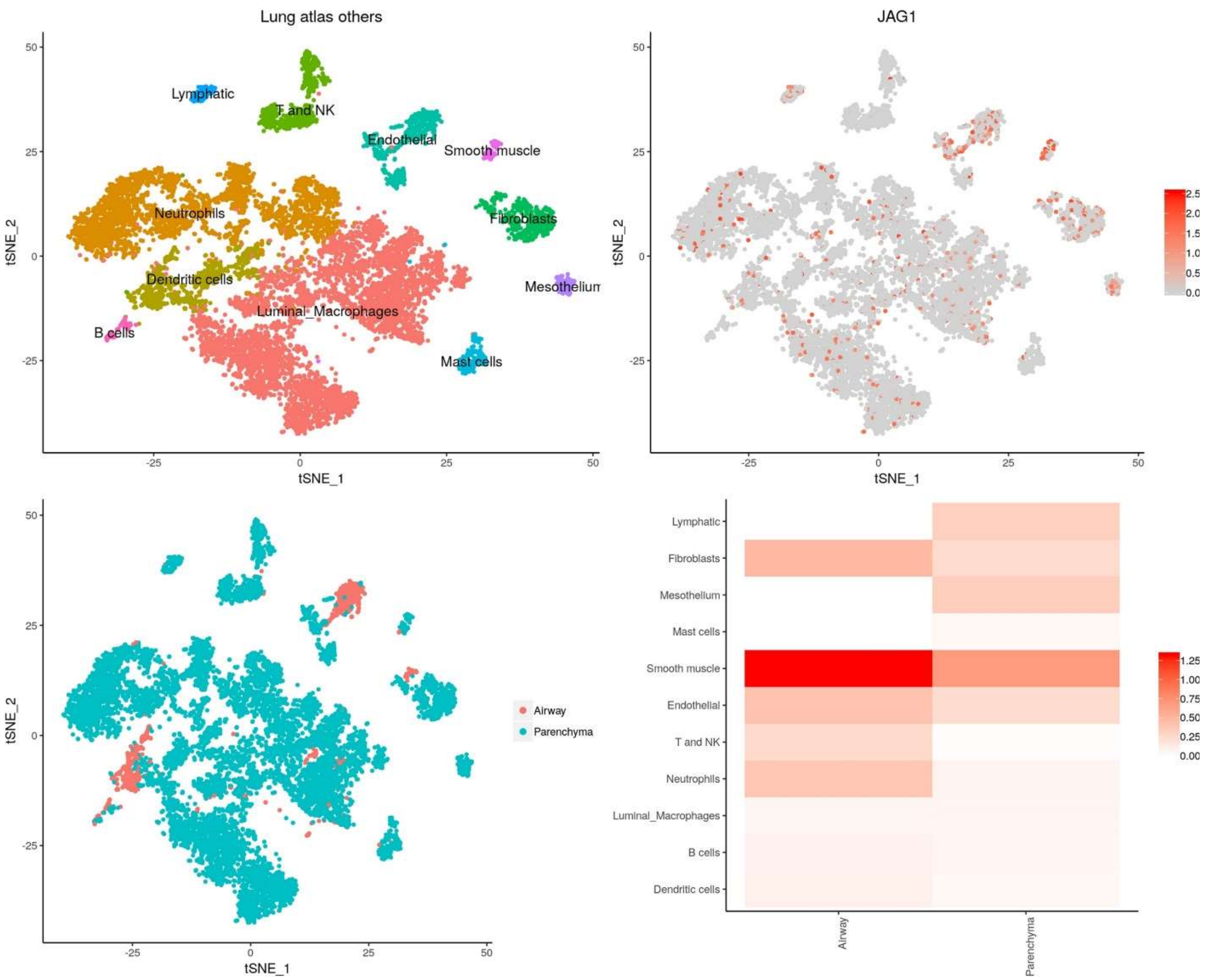

Figure S9b. JAG1, non-epithelium.

Upper left: Displaying unbiased clustering of different non-epithelial cell types in lung tissue; Upper right: t-SNE plot showing the expression of JAG1 in different cluster of cells; Lower left: Different cell types in upper airway and parenchymal lung tissue; Lower right: t-SNE plot showing the expression of JAG1 in upper airway and parenchymal lung tissue.

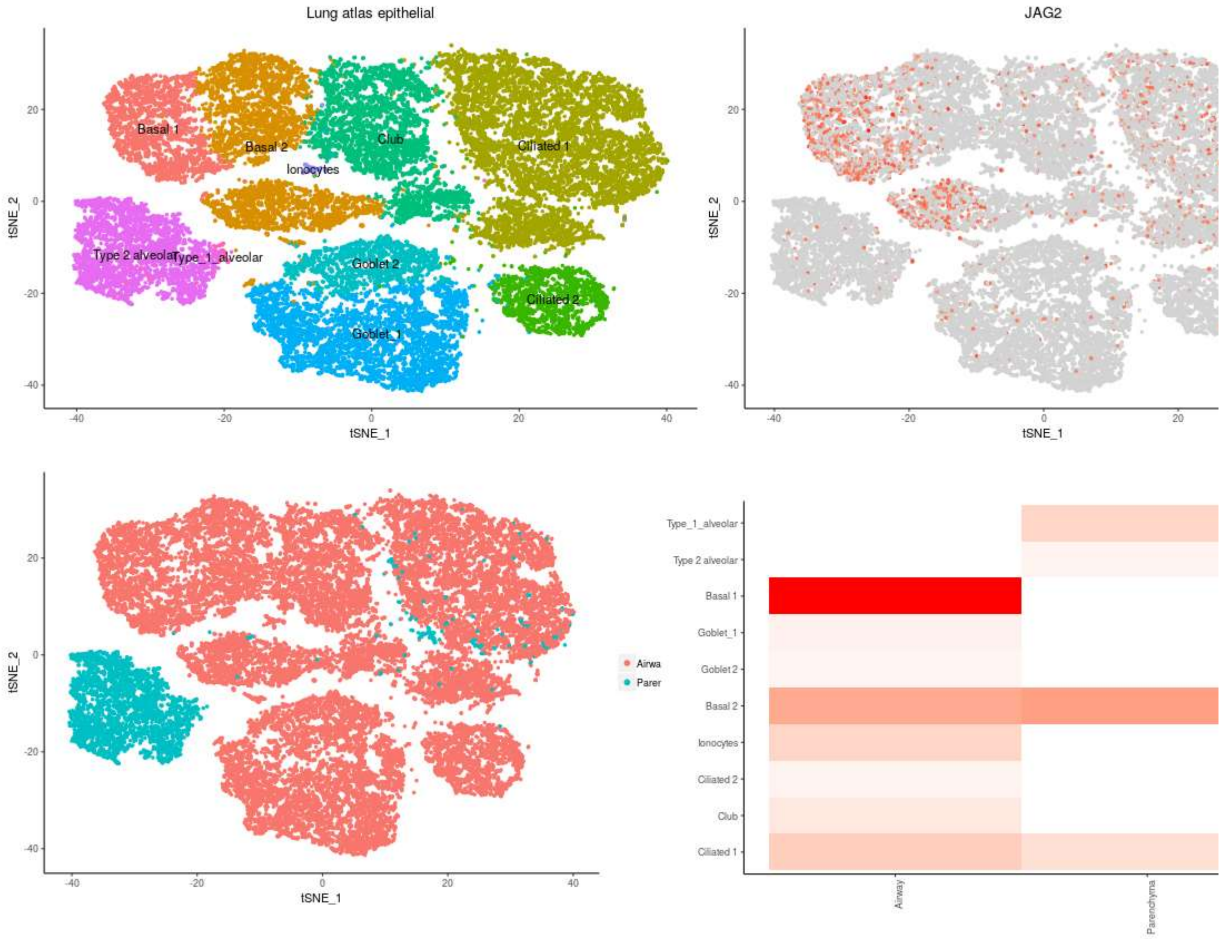

Figure S10a. JAG2, lung epithelium.

Upper left: Displaying unbiased clustering of different cell types in lung tissue; Upper right: t-SNE plot showing the expression of JAG2 in different cluster of cells; Lower left: Different cell types in upper airway and parenchymal lung tissue; Lower right: t-SNE plot showing the expression of JAG2 in upper airway and parenchymal lung tissue.

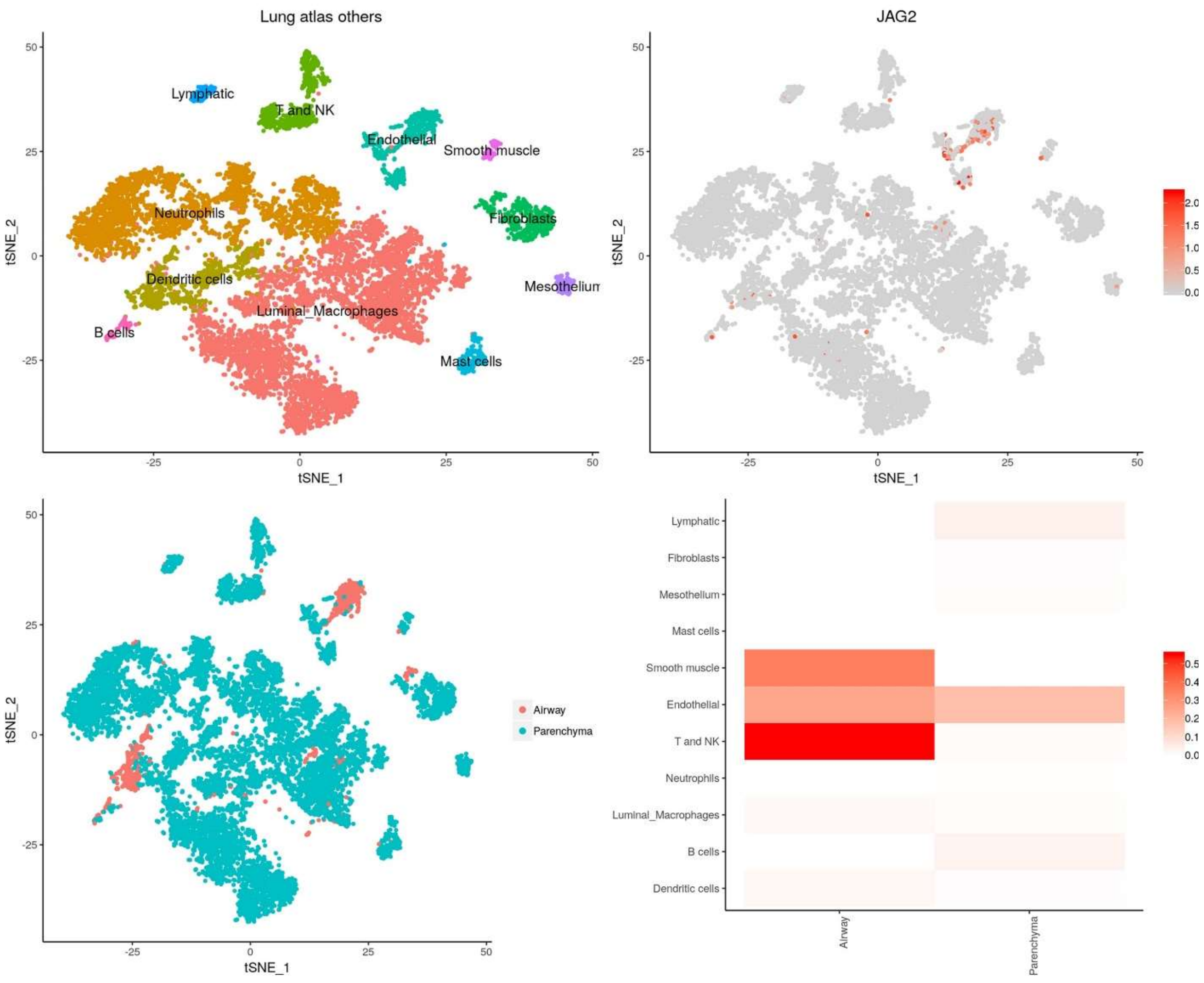

Figure S10b. JAG2, non-epithelium.

Upper left: Displaying unbiased clustering of different non-epithelial cell types in lung tissue; Upper right: t-SNE plot showing the expression of JAG2 in different cluster of cells; Lower left: Different cell types in upper airway and parenchymal lung tissue; Lower right: t-SNE plot showing the expression of JAG2 in upper airway and parenchymal lung tissue.

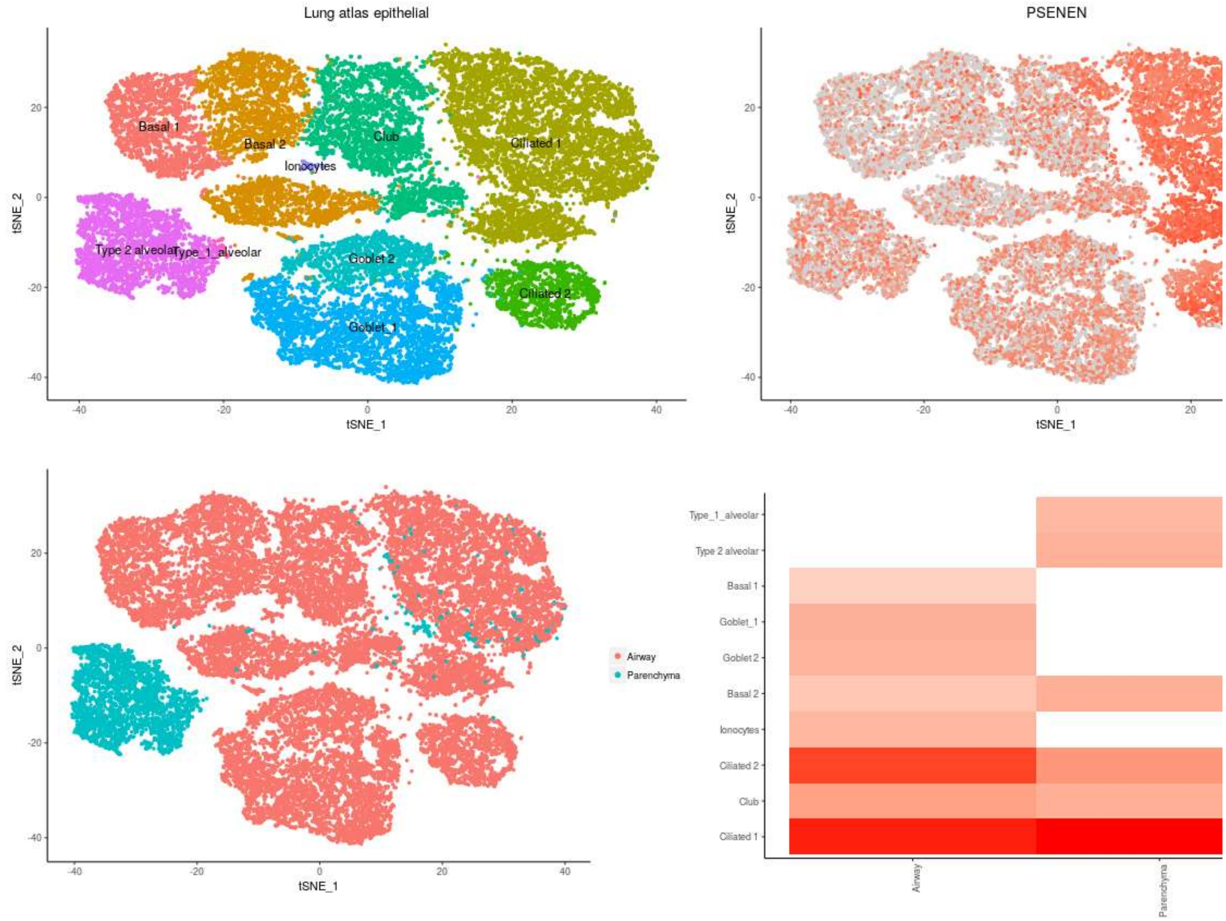

Figure S11a. PSENEN, lung epithelium.

Upper left: Displaying unbiased clustering of different cell types in lung tissue; Upper right: t-SNE plot showing the expression of PSENEN in different cluster of cells; Lower left: Different cell types in upper airway and parenchymal lung tissue; Lower right: t-SNE plot showing the expression of PSENEN in upper airway and parenchymal lung tissue.

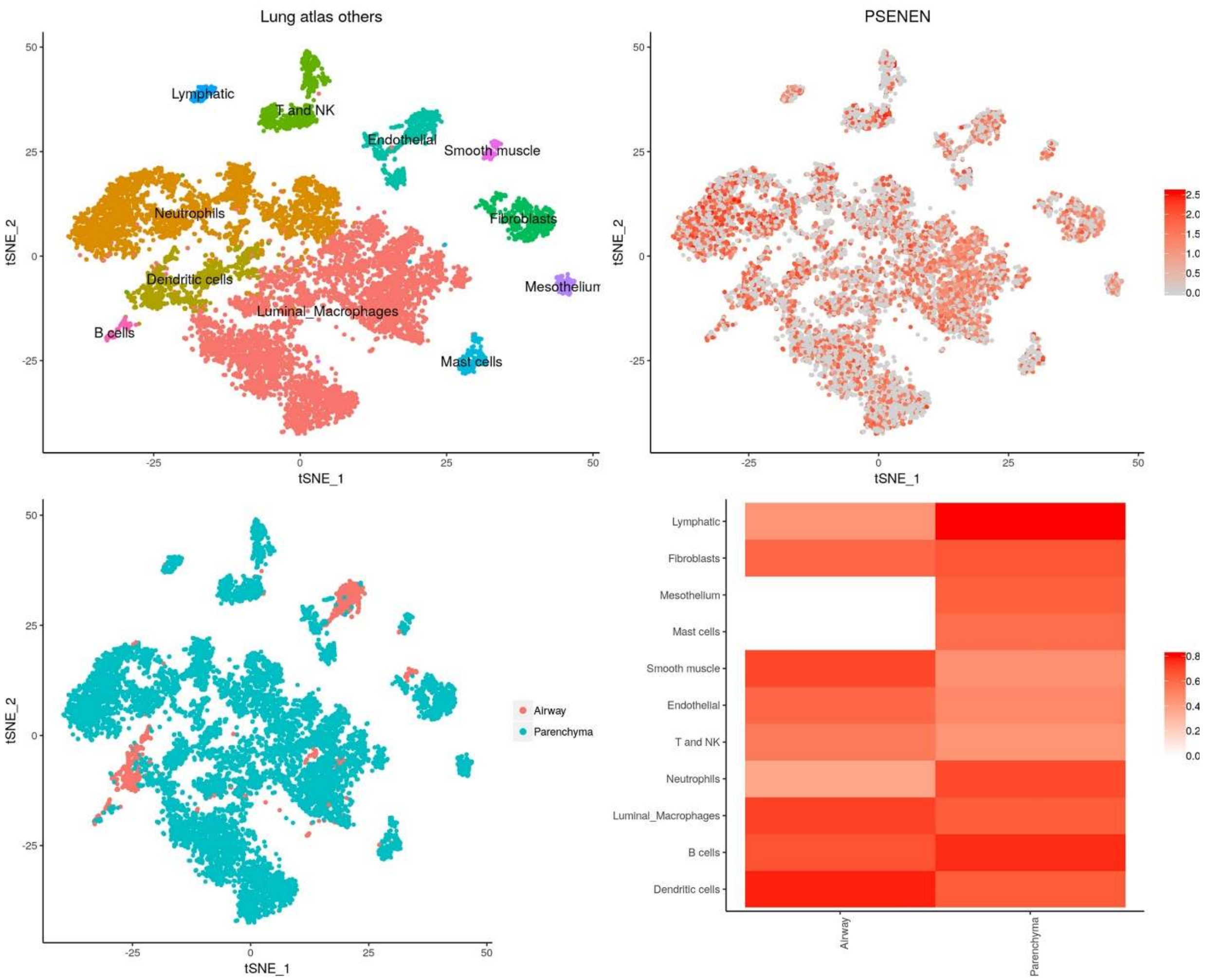

Figure S11b. PSENEN, non-epithelium.

Upper left: Displaying unbiased clustering of different non-epithelial cell types in lung tissue; Upper right: t-SNE plot showing the expression of PSENEN in different cluster of cells; Lower left: Different cell types in upper airway and parenchymal lung tissue; Lower right: t-SNE plot showing the expression of PSENEN in upper airway and parenchymal lung tissue.

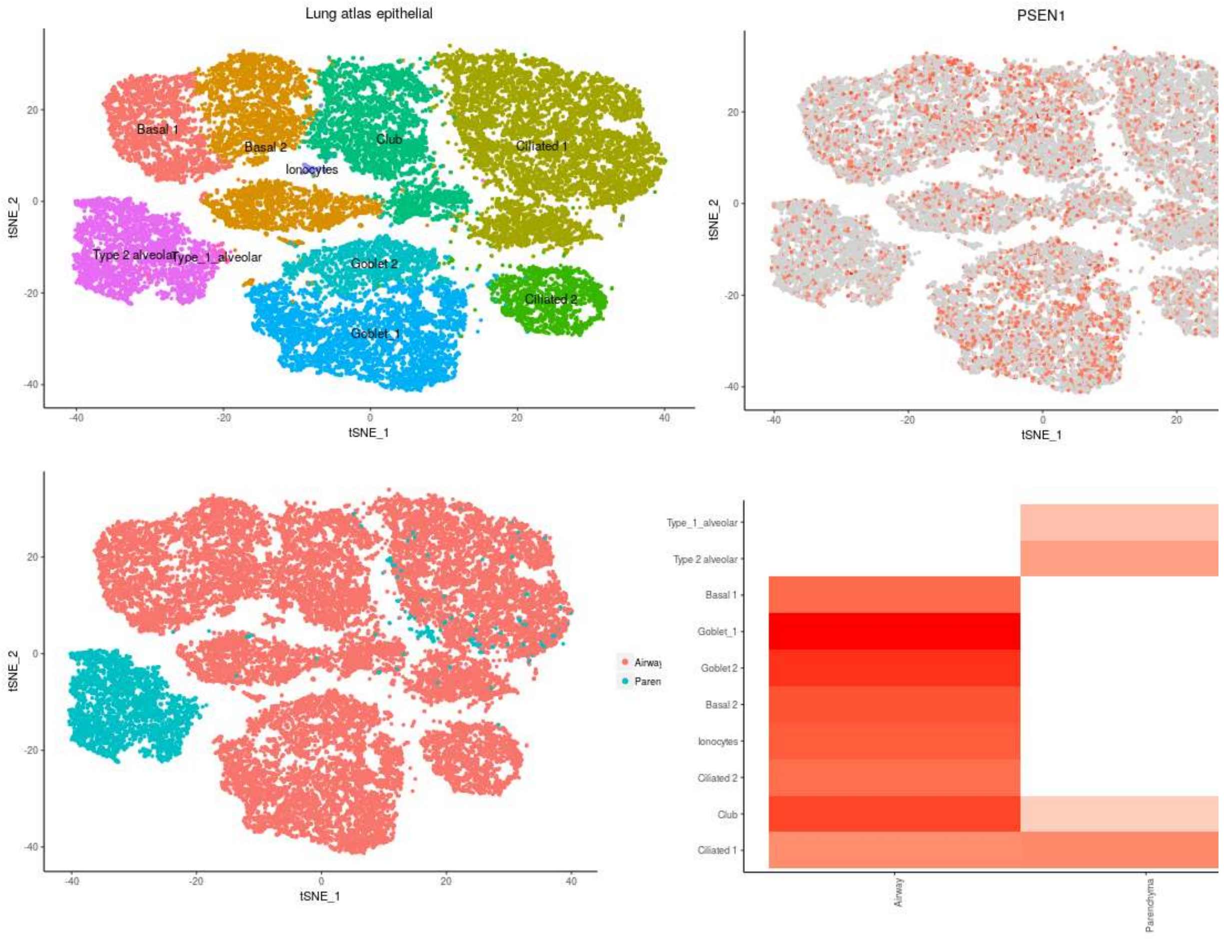

Figure S12a. PSEN1, lung epithelium.

Upper left: Displaying unbiased clustering of different cell types in lung tissue; Upper right: t-SNE plot showing the expression of PSEN1 in different cluster of cells; Lower left: Different cell types in upper airway and parenchymal lung tissue; Lower right: t-SNE plot showing the expression of PSEN1 in upper airway and parenchymal lung tissue.

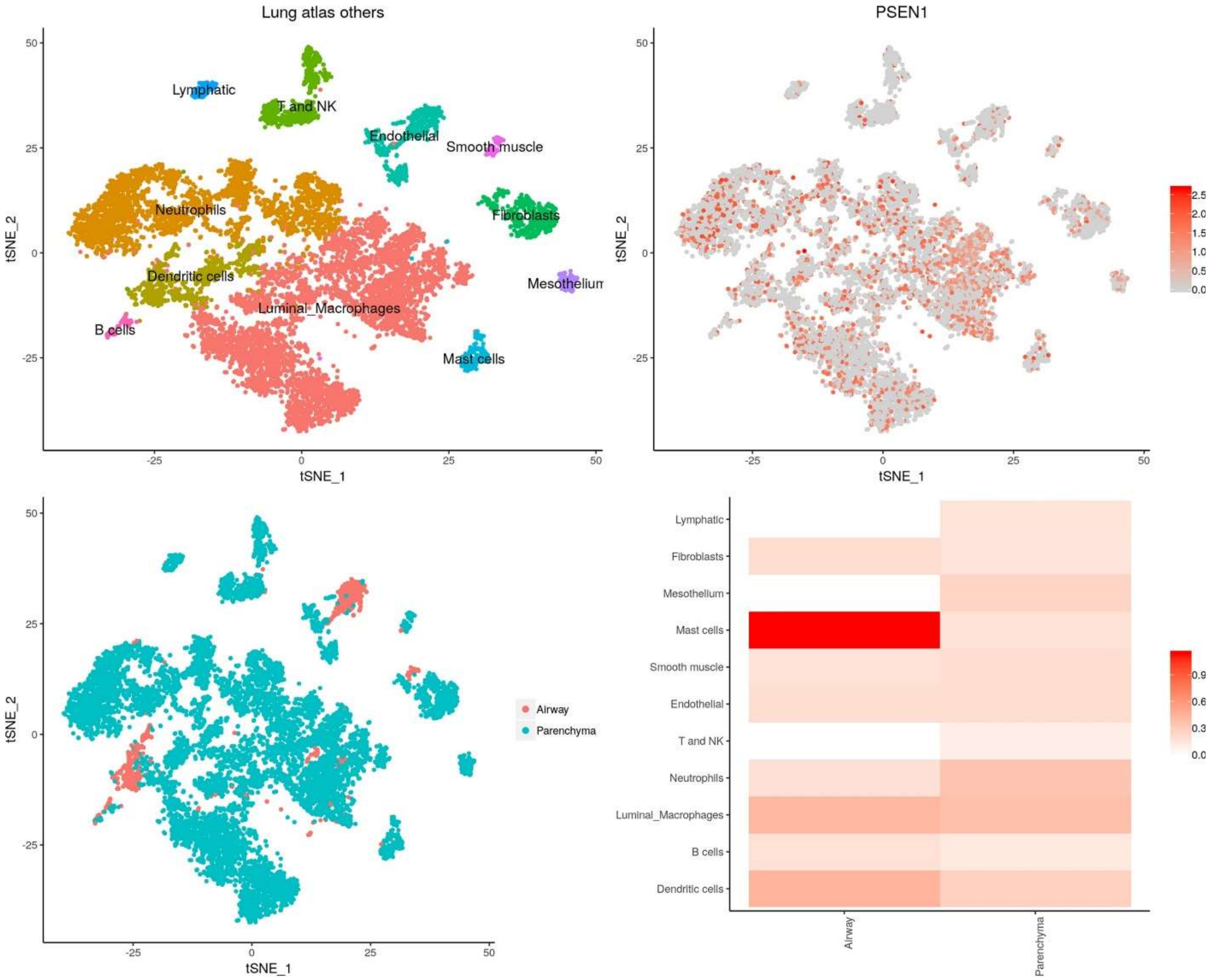

Figure S12b. PSEN1, non-epithelium.

Upper left: Displaying unbiased clustering of different non-epithelial cell types in lung tissue; Upper right: t-SNE plot showing the expression of PSEN1 in different cluster of cells; Lower left: Different cell types in upper airway and parenchymal lung tissue; Lower right: t-SNE plot showing the expression of PSEN1 in upper airway and parenchymal lung tissue.

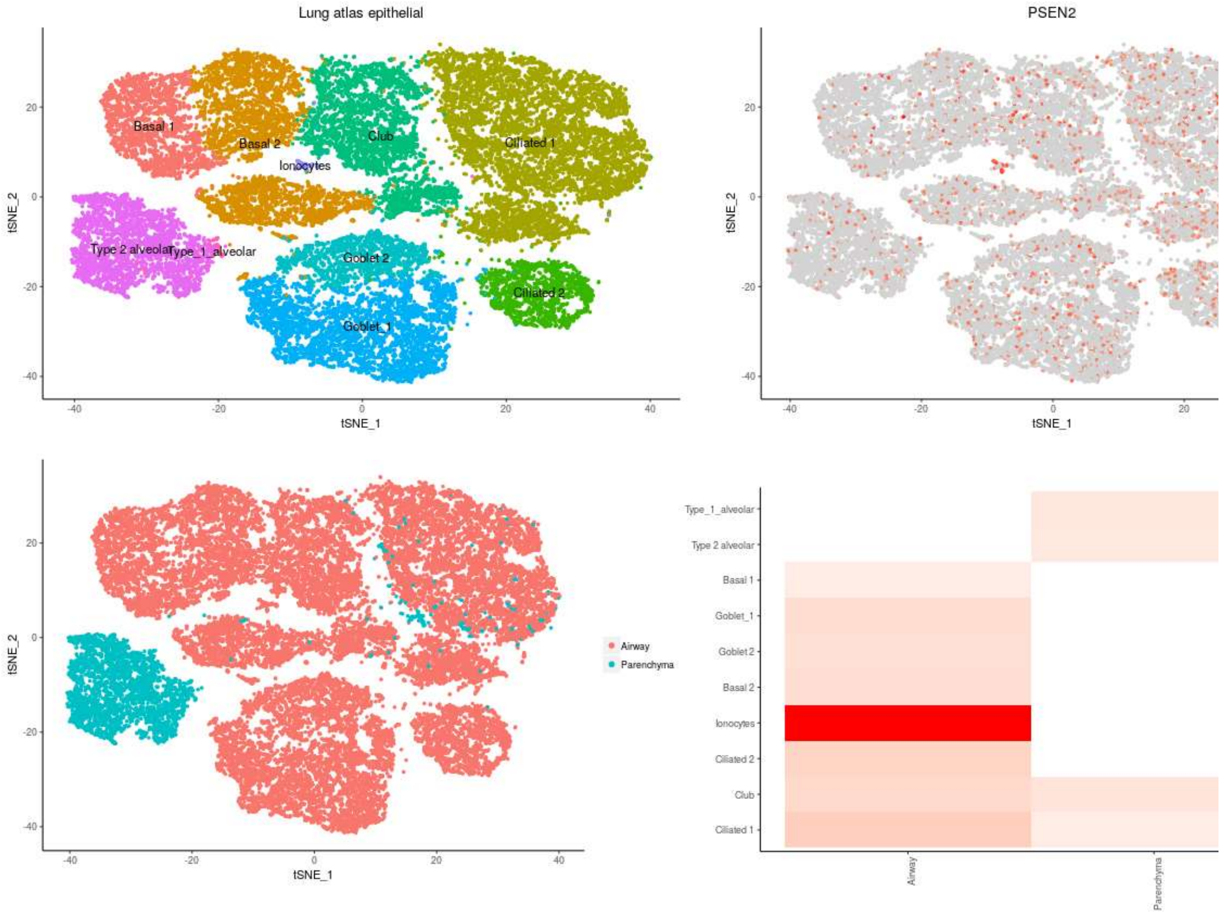

Figure S13a. PSEN2, lung epithelium.

Upper left: Displaying unbiased clustering of different cell types in lung tissue; Upper right: t-SNE plot showing the expression of PSEN2 in different cluster of cells; Lower left: Different cell types in upper airway and parenchymal lung tissue; Lower right: t-SNE plot showing the expression of PSEN2 in upper airway and parenchymal lung tissue.

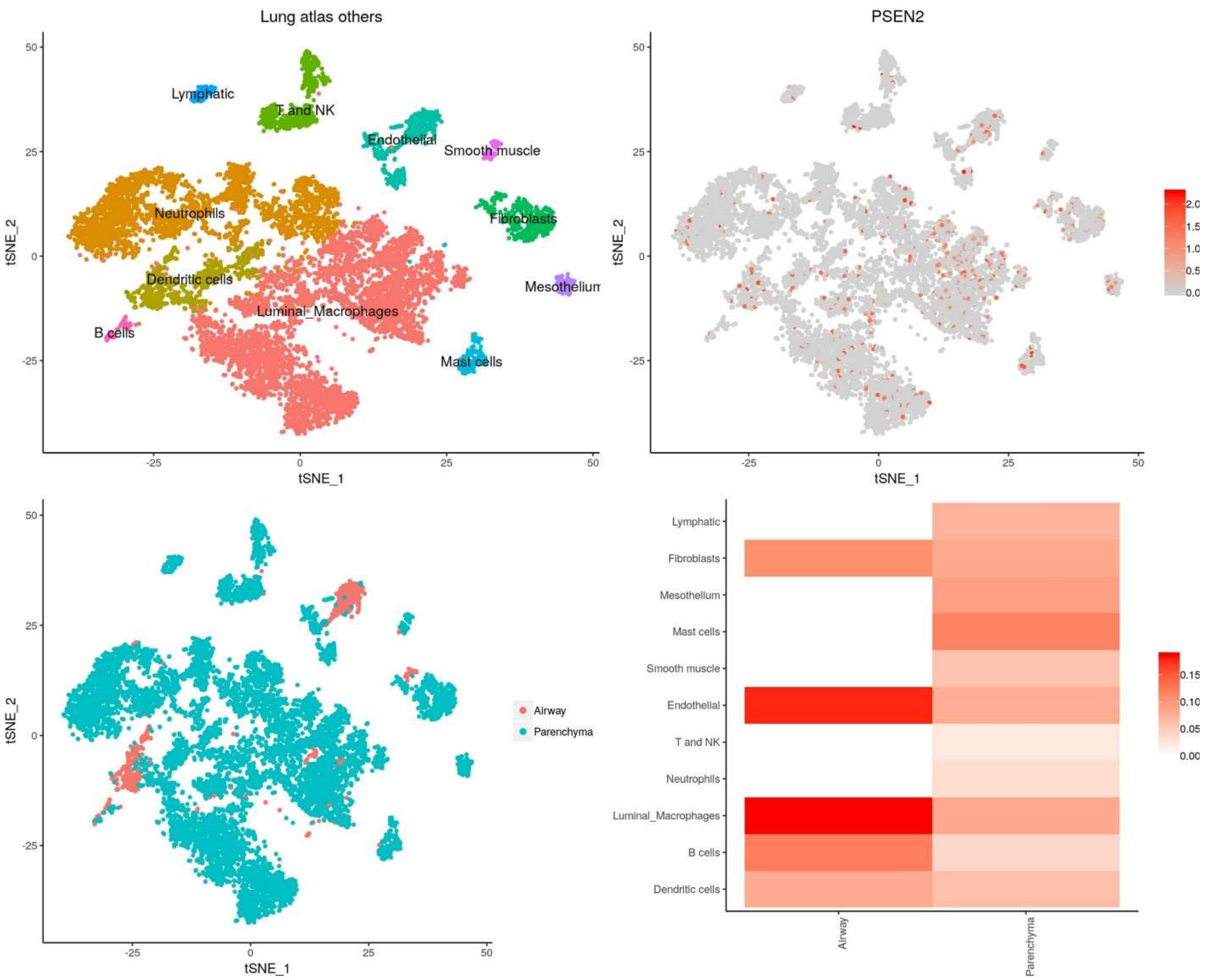

Figure S13b. PSEN2, non-epithelium.

Upper left: Displaying unbiased clustering of different non-epithelial cell types in lung tissue; Upper right: t-SNE plot showing the expression of PSEN2 in different cluster of cells; Lower left: Different cell types in upper airway and parenchymal lung tissue; Lower right: t-SNE plot showing the expression of PSEN2 in upper airway and parenchymal lung tissue.

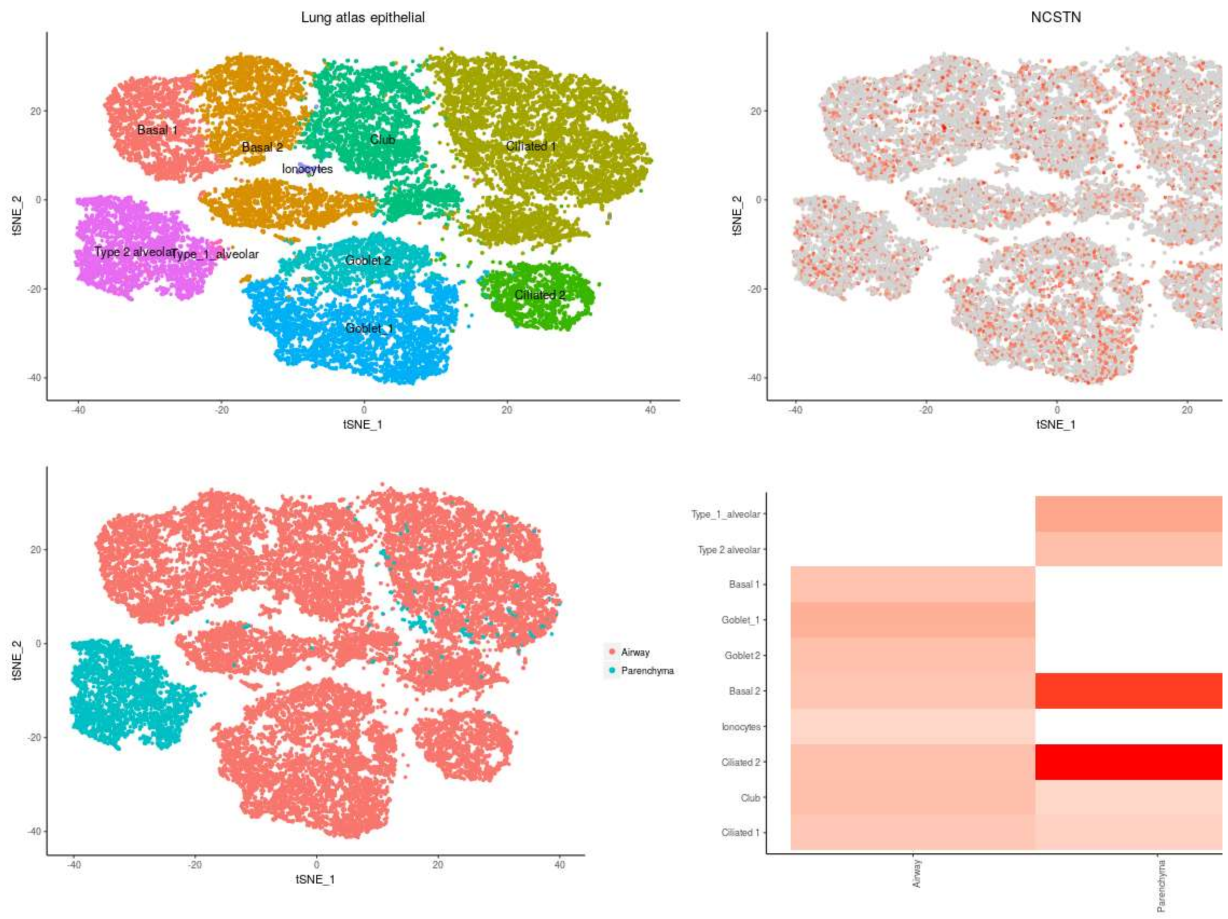

Figure S14a. NCSTN, lung epithelium.

Upper left: Displaying unbiased clustering of different cell types in lung tissue; Upper right: t-SNE plot showing the expression of NCSTN in different cluster of cells; Lower left: Different cell types in upper airway and parenchymal lung tissue; Lower right: t-SNE plot showing the expression of NCSTN in upper airway and parenchymal lung tissue.

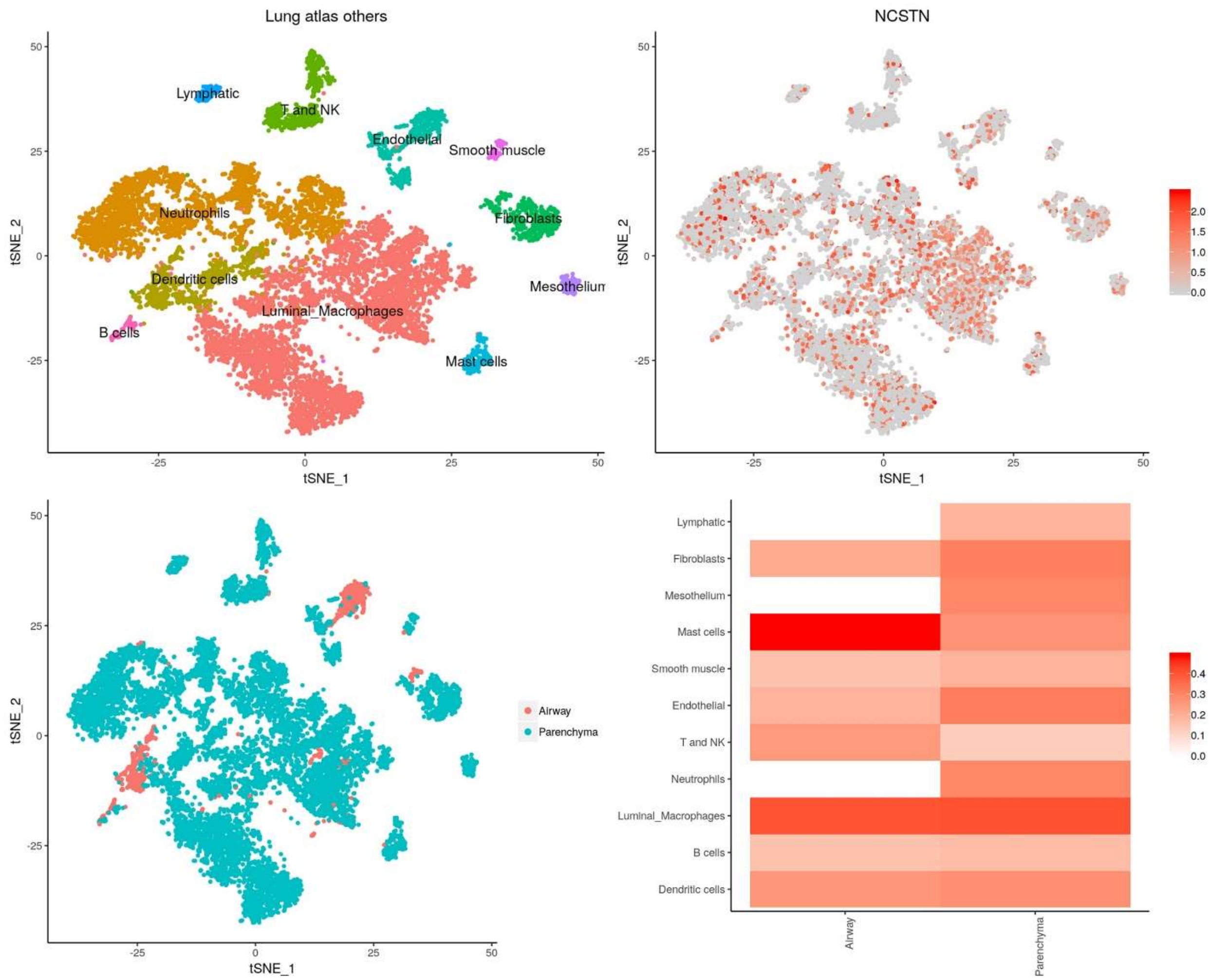

Figure S14b. NCSTN, non-epithelium.

Upper left: Displaying unbiased clustering of different non-epithelial cell types in lung tissue; Upper right: t-SNE plot showing the expression of NCSTN in different cluster of cells; Lower left: Different cell types in upper airway and parenchymal lung tissue; Lower right: t-SNE plot showing the expression of NCSTN in upper airway and parenchymal lung tissue.

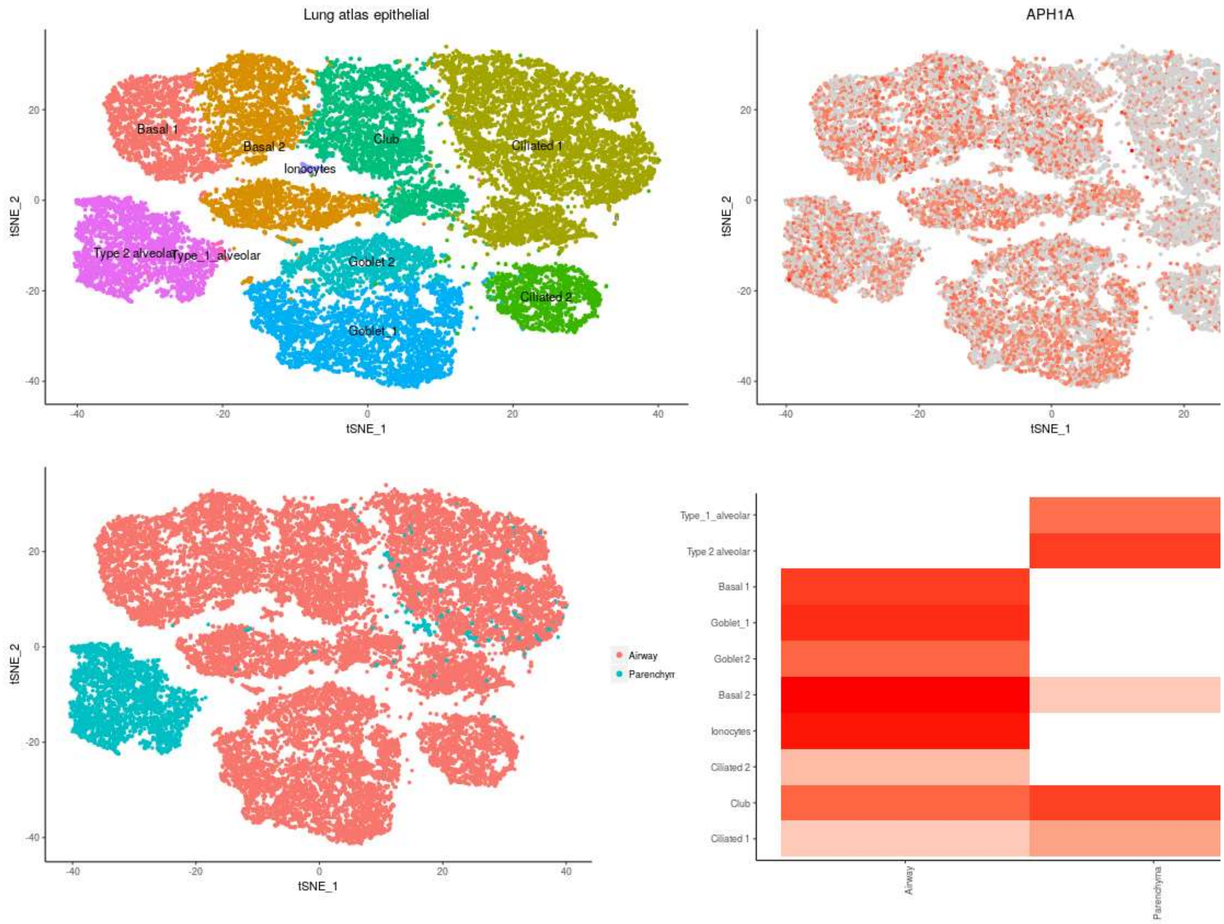

Figures S15a. APH1A, lung epithelium.

Upper left: Displaying unbiased clustering of different cell types in lung tissue; Upper right: t-SNE plot showing the expression of APH1A in different cluster of cells; Lower left: Different cell types in upper airway and parenchymal lung tissue; Lower right: t-SNE plot showing the expression of APH1A in upper airway and parenchymal lung tissue.

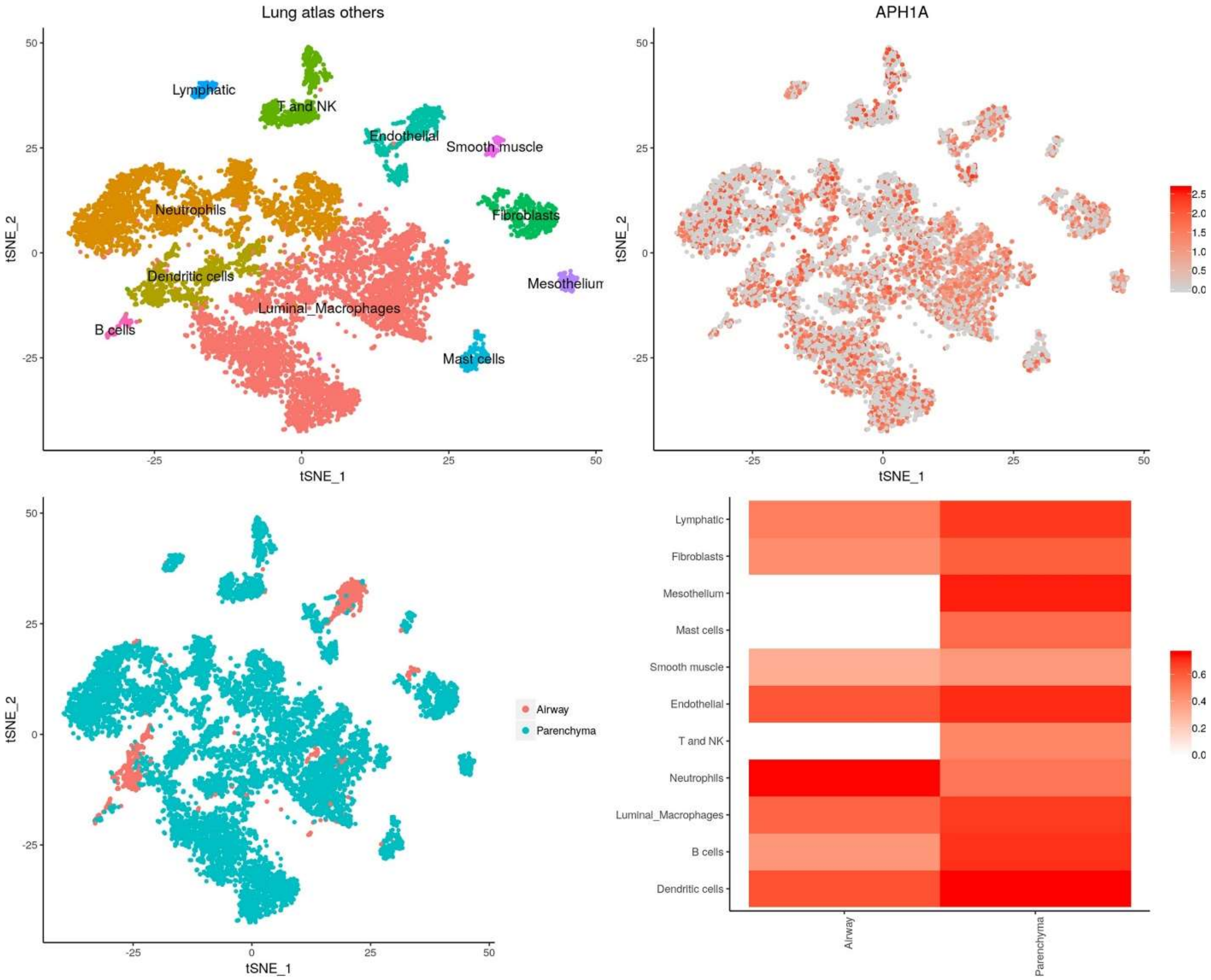

Figure S15b. APH1A, non-epithelium.

Upper left: Displaying unbiased clustering of different non-epithelial cell types in lung tissue; Upper right: t-SNE plot showing the expression of APH1A in different cluster of cells; Lower left: Different cell types in upper airway and parenchymal lung tissue; Lower right: t-SNE plot showing the expression of APH1A in upper airway and parenchymal lung tissue.

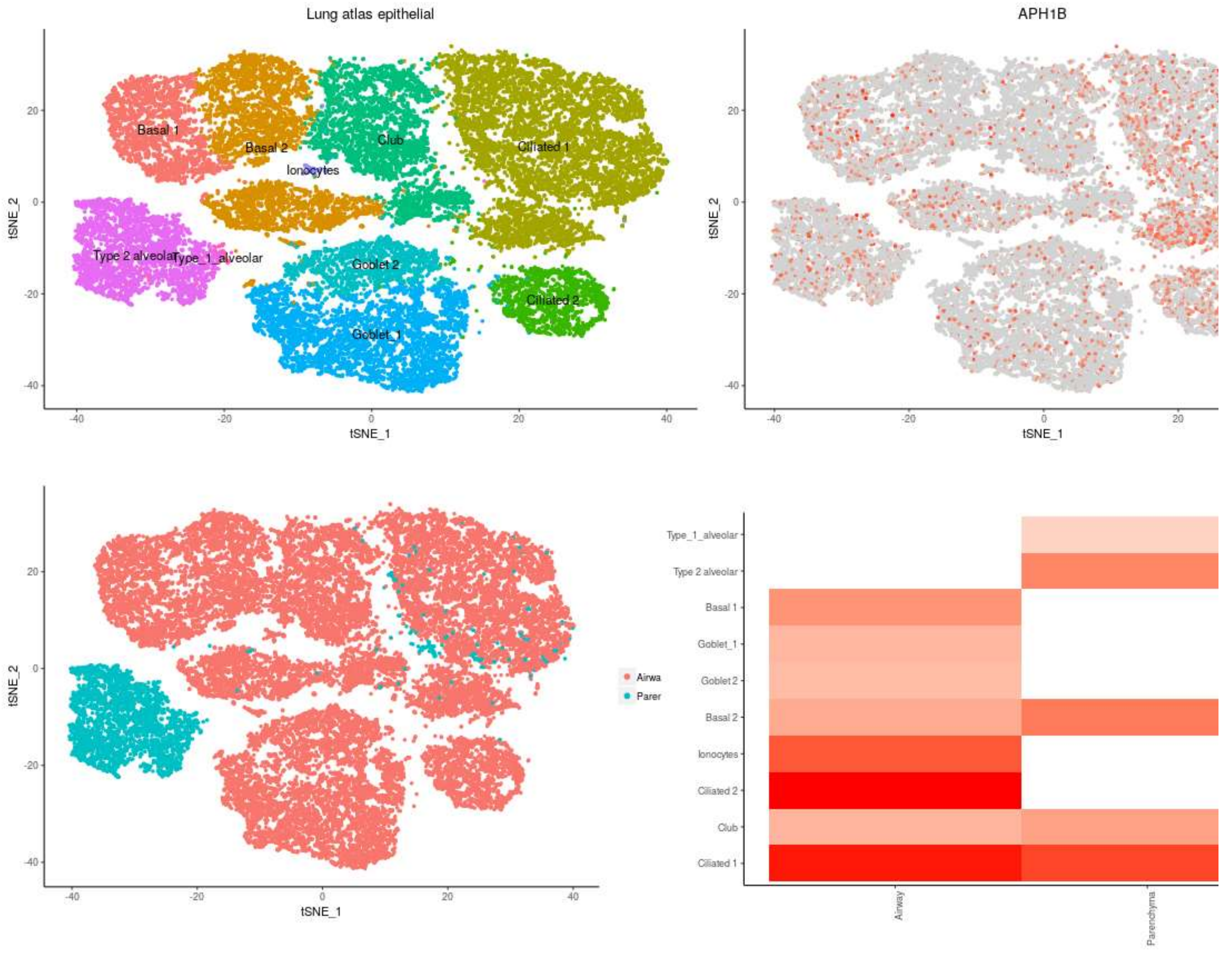

Figure S16a. APH1B, lung epithelium.

Upper left: Displaying unbiased clustering of different cell types in lung tissue; Upper right: t-SNE plot showing the expression of APH1B in different cluster of cells; Lower left: Different cell types in upper airway and parenchymal lung tissue; Lower right: t-SNE plot showing the expression of APH1B in upper airway and parenchymal lung tissue.

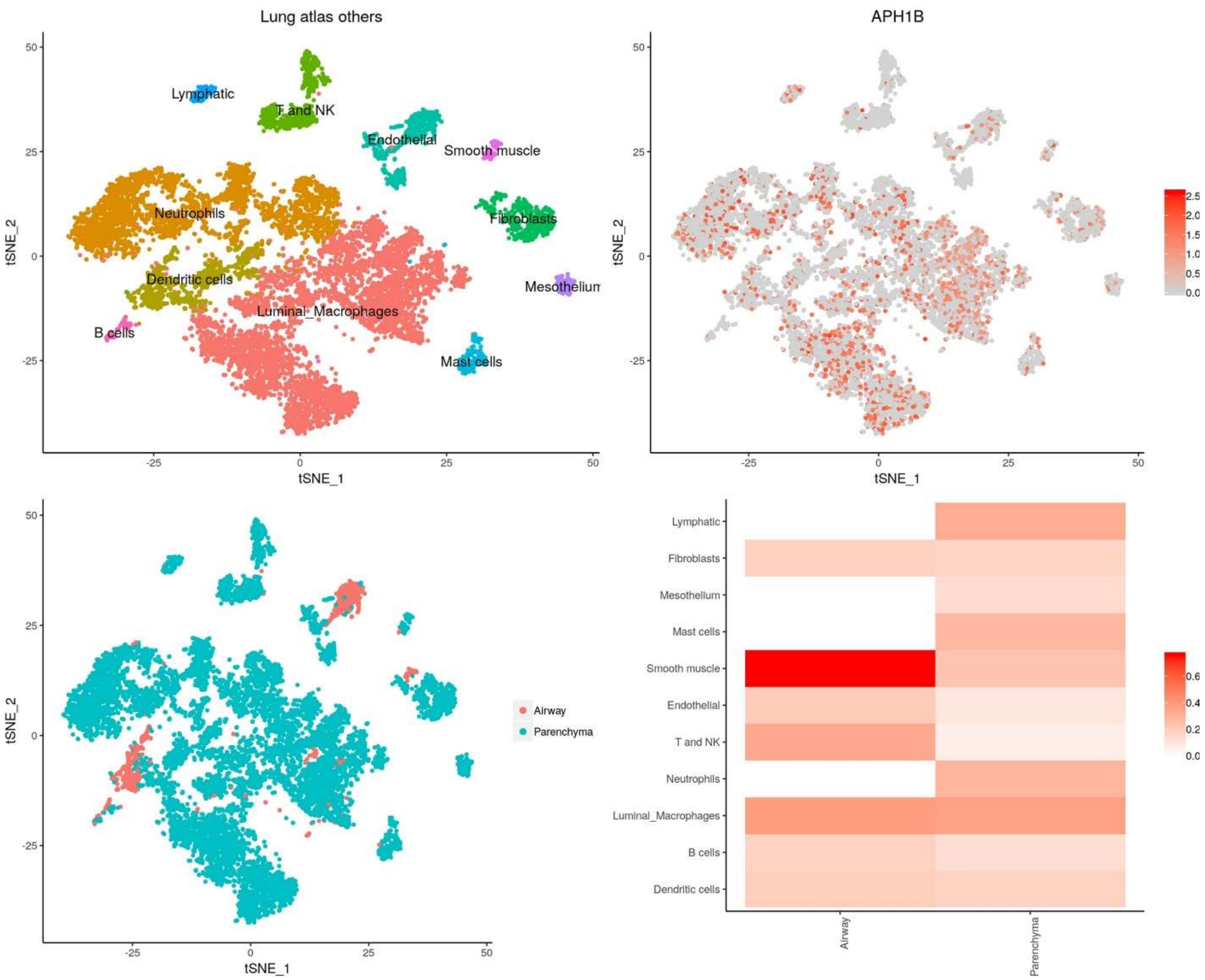

Figure S16b. APH1B, non-epithelium.

Upper left: Displaying unbiased clustering of different non-epithelial cell types in lung tissue; Upper right: t-SNE plot showing the expression of APH1B in different cluster of cells; Lower left: Different cell types in upper airway and parenchymal lung tissue; Lower right: t-SNE plot showing the expression of APH1B in upper airway and parenchymal lung tissue.

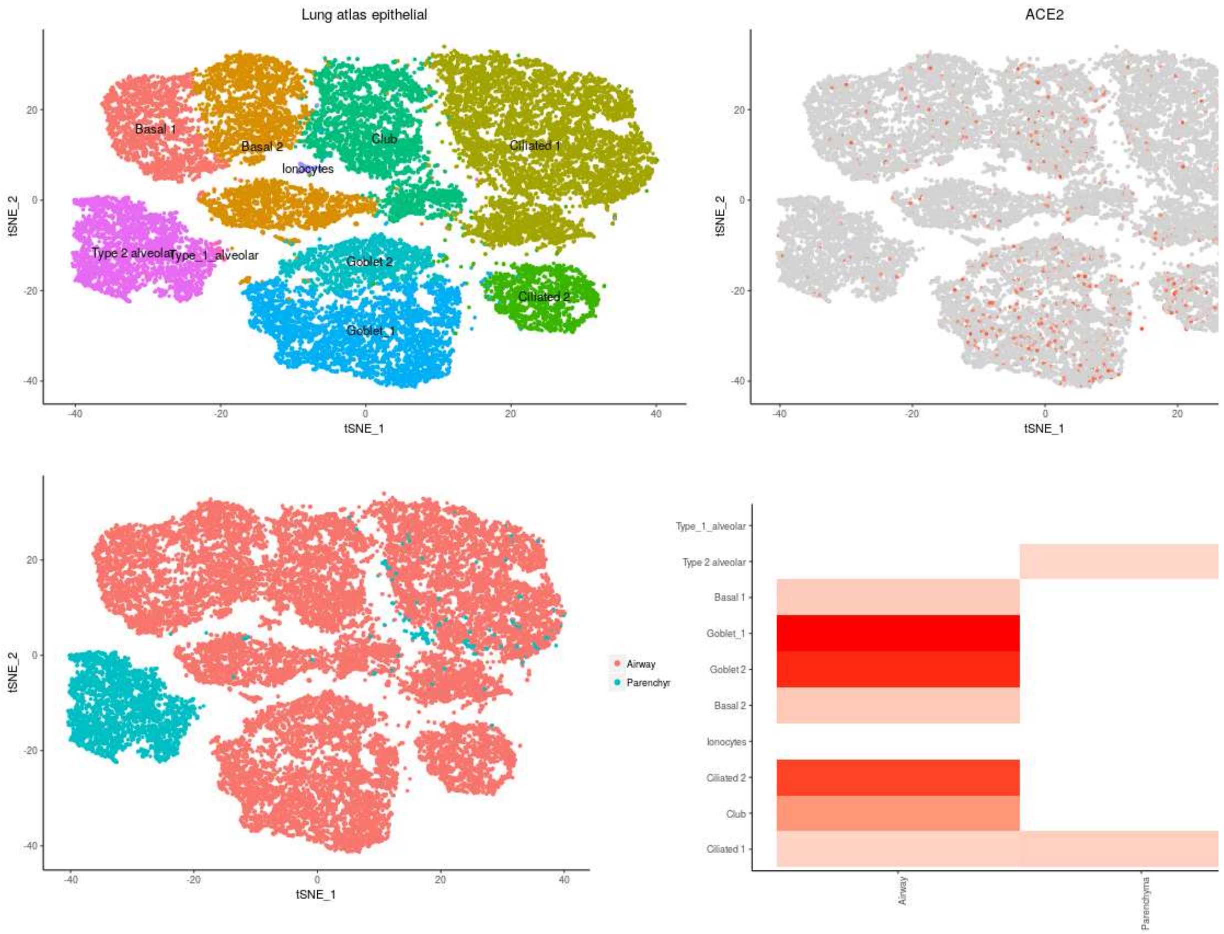

Figure S17a. ACE2, lung epithelium.

Upper left: Displaying unbiased clustering of different cell types in lung tissue; Upper right: t-SNE plot showing the expression of ACE2 in different cluster of cells; Lower left: Different cell types in upper airway and parenchymal lung tissue; Lower right: t-SNE plot showing the expression of ACE2 in upper airway and parenchymal lung tissue.

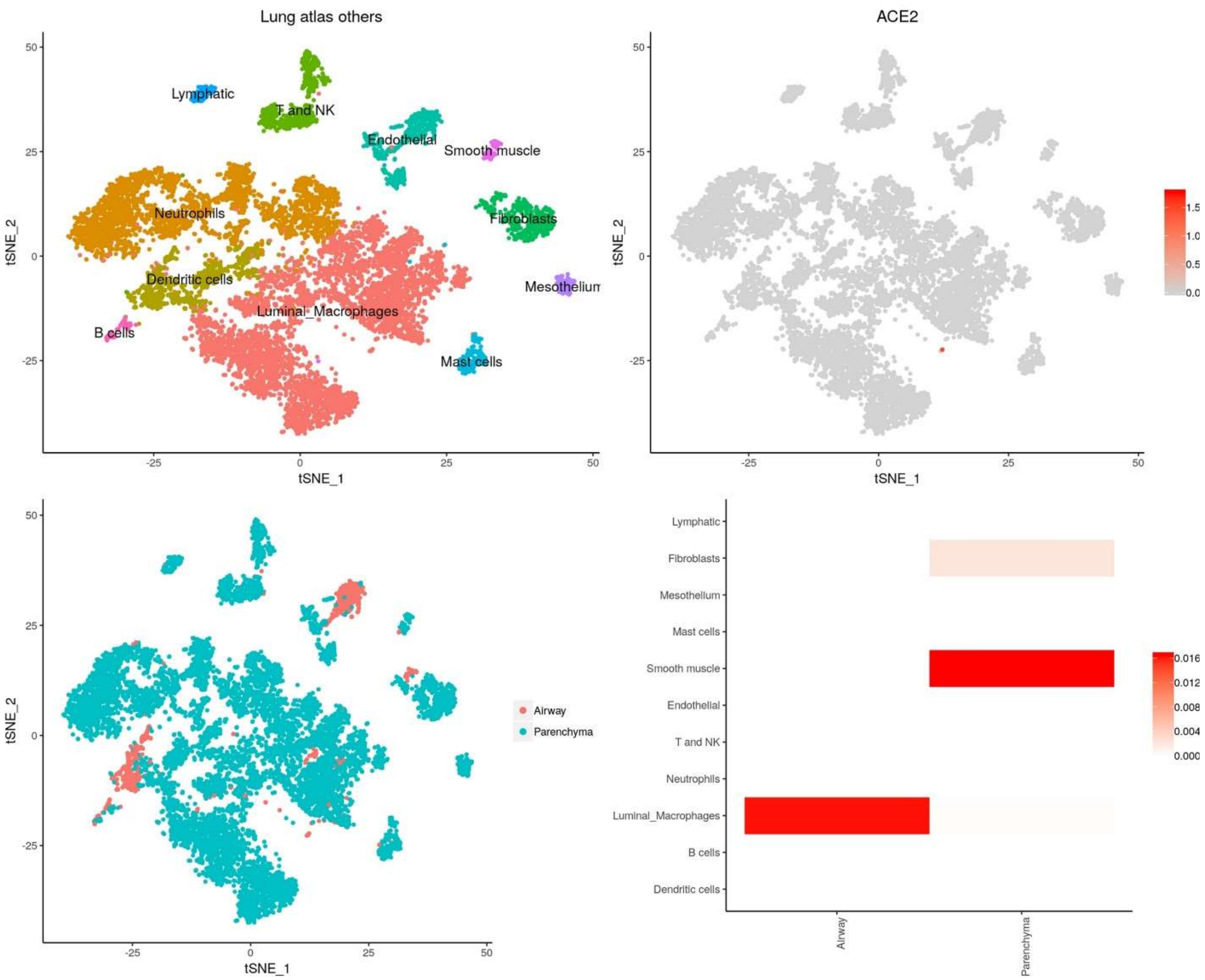

Figure S17b. ACE2, non-epithelium.

Upper left: Displaying unbiased clustering of different non-epithelial cell types in lung tissue; Upper right: t-SNE plot showing the expression of ACE2 in different cluster of cells; Lower left: Different cell types in upper airway and parenchymal lung tissue; Lower right: t-SNE plot showing the expression of ACE2 in upper airway and parenchymal lung tissue.

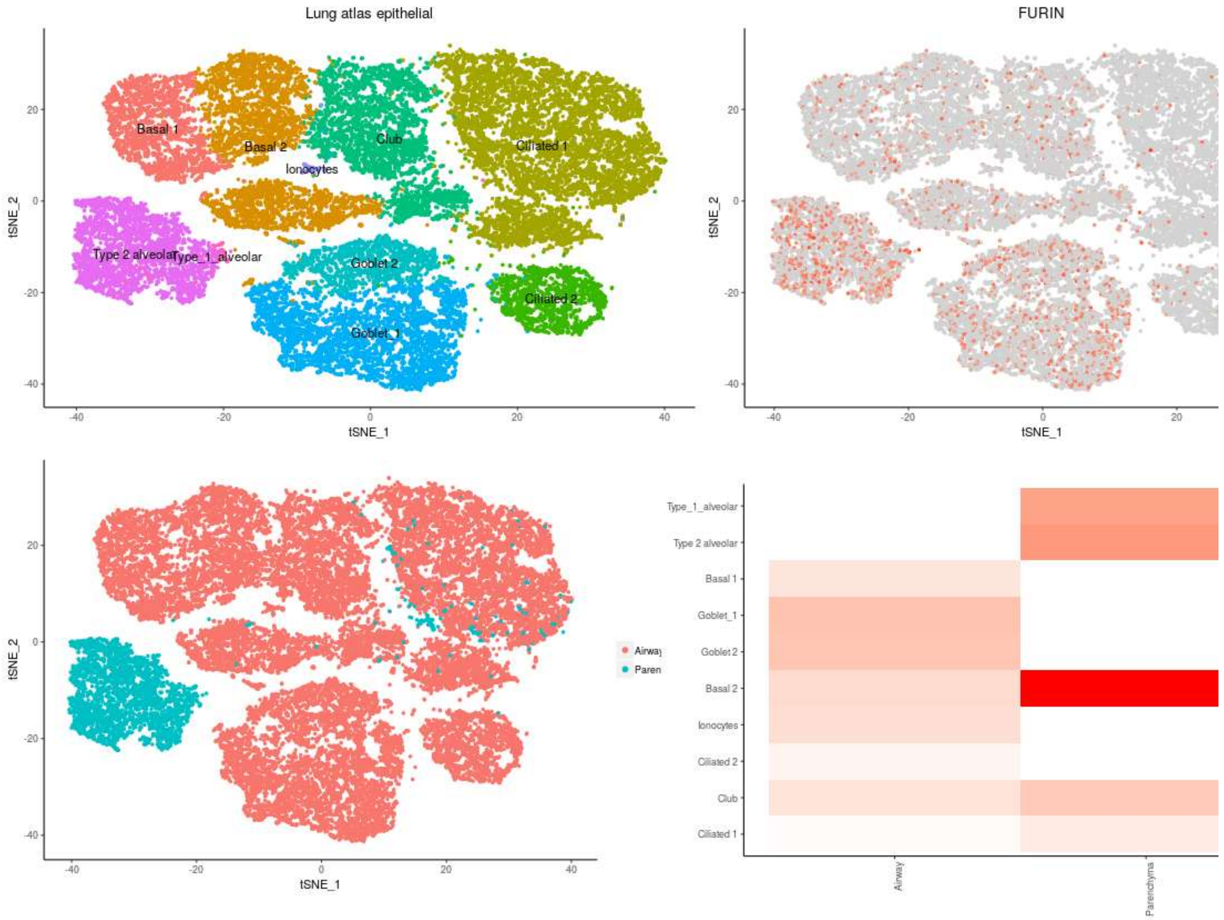

Figure S18a. FURIN, lung epithelium.

Upper left: Displaying unbiased clustering of different cell types in lung tissue; Upper right: t-SNE plot showing the expression of FURIN in different cluster of cells; Lower left: Different cell types in upper airway and parenchymal lung tissue; Lower right: t-SNE plot showing the expression of FURIN in upper airway and parenchymal lung tissue.

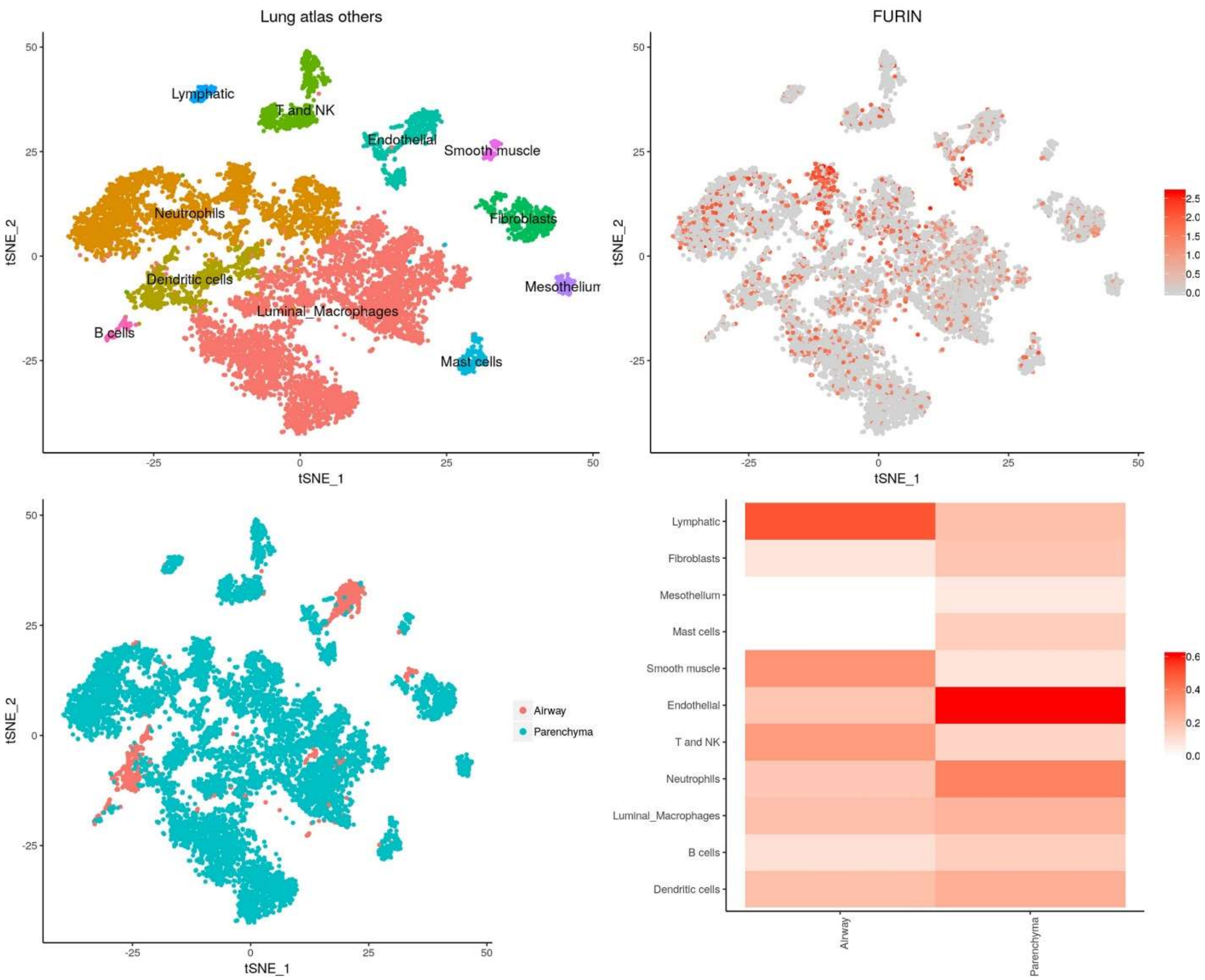

Figure S18b. FURIN, non-epithelium.

Upper left: Displaying unbiased clustering of different non-epithelial cell types in lung tissue; Upper right: t-SNE plot showing the expression of FURIN in different cluster of cells; Lower left: Different cell types in upper airway and parenchymal lung tissue; Lower right: t-SNE plot showing the expression of FURIN in upper airway and parenchymal lung tissue.

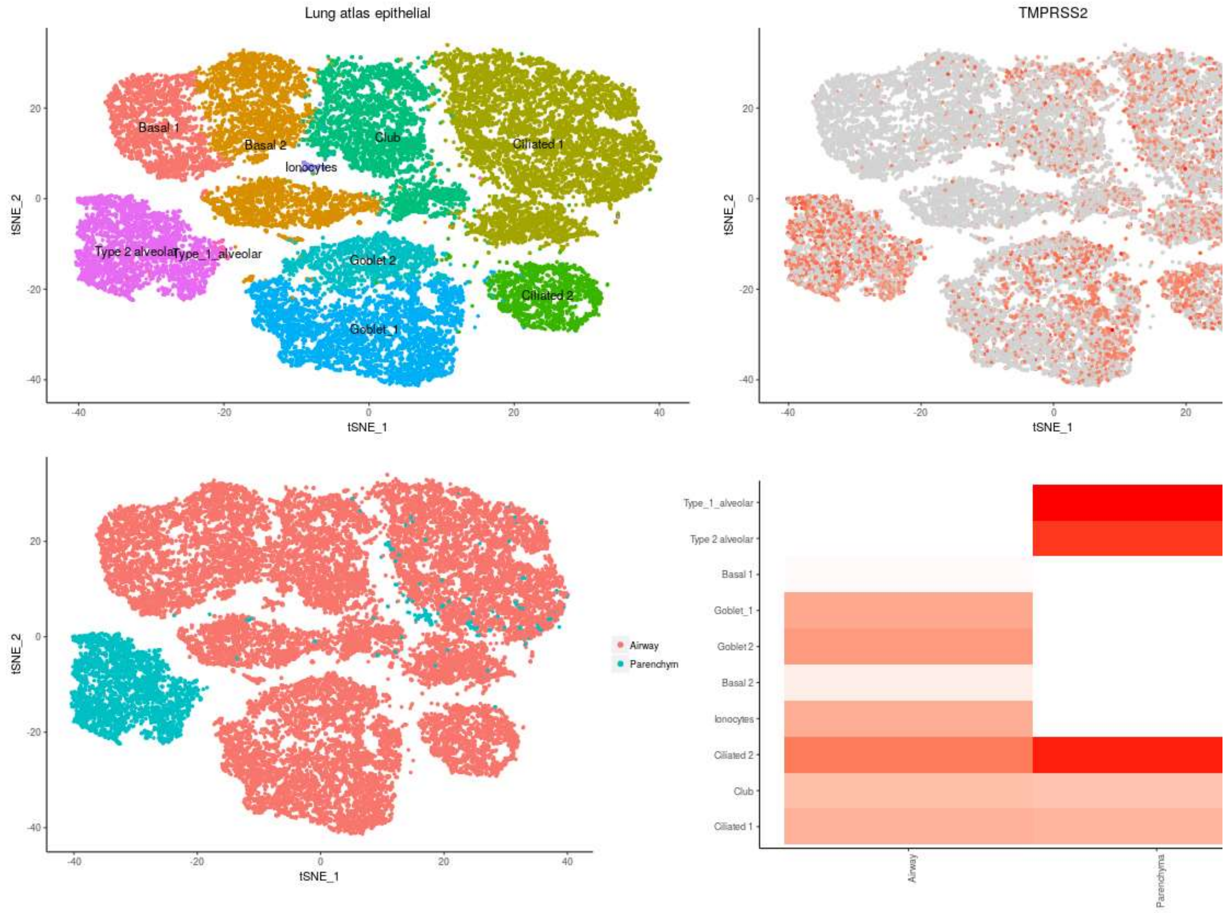

Figure S19a. TMPRSS2, lung epithelium.

Upper left: Displaying unbiased clustering of different cell types in lung tissue; Upper right: t-SNE plot showing the expression of TMPRSS2 in different cluster of cells; Lower left: Different cell types in upper airway and parenchymal lung tissue; Lower right: t-SNE plot showing the expression of TMPRSS2 in upper airway and parenchymal lung tissue.

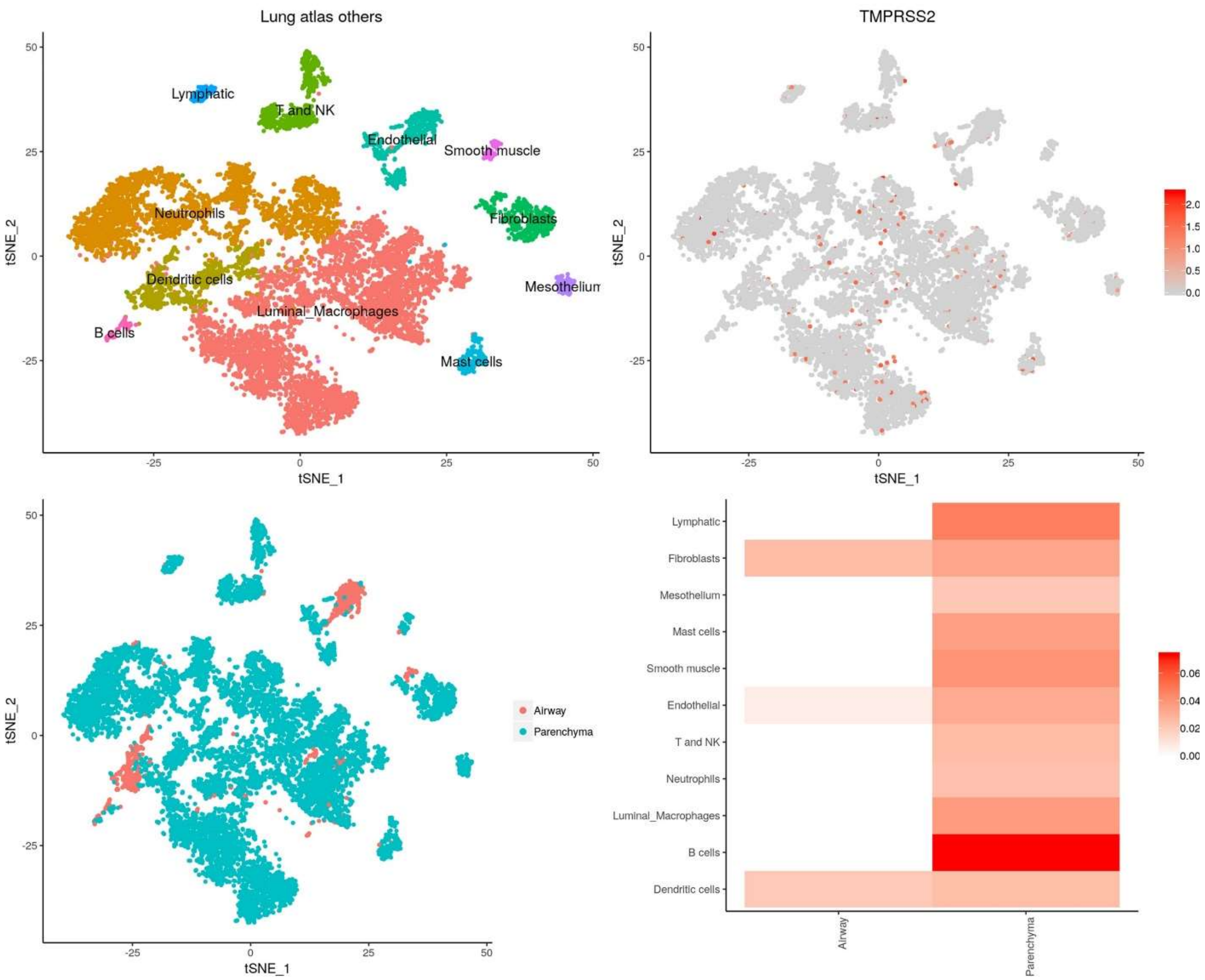

Figure S19b. TMPRSS2, non-epithelium.

Upper left: Displaying unbiased clustering of different non-epithelial cell types in lung tissue; Upper right: t-SNE plot showing the expression of TMPRSS2 in different cluster of cells; Lower left: Different cell types in upper airway and parenchymal lung tissue; Lower right: t-SNE plot showing the expression of TMPRSS2 in upper airway and parenchymal lung tissue.

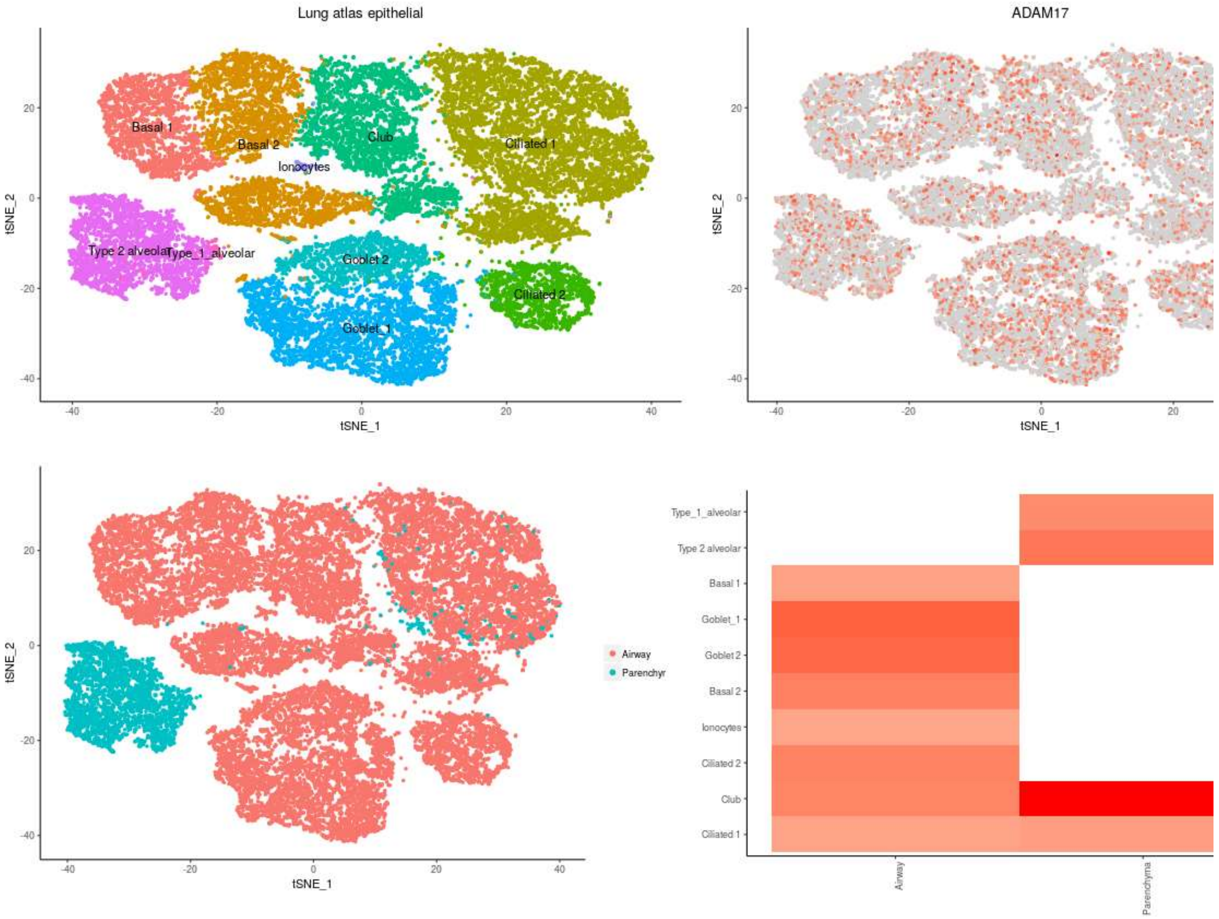

Figure S20a. ADAM17, lung epithelium.

Upper left: Displaying unbiased clustering of different cell types in lung tissue; Upper right: t-SNE plot showing the expression of ADAM17 in different cluster of cells; Lower left: Different cell types in upper airway and parenchymal lung tissue; Lower right: t-SNE plot showing the expression of ADAM17 in upper airway and parenchymal lung tissue.

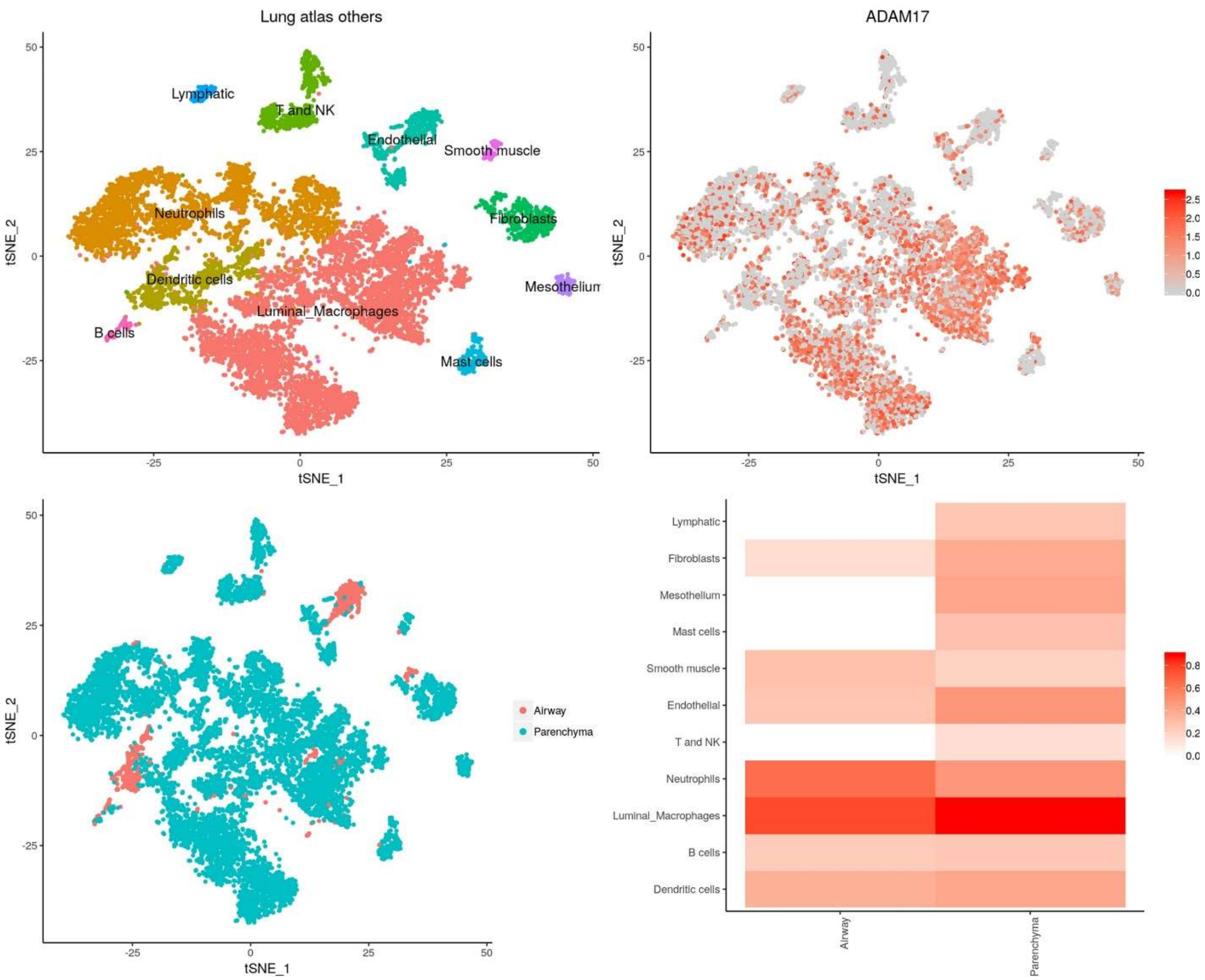

Figure S20b. ADAM17, non-epithelium.

Upper left: Displaying unbiased clustering of different non-epithelial cell types in lung tissue; Upper right: t-SNE plot showing the expression of ADAM17 in different cluster of cells; Lower left: Different cell types in upper airway and parenchymal lung tissue; Lower right: t-SNE plot showing the expression of ADAM17 in upper airway and parenchymal lung tissue.

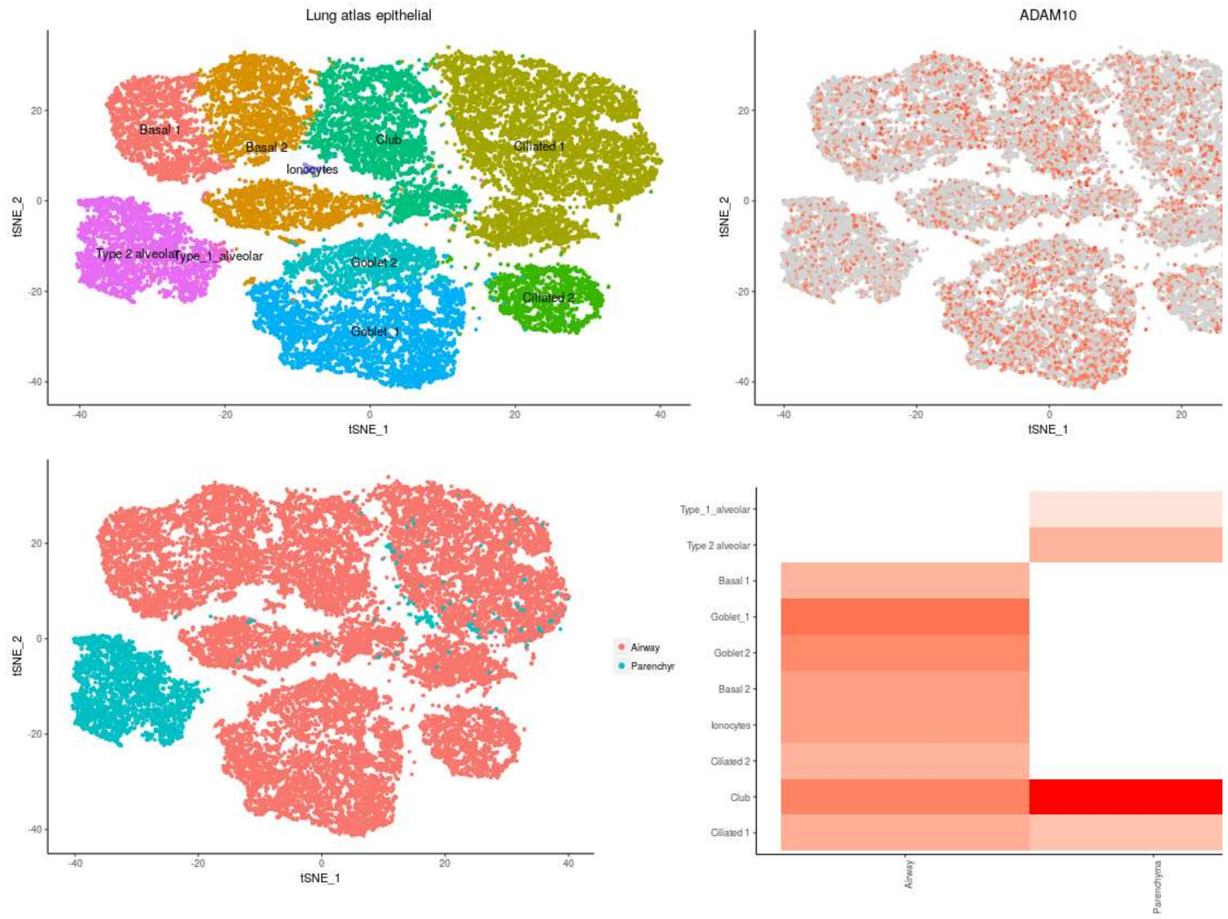

Figure S21a. ADAM10, lung epithelium.

Upper left: Displaying unbiased clustering of different cell types in lung tissue; Upper right: t-SNE plot showing the expression of ADAM10 in different cluster of cells; Lower left: Different cell types in upper airway and parenchymal lung tissue; Lower right: t-SNE plot showing the expression of ADAM10 in upper airway and parenchymal lung tissue.

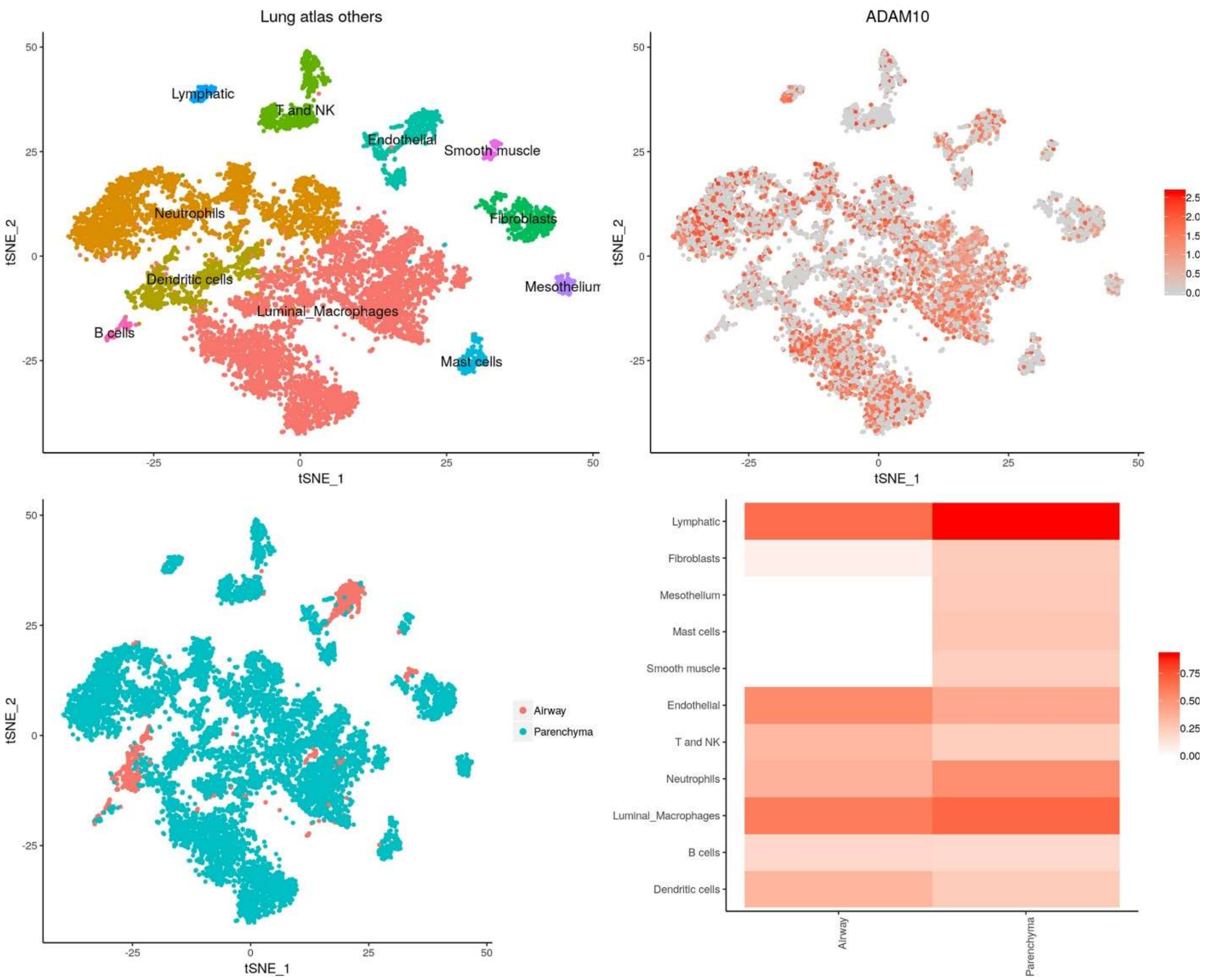

Figure S21b. ADAM10, non-epithelium.

Upper left: Displaying unbiased clustering of different non-epithelial cell types in lung tissue; Upper right: t-SNE plot showing the expression of ADAM10 in different cluster of cells; Lower left: Different cell types in upper airway and parenchymal lung tissue; Lower right: t-SNE plot showing the expression of ADAM10 in upper airway and parenchymal lung tissue.

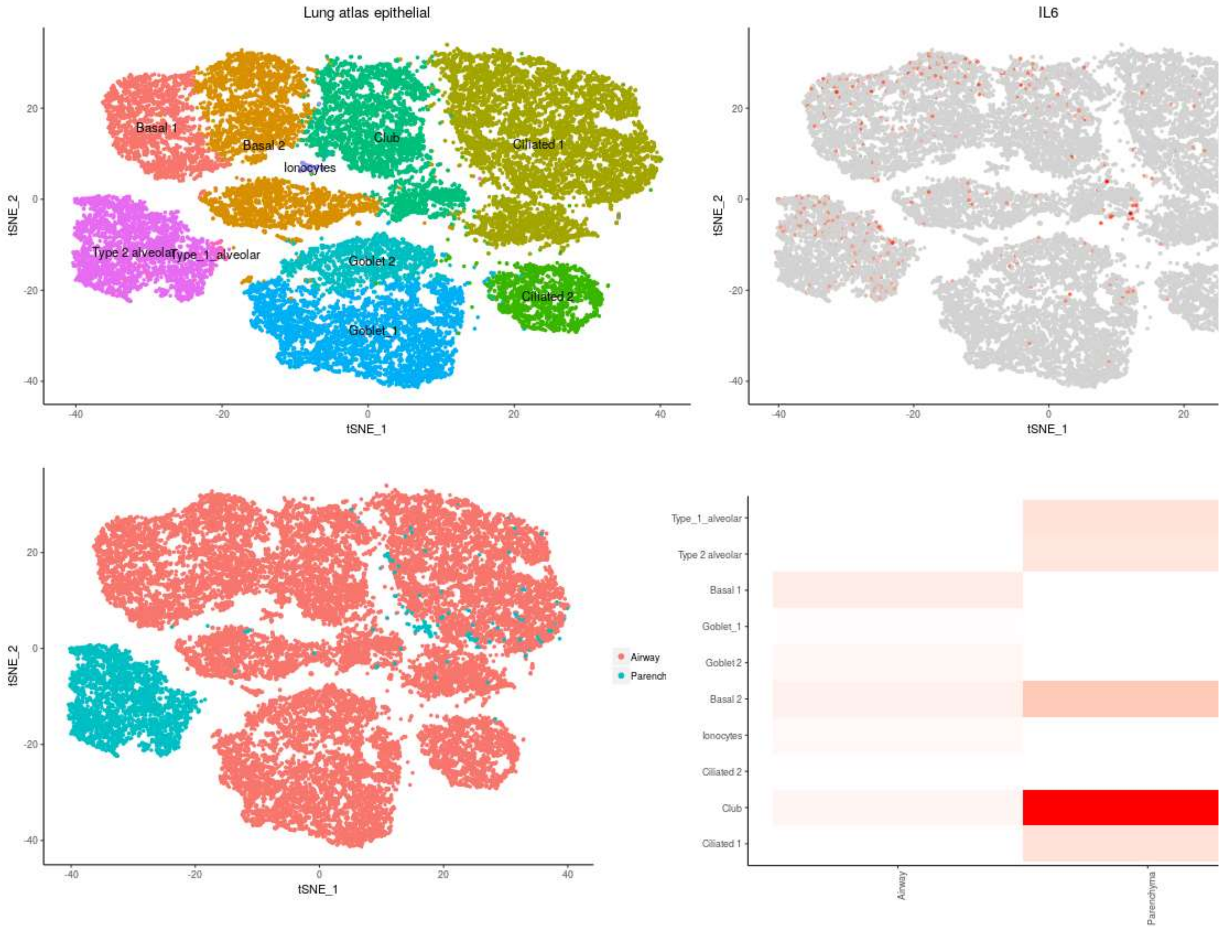

Figure S22a. IL6, lung epithelium.

Upper left: Displaying unbiased clustering of different cell types in lung tissue; Upper right: t-SNE plot showing the expression of IL6 in different cluster of cells; Lower left: Different cell types in upper airway and parenchymal lung tissue; Lower right: t-SNE plot showing the expression of IL6 in upper airway and parenchymal lung tissue.

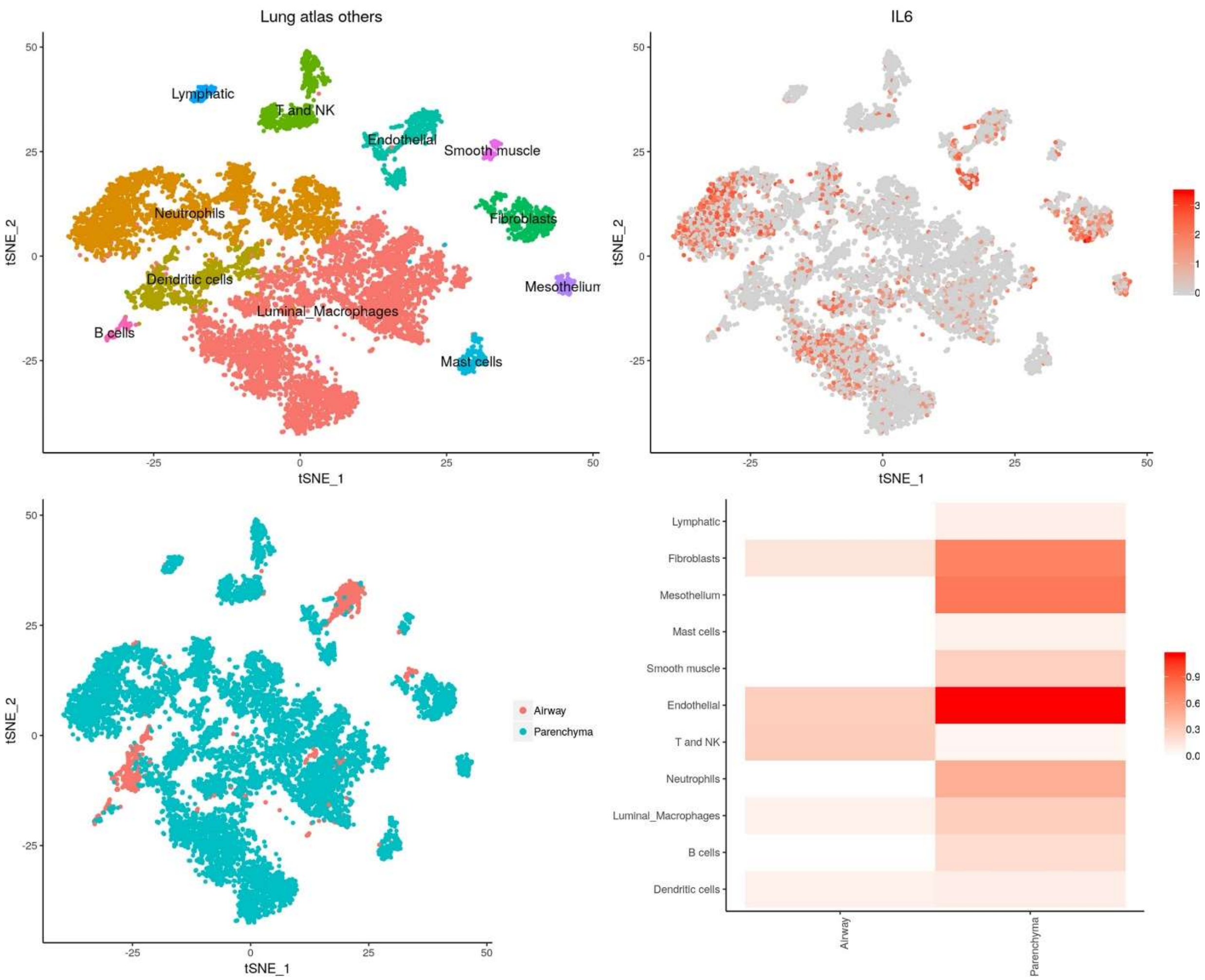

Figure S22b. IL6, non-epithelium.

Upper left: Displaying unbiased clustering of different non-epithelial cell types in lung tissue; Upper right: t-SNE plot showing the expression of IL6 in different cluster of cells; Lower left: Different cell types in upper airway and parenchymal lung tissue; Lower right: t-SNE plot showing the expression of IL6 in upper airway and parenchymal lung tissue.

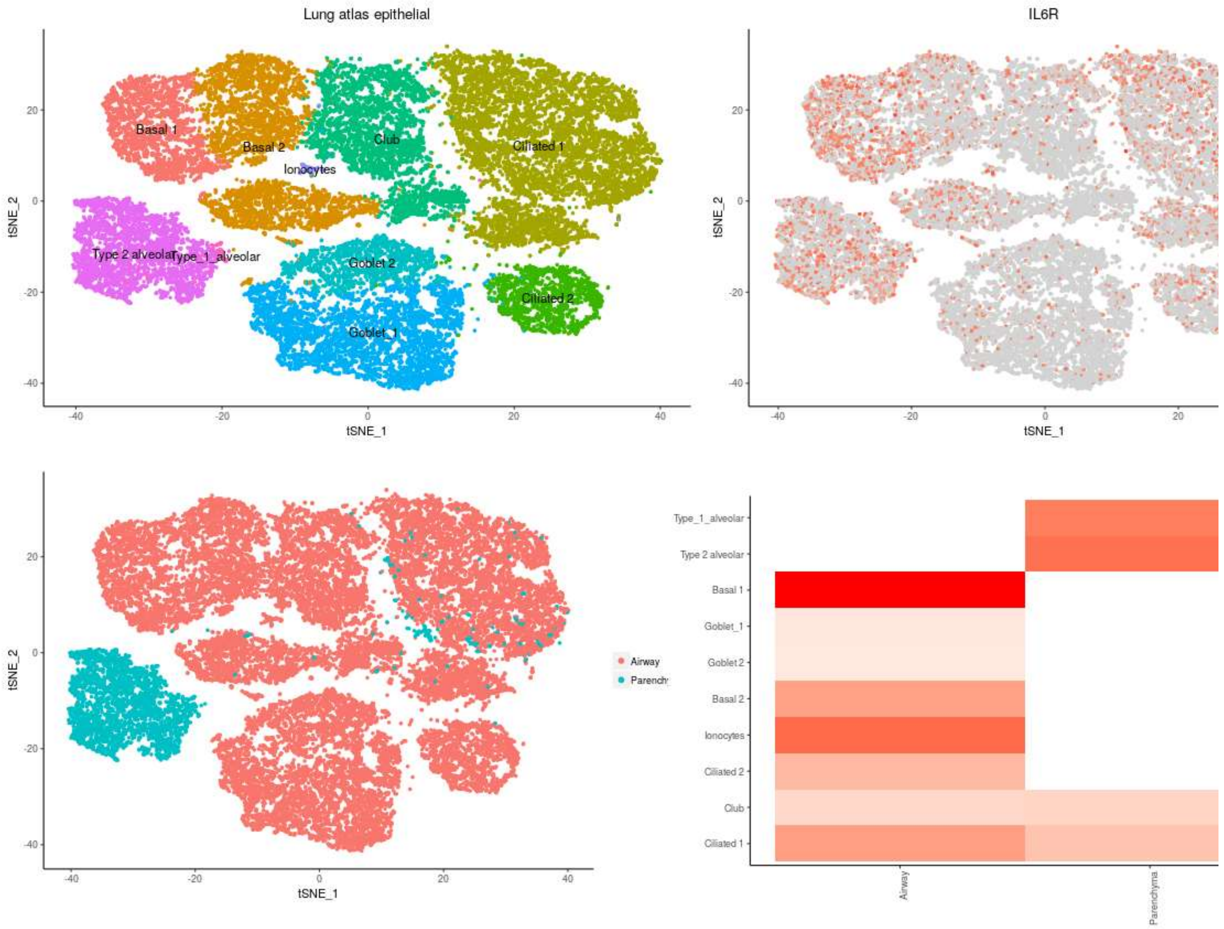

Figure S23a. IL6R, lung epithelium.

Upper left: Displaying unbiased clustering of different cell types in lung tissue; Upper right: t-SNE plot showing the expression of IL6R in different cluster of cells; Lower left: Different cell types in upper airway and parenchymal lung tissue; Lower right: t-SNE plot showing the expression of IL6R in upper airway and parenchymal lung tissue.

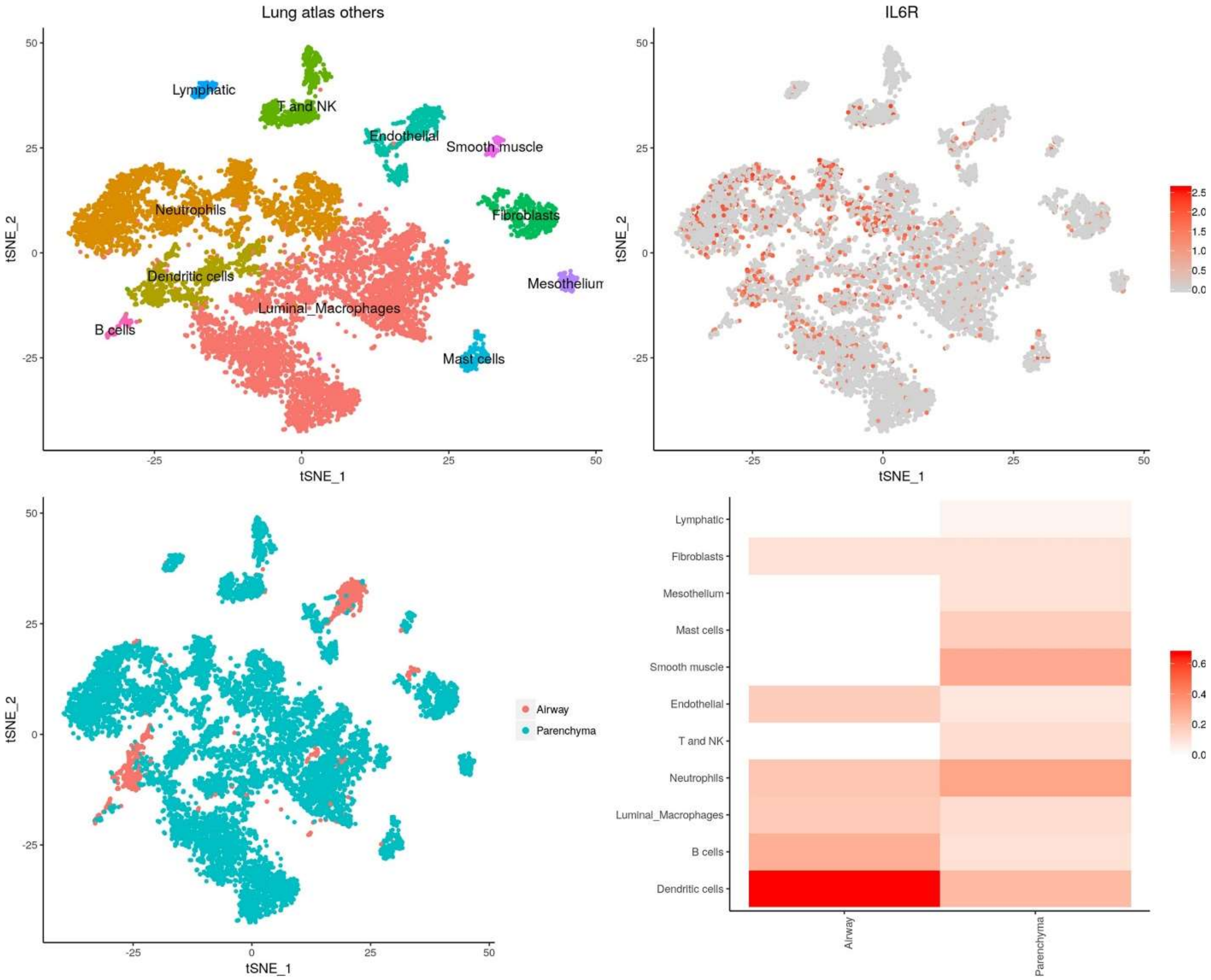

Figure S23b. IL6R, non-epithelium.

Upper left: Displaying unbiased clustering of different non-epithelial cell types in lung tissue; Upper right: t-SNE plot showing the expression of IL6R in different cluster of cells; Lower left: Different cell types in upper airway and parenchymal lung tissue; Lower right: t-SNE plot showing the expression of IL6R in upper airway and parenchymal lung tissue.

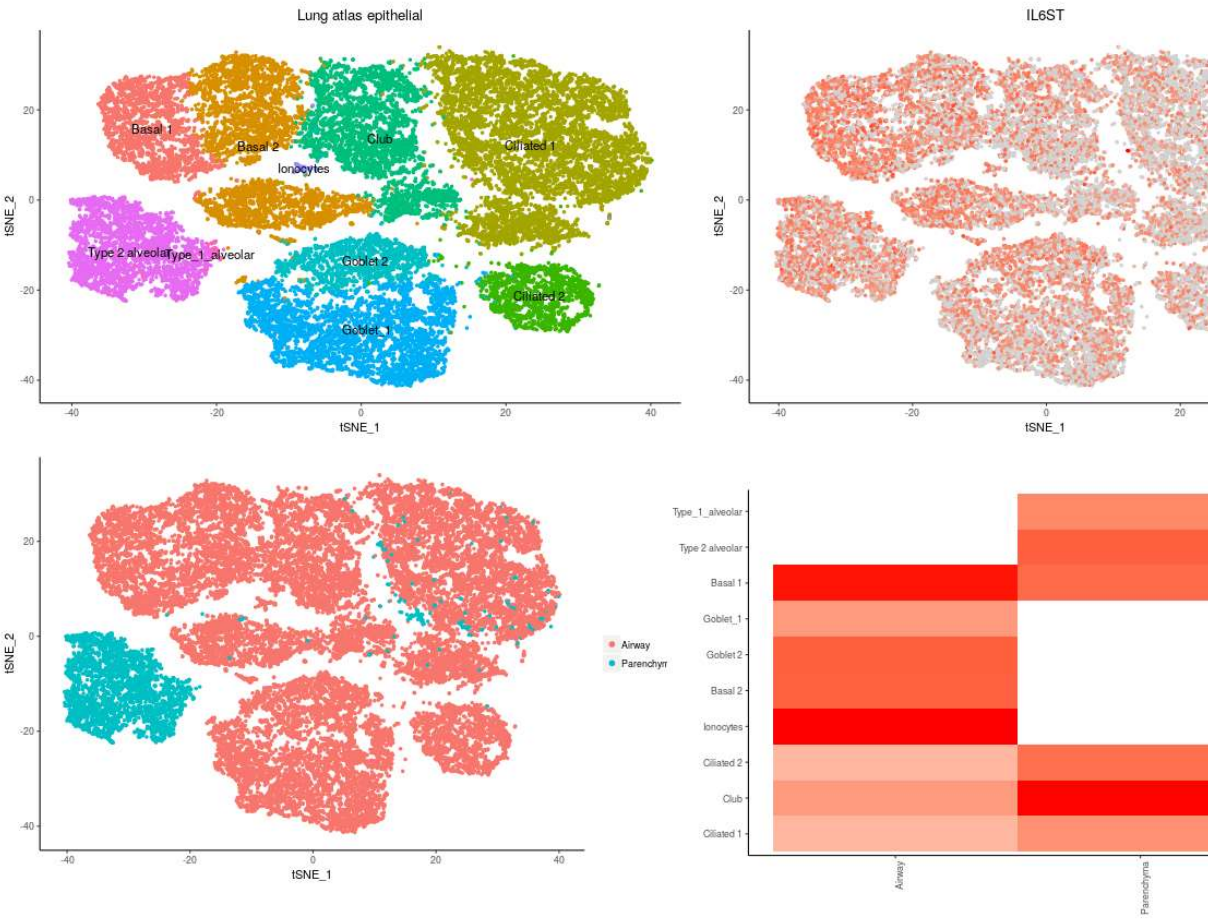

Figure S24a. IL6ST, lung epithelium.

Upper left: Displaying unbiased clustering of different cell types in lung tissue; Upper right: t-SNE plot showing the expression of IL6ST in different cluster of cells; Lower left: Different cell types in upper airway and parenchymal lung tissue; Lower right: t-SNE plot showing the expression of IL6ST in upper airway and parenchymal lung tissue.

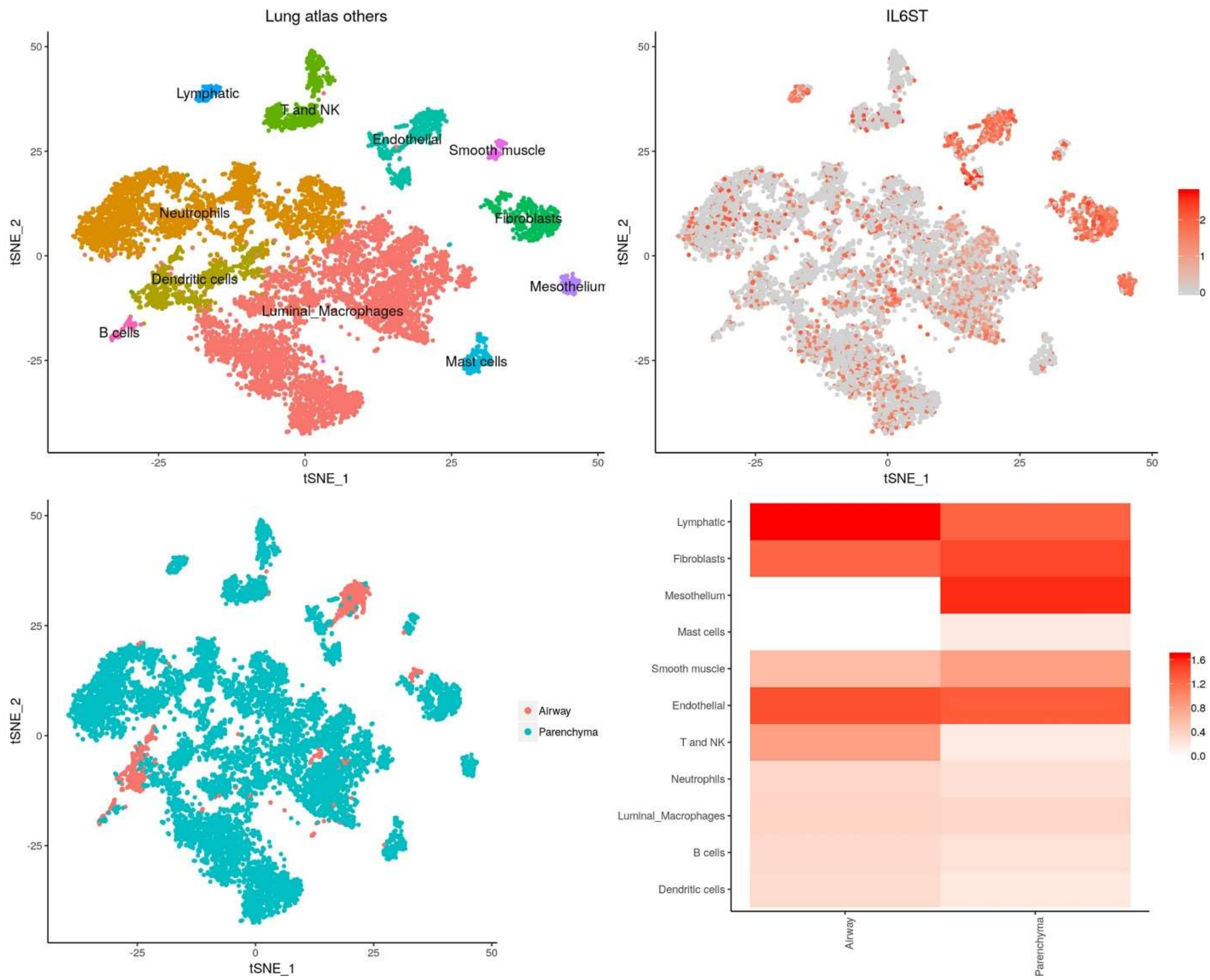

Figure S24b. IL6ST, non-epithelium.

Upper left: Displaying unbiased clustering of different non-epithelial cell types in lung tissue; Upper right: t-SNE plot showing the expression of IL6ST in different cluster of cells; Lower left: Different cell types in upper airway and parenchymal lung tissue; Lower right: t-SNE plot showing the expression of IL6ST in upper airway and parenchymal lung tissue.

References

Szklarczyk, D., Gable, A. L., Lyon, D., Junge, A., Wyder, S., Huerta-Cepas, J., et al. (2019). STRING v11: Protein-protein association networks with increased coverage, supporting functional discovery in genome-wide experimental datasets. *Nucleic Acids Res.* 47, D607–D613. doi:10.1093/nar/gky1131.

Vieira Braga, F. A., Kar, G., Berg, M., Carpaij, O. A., Polanski, K., Simon, L. M., et al. (2019). A cellular census of human lungs identifies novel cell states in health and in asthma. *Nat. Med.* 25, 1153–1163. doi:10.1038/s41591-019-0468-5.
